# Diminutive fleet-footed tyrannosauroid narrows the 70-million-year gap in the North American fossil record

**DOI:** 10.1038/s42003-019-0308-7

**Published:** 2019-02-21

**Authors:** Lindsay E. Zanno, Ryan T. Tucker, Aurore Canoville, Haviv M. Avrahami, Terry A. Gates, Peter J. Makovicky

**Affiliations:** 1 Paleontology, North Carolina Museum of Natural Sciences, 11W. Jones, St. Raleigh, NC 27601 USA; 2 Department of Biological Sciences, North Carolina State University, 100 Brooks Ave., Raleigh, NC 27607 USA; 3 Section of Earth Sciences, Field Museum of Natural History, 1400S. Lake Shore Dr., Chicago, IL 60605 USA; 4 Department of Earth Sciences, Stellenbosch University, Private Bag X1 Matieland, Stellenbosch, 7602 South Africa

## Abstract

To date, eco-evolutionary dynamics in the ascent of tyrannosauroids to top predator roles have been obscured by a 70-million-year gap in the North American (NA) record. Here we report discovery of the oldest Cretaceous NA tyrannosauroid, extending the lineage by ~15 million years. The new taxon—*Moros intrepidus* gen. et sp. nov.—is represented by a hind limb from an individual nearing skeletal maturity at 6–7 years. With a ~1.2-m limb length and 78-kg mass, *M*. *intrepidus* ranks among the smallest Cretaceous tyrannosauroids, restricting the window for rapid mass increases preceding the appearance of colossal eutyrannosaurs. Phylogenetic affinity with Asian taxa supports transcontinental interchange as the means by which iconic biotas of the terminal Cretaceous were established in NA. The unexpectedly diminutive and highly cursorial bauplan of NA’s earliest Cretaceous tyrannosauroids reveals an evolutionary strategy reliant on speed and small size during their prolonged stint as marginal predators.

## Abstract (web summary)

Lindsay Zanno et al. report the discovery of a new tyrannosaur that helps to fill in a 70 million year gap in the fossil record. This new species reveals that the earliest North American tyrannosaurs relied on speed and small body size to survive and that apex predator status and large body sizes were not reached until much later in their evolutionary history.

## Introduction

During the terminal Cretaceous, tyrannosauroids reigned supreme within terrestrial ecosystems of the Northern Hemisphere, evolving a lethal combination of colossal size^1^, exceptional bite forces^2^, accelerated growth rates^3^, and adept sensory systems^4,5^ that would redefine top predator guilds leading up to the end-Cretaceous mass extinction event ~66 million years ago. Yet, for the vast majority of their more than 100 million-year-long evolutionary history, tyrannosauroids were small bodied, subordinate hunters, evolving in the shadow of archaic lineages that were already established at the top of the food chain^6,7^. The timing and tempo of trophic restructuring within Late Cretaceous terrestrial ecosystems, including the extinction of allosaurian megapredators and the ascent of tyrannosauroids to top predator roles, are poorly constrained. Teasing out these eco-evolutionary dynamics requires a comprehensive record of tyrannosauroid biodiversity (currently upwards of 30 valid species) and relatively contiguous spatio-temporal data, particularly during the poorly sampled interval spanning the late Early to early Late Cretaceous, or ‘mid-Cretaceous’, during which time this niche shift and associated anatomical transformations are hypothesized to have occurred^6–8^.

Whereas the Cretaceous record of Asian tyrannosauroids is rapidly growing (at minimum represented by six pre-Campanian species), and significant progress has recently been made understanding the timing of major evolutionary transitions within Asian representatives of the clade^5,9^, the pre-Campanian record in North America (NA) has remained entirely devoid of diagnostic body fossils. Widely represented by multiple taxa in Late Jurassic, the species record of tyrannosauroids on the North American continent goes dark across the Jurassic–Cretaceous boundary and does not reappear until the latest Cretaceous (ca. 81 Ma)^8^—a gap in the fossil record spanning 70 million years, and one that directly precedes the ‘sudden’ appearance and rapid radiation of large-bodied eutyrannosaurians as apex predators within Campanian ecosystems^8^.

Here we report the discovery of a new, diminutive tyrannosauroid, *Moros intrepidus* gen. et sp. nov., as well as isolated premaxillary teeth sharing synapomorphies with Asian taxa, from Cenomanian-aged terrestrial deposits of western NA. The new taxon represents the oldest Cretaceous diagnostic tyrannosauroid on the continent, extending the definitive record of this clade by ~15 million years, and providing a key datum for untangling evolutionary, paleobiogeographic, and ecological dynamics during a turbulent interval in Earth’s history.

## Results

### Systematic paleontology

Dinosauria Owen, 1842

Theropoda Marsh, 1881

Coelurosauria von Huene, 1914

Tyrannosauroidea Osborn, 1905

Tyrannosauroidea indet.

**Referred materials** NCSM 33393, NCSM 33276 isolated tyrannosauroid premaxillary teeth.

**Fig. 1:**
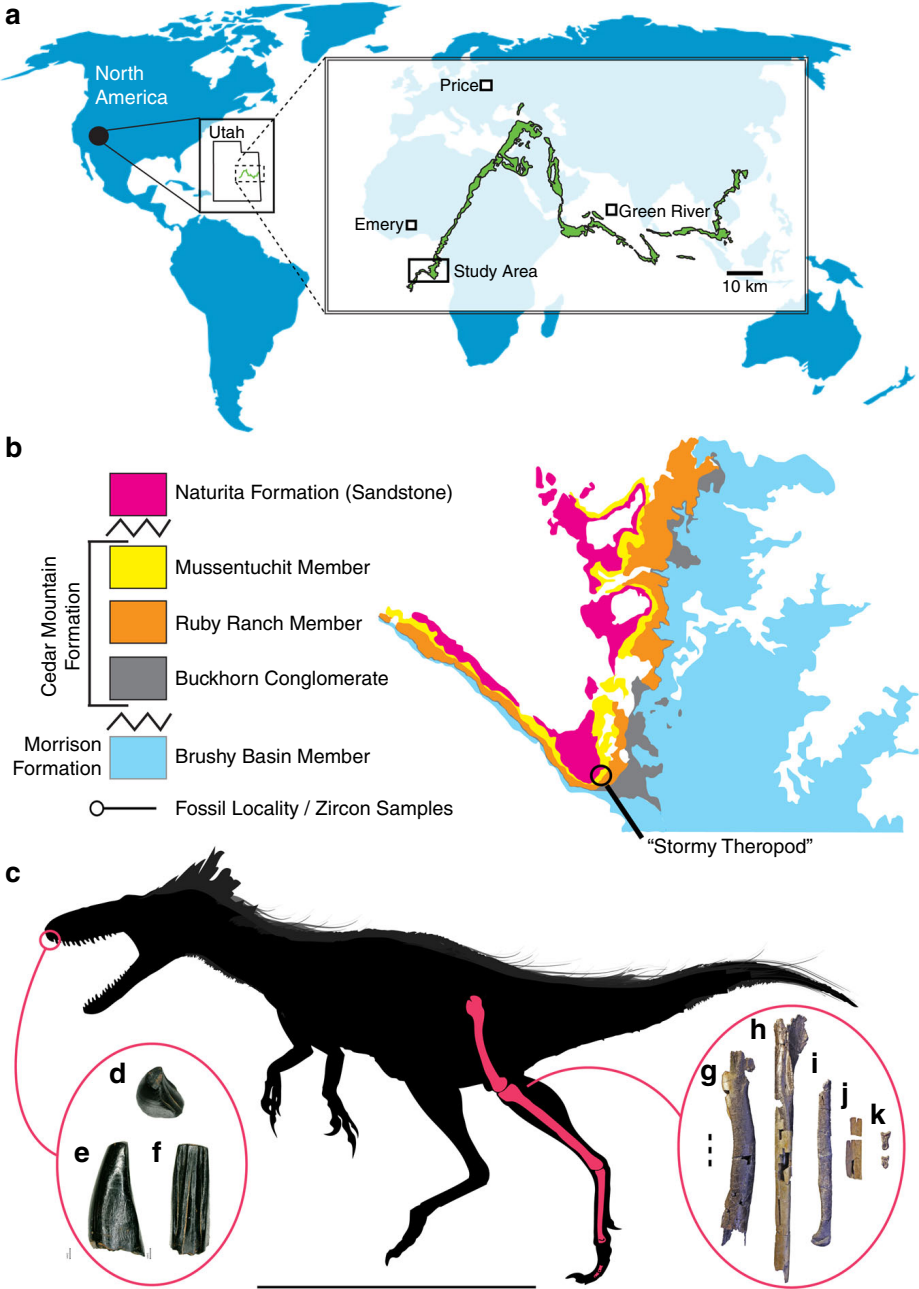
Location of holotype locality for *M*. *intrepidus* (NCSM 33392). (**a**) Global view showing extent of Cedar Mountain Formation outcrop in central Utah, (**b**) generalized stratigraphic section outcrop of the Cedar Mountain Formation in area of discovery, and (**c**) silhouette of *M*. *intrepidus* showing recovered elements. Isolated indet. tyrannosauroid premaxillary tooth (NCSM 33393) recovered from nearby strata in (**d**) occlusal, (**e**) mesiodistal, and (**f**) lingual views. Holotype specimen of *M*. *intrepidus* (NCSM 33392) composed of (**g**) femur, (**h**) tibia, (**i**) fourth metatarsal, (**j**) second metatarsal, and (**k**) pedal phalanges of the fourth digit. Scale bar (**c**) 1 m, (**g**–**k**) 5 mm. (**d**–**f**) Enlarged to show detail, not to scale

**Locality and horizon** NCSM 33393 and NCSM 33276 were recovered from Suicide Hill (NCPALEOUT11) and the Cliffs of Insanity Microsite (NCPALEOUT18), respectively, lower Mussentuchit Member, upper Cedar Mountain Formation, Emery County, Utah, USA (Fig. 1). NCPALEOUT11 and NCPALEOUT18 are located on land administered by the US BLM and State of Utah, respectively; access to exact locality information is restricted by federal and state statutes and is available to qualified researchers via the NCSM. Fossils at Suicide Hill are entombed in a laterally discontinuous lenticular crevasse splay. Microvertebrate fossils from the Cliffs of Insanity Microsite were recovered from volcanilithic-rich, fine-grained muddy sandstone, and muddy siltstone (Supplementary Methods 1, Supplementary Fig. 1).

**Fig. 2:**
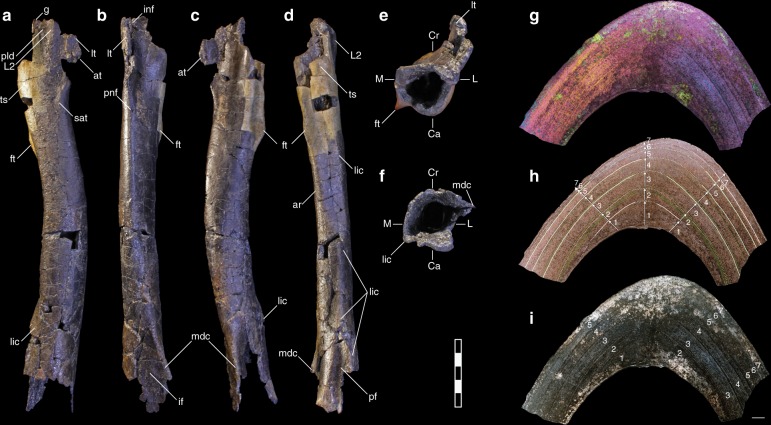
Right femur of *M*. *intrepidus* (NCSM 33392). (**a**) Lateral, (**b**) cranial, (**c**) medial, (**d**) caudal, (**e**) proximal, and (**f**) distal views. Partial mid-diaphyseal cross-section of the femur shown in (**g**) polarized light with lambda filter, (**h**) natural light with numbered arrows and tracings indicating seven growth cycles (see Supplementary Fig. 5), and (**i**) polarized light. *Abbreviations*: ar adductor ridge, at accessory trochanter, Ca caudal aspect, Cr cranial aspect, ft fourth trochanter, if intercondylar fossa, inf intertrochanteric nutrient foramen, L lateral aspect, L2 lobe on lesser trochanter (sensu^17^), lic linea intermuscularis caudalis. lt lesser trochanter, M medial aspect, mdc mesiodistal crest, pf popliteal fossa, pld lateral depression, proximal. pnf principle nutrient foramen, sat semicircular accessory tuberosity, ts trochanteric shelf. Scale bar (**a**–**e**) 5 cm; (**g**–**i**) 1 mm

**Diagnosis** (autapomorphy denoted with asterisk) tyrannosauroid premaxillary teeth exhibiting a salinon cross section; sharp, sinuous mesial, and distal carinae lacking serrations; and a pronounced lingual ridge; some teeth bearing a deep, obliquely oriented groove incising the lingual ridge* (Fig. 1; Supplementary Methods 2, Fig. 2).

**Description and comparisons** Two isolated premaxillary teeth bear lingually rotated mesial and distal carinae forming a salinon cross-section at mid-crown height, and a highly convex labial aspect as in tyrannosauroids generally^10–12^ (Fig. 1; Supplementary Figs. 2 and 3). In mesial/distal views carinae are sinuous, transitioning from lingually convex near the base to lingually concave approaching the occlusal surface (Supplementary Fig. 2d, f, h). Carinae terminate prior to reaching the root/crown juncture (Supplementary Fig. 2b). Mesial and distal aspects of the crown are depressed, yielding a weakly hourglass-shaped cross-section at the crown base (Fig. 1a; Supplementary Fig. 2). Crown height ranges from 6 mm (NCSM 33276) to 11.34 mm (NCSM 33393). Carinae lack serrations as in the Early Cretaceous tyrannosauroid *Xiongguanlong*^9^ (Supplementary Fig. 4) and an isolated tooth from the Cloverly Formation^12^. This feature has been interpreted as ontogenetically variable, yet serrations line the premaxillary teeth of *Tarbosaurus* beginning at 2-years of age^13^, suggesting it may be phylogenetically informative as suggested by Zanno and Makovicky^12^.

As in other tyrannosauroids, teeth exhibit a pronounced lingual ridge. The lingual ridge on the largest tooth is cleaved by a deep, obliquely oriented groove (Fig. 1d, g; Supplementary Fig. 2). However, the groove is not present on the smaller specimen (NCSM 33276) and may be an ontogenetic, or tooth position-dependent trait. To our knowledge, this feature has not been described on other tyrannosauroid premaxillary teeth and as such can currently be considered autapomorphic. However, given the dearth of premaxillary teeth in mid-Cretaceous taxa, damage to this region in teeth on other mid-Cretaceous tyrannosauroid teeth^12^, and difficulty in assessing premaxillary dentition in labial view in articulated skulls of tyrannosaurids, we consider it possible that the trait is more widespread.

*Moros intrepidus* gen. et sp. nov.

**Fig. 3:**
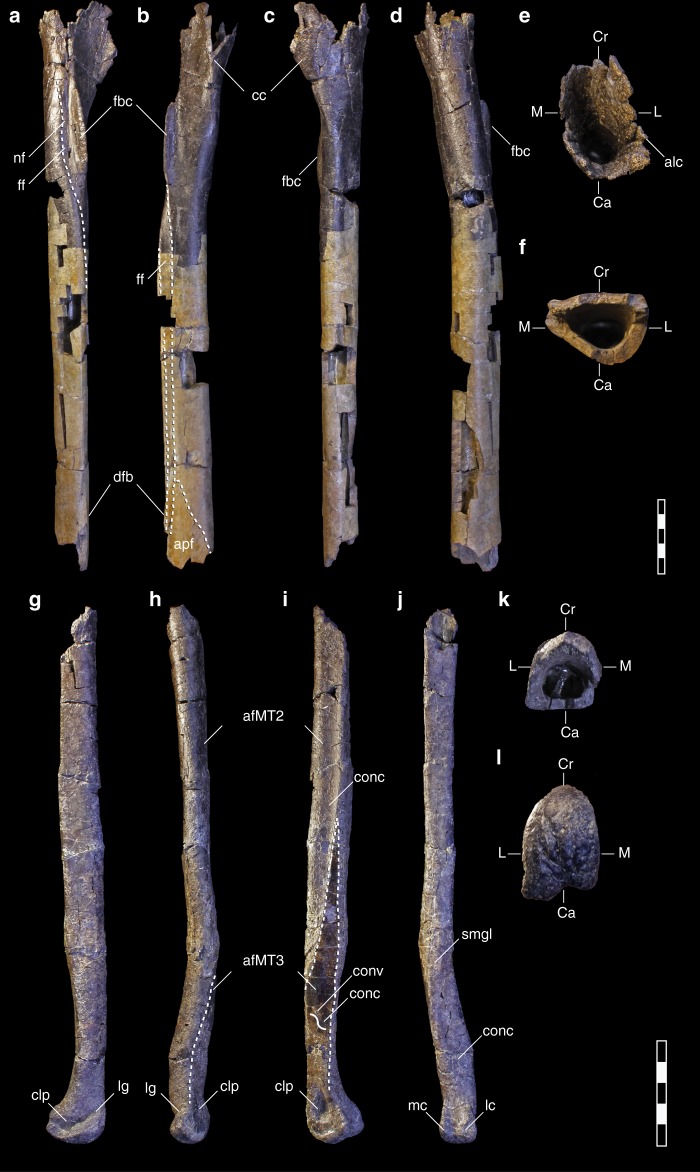
Right tibia (**a**–**f**) and right fourth metatarsal (**g**–**l**) of *M*. *intrepidus* (NCSM 33392). (**a**, **g**) lateral, (**b**, **h**) cranial, (**c**, **i**) medial, (**d**, **j**) caudal, (**e**, **k**) proximal, and (**f**, **l**) distal views. *Abbreviations*: afMT2 articular facet for second metatarsal, afMT3 articular facet for third metatarsal, alc accessory lateral condyle, Ca caudal aspect, cc cnemial crest, clp collateral ligament pit, Cr cranial aspect, conc concave surface of the articular facet for the third metatarsal, conv convex surface of the articular facet for the third metatarsal, dfb distal fibular buttress, fbc fibular crest, ff articular facet for the fibula, L lateral aspect, lc lateral condyle, lg lateral groove, M medial aspect, mc medial condyle, nf nutrient foramen, smgl scar for *M*. *gastroccnemius lateralis*. Scale bar 5 cm

**Fig. 4:**
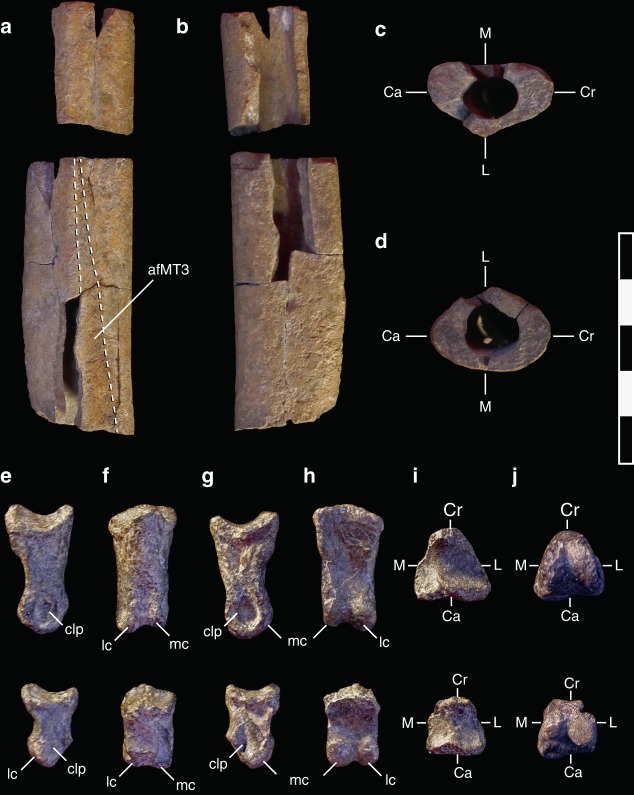
Right second metatarsal (**a–d**), right pedal phalanges IV–III (superior) and IV–IV (inferior) (**e-j**) of *M*. *intrepidus* (NCSM 33392). (**a**, **e**) lateral, (**b**, **g**) medial, (**c**, **i**) proximal, (**d**, **j**) distal, (**f**) dorsal, (**h**) ventral views. *Abbreviations*: afMT3 articular facet for third metatarsal, Ca caudal aspect, cc cnemial crest, clp collateral ligament pit, Cr cranial aspect, L lateral aspect, lc lateral condyle, M medial aspect, mc medial condyle. Scale bar 5 cm

**Holotype NCSM 33392**, an associated right hind limb of a subadult individual including portions of the femur, tibia, fourth, and second metatarsals and phalanges of the fourth digit (Figs. 2, 3, and 4).

**Etymology** *Moros*, (Greek) the embodiment of impending doom, in reference to the establishment of the Cretaceous tyrannosauroid lineage in NA, and *intrepidus*, (Latin) for intrepid, in reference to the hypothesized intracontinental dispersal of tyrannosaurs during this interval.

**Locality and horizon** NCSM 33392 was recovered from the lower Mussentuchit Member (6–7 m above the Ruby Ranch contact), upper Cedar Mountain Formation, Emery County, Utah, USA (“Stormy Theropod” NCPALEOUT05; Fig. 1). NCPALEOUT05 is located on land administered by the State of Utah; access to this information is restricted by state statute and is available to qualified researchers via the NCSM. Recovered skeletal remains and co-occurring detrital zircons were hosted within volcanilithic-rich, intercalated drab gray to light gray silty-mudstones and muddy siltstones. Site-specific facies analysis and architectural reconstruction indicates that sediments and fossil materials were emplaced along a coastal mudflat (with associated minor ephemeral channels and lakes) dated to no older than 96.4 Ma (average youngest maximum depositional age) via LA–ICP–MS analysis of recovered co-occurring detrital zircon grains (120 grains in total) (Supplementary Fig. 1, Supplementary Table 1, Methods I), which approximates the youngest ages reported for the Mussentuchit Mbr. in previous studies (96.7 and 97 Ma [recalibrated in 2007])^14,15^.

**Diagnosis** Small-bodied, gracile-limbed tyrannosauroid (Supplementary Tables 2 and 3) exhibiting a semicircular tuberosity on the craniomedial femoral shaft originating at the distalmost extent of the lesser trochanter* (Fig. 2a); sinuous articular facet on medial aspect of fourth metatarsal for contact with third metatarsal* (Fig. 3i); transversely compressed, subtriangular distal articular condyle of the fourth metatarsal in distal view (Fig. 3l); and distal articular surface of fourth metatarsal exhibiting hypertrophied craniolateral aspect*, confluent with a deeply incised, striated extensor groove that grades indistinctly into the lateral collateral ligament pit* (Fig. 3g). (Autapomorphies denoted with asterisk.)

### Description and comparisons

NCSM 33392 preserves a partial right hind limb including portions of the femur, tibia, second and fourth metatarsals, and phalanges of the fourth pedal digit (Figs. 2, 3 and 4; Supplementary Figs. 5–9). The proximal and distal aspects of the femur are poorly preserved—the femoral head, greater trochanter, and distal articular condyles have eroded—nonetheless, much of the femoral morphology can be discerned. *Moros* exhibits the base of an alariform, heavily striated lesser trochanter as in *Alioramus*^16^ and later diverging tyrannosaurids^17^. The base of the groove separating the lesser and greater trochanters and much of the intertrochanteric fossa is preserved (Fig. 2a), thus, although the relative height of the lesser and greater trochanters is indeterminate, it is clear they were separated by a deep, narrow cleft, as opposed to the broader condition of ornithomimosaurs (e.g., *Gallimimus* MPC-D 100/14 and *Garudimimus* MPC-D 100/13). A well-developed ridge rises from the lateral aspect of the lesser trochanter as in *Alioramus*^16^, *Gorgosaurus* (ROM 1247), and *Tyrannosaurus*^17^ and ornithomimosaurs (e.g., ROM 852, 797) (Fig. 2a). Caudal to this ridge and distal to the base of the lesser trochanter, a distinct, bulbous trochanteric crest extends from the caudomedial aspect of the shaft as in *Dryptosaurus*^18^, *Bistahieversor* (NMMNH P-25049), and *Gorgosaurus* (ROM 1247)(Fig. 2a,d). Together these features border a lateral depression as in tyrannosauroids generally (*Dilong*, *Guanlong*, *Alioramus*, *Dryptosaurus*^18^, *Gorgosaurus* [ROM 1247], *Bistahieversor* [NMMNH P-25049], *Albertosaurus*, *Tarbosaurus*, and *Tyrannosaurus*^16^), and to a lesser degree Late Cretaceous ornithomimids. A flange-like accessory trochanter is traceable extending from the distal-most lesser trochanter as in *Guanlong*, *Dilong*, and *Xiongguanlong*^9^ and an additional semicircular accessory tuberosity is present laterally, at the distal-most extent of the lesser trochanter (Fig. 2a). The medullary cavity is expansive and cortical bone relatively thin (Supplementary Methods 3).

Despite damage to the proximal aspect of the intertrochanteric fossa, the caudal margin of a relatively small nutrient foramen is preserved in this region (here termed an intertrochanteric nutrient foramina)(Fig. 2b; Supplementary Discussion 1.a) as observed in select eutyrannosaurs (e.g., *Tyrannosaurus*^17^ and *Alioramus*^16^ and some ornithomimids (e.g., *Gallimimu*s [MPC-D 100/14]; Bissekty and Bostobe Fms taxa^19,20^). A second, diminutive nutrient foramen is also preserved piercing the femoral shaft distal to the lesser trochanter (the principle nutrient foramen, sensu Madsen^21^, Supplementary Discussion 1.a) as in coelurosaurians generally. On *Moros*, the principle nutrient foramen is located medial to the long axis of the lesser trochanter as in other tyrannosauroids (Supplementary Fig. 7).

A subtriangular fourth trochanter rises from the caudomedial shaft (Fig. 2. c, d), extending proximally to overlap vertically with the trochanteric shelf as in *Bistahieversor* (NMMNH P-25049) and creating a “D”-shaped cross-section, bearing a flattened caudal aspect as in tyrannosaurids (e.g., *Gorgosaurus* ROM 1247; FMNH PR2211, *Albertosaurus sarcophagus* ROM 807) and *Ornithomimus* (ROM 797, 852). This flattening of the caudal shaft continues distally in *M*. *intrepidus*, being emarginated by two intramuscular lines, one extending distally from the base of the trochanteric crest (linea intermuscularis caudalis)^22^, and one from the fourth trochanter (i.e., adductor ridge, crista supracondylaris medialis)^22^ (Fig. 2d). In lateral profile, the midpoint of the fourth trochanter is slightly concave giving it a bimodal outline (Fig. 2c); the proximal-most aspect gradually fades into the proximal femur.

The shaft is bowed cranially, as is common in theropods generally, including tyrannosauroids (e.g., *Xiongguanlong*^9^, *Dryptosaurus*^18^, and *Tyrannosaurus*^17^). Erosion to the caudal aspect of the shaft precludes identification of muscle scars in this region; however, based on better preservation of the lateral aspect, if present, the scar for the *M. caudifemorali*s *longus* was located toward the medial margin.

In cross-section, the distal femur is highly skewed, with the longest diameter angled obliquely in a craniomedial/caudolateral orientation as in tyrannosauroids generally, and to a lesser degree ornithomimosaurs; we term this a lens-shaped cross-section of the distal femur (Fig. 2f). There is a pronounced, sharp mesiodistal crest rising from the medial condyle proximally (Fig. 2b–d); it is unknown if this crest bifurcated distally as in *Alioramus*, *Albertosaurus*, *Alectrosaurus*, or *Tarbosaurus*^16^. Caudal to the crest the medial shaft is clearly concave, yet lacks the posterior crest bounding an autapomorphic fossa in this region on *Dryptosaurus*^18^ and ornithomimosaurs (ROM 851). The linea intermuscularis caudalis morphs into a large bulbous tuberosity, forming the proximal rim of the flexor groove, and extending further distally as a robust caudolateral crest that was likely confluent with the crista tibiofibularis (Fig. 2d). Among ornithomimosaurs, this condition is similar to that observed on ROM 852, yet is not well-developed on other taxa. It is difficult to judge the depth of the intercondylar fossa (the proximal-most extent of the extensor groove) as only a portion is preserved and may be distorted; however, it appears more developed than *Dilong* and *Guanlong*, and we interpret it as similar to the condition in *Juratyrant*^23^, *Xiongguanlong*^9^, and *Gorgosaurus* (ROM 1247).

The tibia is slender, longer than the femur, and missing its proximal and distal articular condyles (Fig. 3a–d; Supplementary Fig. 10). The proximal portion bears the base of a well-developed, cranially extensive cnemial crest. The preserved portion suggests a mediolaterally narrow condylar surface. A convexity bordering the caudal margin of the cnemial fossa is suggestive of an accessory condyle extending off the lateral condyle (Fig. 3e), although it is unknown if this was hook-like as in *Xiongguanlong*^9^ and tyrannosaurids. An elongate fibular crest is subrectangular, in contrast to the distally sloping crest of ornithomimids^19^ (e.g., TMP 1994.012.1010). A nutrient foramen pierces the lateral aspect, as in theropods generally. The cross-section at midshaft is cranially flatted, with a convex caudal aspect (Fig. 3f), producing a strongly semicircular cross-section, a feature characterizing *Tyrannosaurus* (BMRP 2002.4.1). The fibular shaft would have been tightly appressed to the tibia as in *Bistahieversor* (NMMNH P-25049). A pronounced articular facet for the fibular shaft indicates that it shifted abruptly from a lateral to cranial position on the tibia and remained cranially oriented bracing the ascending process of the astragalus (Fig. 3b) as in *Tyrannosaurus* (BMRP 2002.4.1; FMNH PR 2081) and *Gorgosaurus* (ROM 1247; FMNH PR 2211). A ridge rises from the craniolateral aspect of the shaft at the point where the fibula and ascending process of the astragalus contact as in ornithomimids and other tyrannosauroids^16^, here termed the distal fibular buttress. A clearly delineated facet for the ascending process of the astragalus indicates that it was proximally extensive, oriented proximolaterally, and covered the entire face of the distal tibia at its base (Fig. 3b).

*Moros intrepidus* bears an unusually gracile arctometatarsalian pes, most closely resembling *Alectrosaurus* (AMNH 6554) in morphology and proportions (Fig. 3g–j; Supplementary Fig. 11). Preserved portions of the right metatarsus include a fragmentary, midshaft section of metatarsal two exhibiting surface erosion (Fig. 4a–d) and a nearly complete fourth metatarsal, missing the proximal-most articular aspect (Fig. 3g–l).

A mid-shaft section of the second metatarsal and associated fragments are preserved (Fig. 4a–d). The shaft fragment is subtriangular in cross-section, flattened medially, and bears a raised longitudinal ridge laterally (Fig. 4c, d). A portion of the facet for MTIII is preserved. It trends toward the flexor surface proximally (Fig. 4a), consistent with the facet for MTIII on MTIV. The plantar surface of the shaft is rugose as in other tyrannosauroids.

The fourth metatarsal is elongate (~270 mm) and slender, exhibiting an estimated proximodistal length to midshaft transverse width ratio of 1:21. This value is intermediate between tyrannosaurids (e.g., 1:10–16; ROM 1247, 807, BMRP 2002.4.1) and ornithomimids (1:22–32; ROM 851, 1790, 757). The proximalmost two-thirds of the preserved shaft is straight, “D” shaped in cross section (Fig. 3k), and highly symmetrical, bearing sharp caudomedial and caudolateral margins and a height to transverse width ratio of 1:1.4. The medial surface in this region is concave (Fig. 3i). These features characterize the fourth metatarsal shaft of tyrannosaurids (e.g., *Gorgosaurus* (ROM 1247), *Albertosaurus* (ROM 807), *Tyrannosaurus* (BMRP 2002.4.1), *Tarbosaurus*). Shaft compression (dorsoventral/transverse width) falls within the range observed for other Late Cretaceous tyrannosaurids (1:1–1.9; ROM 1247, 807, BMRP 2002.4.1) as opposed to the highly compressed metatarsals of Late Cretaceous ornithomimids (1:1.7–2.4; ROM 851, 1790, 757). Distally, the shaft flares laterally and there is a distinct scar for the *M. gastrocnemius lateralis* on the plantar surface at the point of deflection as in tyrannosaurids generally (e.g., *Gorgosaurus* (ROM 1247), *Albertosaurus* (ROM 807); Fig. 3j). Taphonomic distortion to the fourth metatarsal deemphasizes the degree of lateral deflection in *M*. *intrepidus*; however, it is still significantly more pronounced than observed in ornithomimids (Supplementary Fig. 8).

As in tyrannosaurids, the articular facet for MTIII is extensive (half the length of MTIV) and pinches out proximally, trending toward the palmar surface, suggesting MTIII would have been visible in palmar, yet not dorsal view. The facet itself is convex toward the extensor surface and concave toward the flexor surface giving it a sinuous profile in cross-section (Fig. 3i); this is in contrast to the uniformly convex facet observed in tyrannosaurids (e.g., *Gorgosaurus* [ROM 1247], *Albertosaurus* [ROM 807], *Tyrannosaurus* FMNH PR 2081) and likely represents incipient development of this feature. The plantar surface of the shaft just proximal to the distal condyles is concave and distinctly compressed craniocaudally as in *Gorgosaurus* (ROM 1247), and *Albertosaurus* (ROM 807) (Fig. 3j, Supplementary Fig. 8). Its medial aspect is rimmed by a ridge originating from the junction point of the proximal–most point of the distal condyles as in tyrannosaurids (e.g., *Gorgosaurus* [ROM 1247], *Albertosaurus* [ROM 807]; Supplementary Fig. 8). In lateral view, the distal aspect arcs caudally and the ventral margin of the distal condylar surface extends well beyond the shaft (Fig. 3g; Supplementary Fig. 9). Together, with the dorsoventral compression of the shaft just proximal to the distal articular surface, these features create a backswept distal aspect to the fourth metatarsal in tyrannosauroids (Supplementary Fig. 9). In contrast, the ventral aspect of the distal articular surface is relatively in-line with the shaft in ornithomimids (Supplementary Fig. 8).

The distal aspect of MTIV is transversely narrow (i.e., the maximum transverse width of the distal articular condyles is subequal in width to the distal shaft) and subtriangular with a dorsally tapering apex as in *Gorgosaurus* (ROM 1247), *Albertosaurus* (ROM 807), and *Coelurus* (YPM 2010) (Fig. 3l). In dorsal view, the distal condyle forms a bulbous, hypertrophied lateral margin that is delineated by a deep, oblique groove (Fig. 3g, h; Supplementary Fig. 9). This groove trends craniomedially to caudolaterally, and bears proximodistally oriented striations as in *Bistahieversor* (NMMNH P-25049), as well as a subadult *Tyrannosaurus* (BMRP 2002.4.1), and grades into a nearly indistinct lateral collateral ligament pit (Fig. 3g). A transversely compressed, subtriangular distal metatarsal bearing a groove and indistinct lateral collateral ligament pit, contrasts with the morphology observed in ornithomimosaurs, which exhibit a laterally flaring lateral articular condyle, gradual condyle to shaft transition on the dorsal surface, dorsally rounded distal condyle, and distinct (e.g., *Arkansaurus*;^24^ YPM 542; MPC-D 100/13; MPC-D 100/29), yet not always deep (e.g., *Gallimimus* [MPC-D 100/14, MPC-D 100/29]) lateral collateral ligament pit (Supplementary Fig. 9). In plantar view, lateral and medial distal condyles are distinguishable, the former being more than twice as wide mediolaterally as the latter (Fig. 3j). The medial collateral ligament pit is deep and relatively large, occupying half the craniocaudal width of the distal MTIV, and emmarginated by a raised rim (Fig. 3i).

Pedal phalanges IV-3 and IV-4 from the right foot are elongate as in tyrannosauroids generally (Fig. 4e–j), unlike the shortened pedal phalanges on digit IV of ornithomimids^25^. Phalanges are subtriangular in cross-section (i.e., transversely pinched on the extensor surface, relative to the plantar surface), bear weakly developed intercondylar ridges, and^26^ deep medial, yet shallow lateral collateral ligament pits.

## Discussion

We tested the broader evolutionary relationships of *Moros intrepidus* by incorporating it in a comprehensive phylogenetic matrix of coelurosaurs that encompasses tyrannosauroids^5^. Within this analysis, *M. intrepidus* is unequivocally recovered as a pantyrannosaurian, diverging after the Early Cretaceous Asian taxon *Dilong*, and prior to the major North American radiation of large-bodied Campano-Maastrichtian tyrannosauroids; however, the relationship between *M*. *intrepidus* and several other Late Jurassic–early Late Cretaceous species are unresolved (Supplementary Fig. 12).

**Fig. 5:**
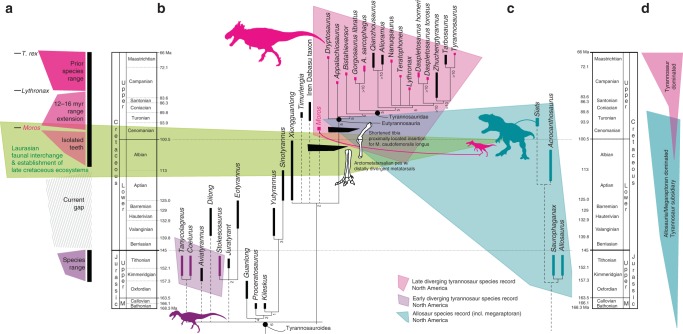
Phylogenetic relationships, chronostratigraphic, and paleoecological implications of *M*. *intrepidus*. **a** Graphic illustrating temporal range of North American tyrannosauroids including species-level range prior to the discovery of *M*. *intrepidus*, extension of current range, and hypothesized range based on isolated teeth^12^. The current gap in the North American tyrannosauroid record spans from the Tithonian to the Aptian. Faunal composition of Late Cretaceous ecosystems was established between the Albian and Turonian, as recognized by the stratigraphic appearance of major clades (see refs. ^7,12^ and references therein). **b** generalized phylogenetic relationships of Tyrannosauroidea, showing the appearance of select traits related to cursoriality in tyrannosaurs that are newly optimized as a result of the discovery of *M*. *intrepidus*. Tree topology follows this study using the modified dataset of Carr and colleagues^27^. *Coelurus* and *Tanycolagreus* are grafted as basal tyrannosauroids following Brusatte and colleagues^5^. **c** Stratigraphic distribution of Allosauria in North America (incl. Megaraptora but see ref. ^70^ for alternative hypotheses regarding this clade) documents overlap with *M*. *intrepidus* in early Late Cretaceous ecosystems leading to (**d**) refined calibration on the origin of late diverging tyrannosauroids and clade-level faunal turnover within apex predator roles throughout the Late Jurassic–Late Cretaceous of North America. Colored polygons are stylized call-outs and are not intended to reflect two-dimensional data. Temporal data corresponding to this figure are available in Supplementary Table 5

We used more targeted datasets that present alternative hypotheses of tyrannosauroid evolution^8,27^ to refine the interrelationships of *M. intrepidus*. Results of both analyses are consistent in positing *Moros* as an intermediate tyrannosauroid closely related to taxa inhabiting Asia during the mid-Cretaceous, such as *Xiongguanlong*, *Timurlengia*, and Turonian-Coniacian-aged^26^ materials from the Iren Dabasu Formation (e.g., *Alectrosaurus*, undescribed materials sensu^27,28^), and “relictual” taxa isolated to the island continent of Appalachia during the Late Cretaceous (i.e., *Appalachiosaurus* and/or *Dryptosaurus*) (Fig. 5). Nonetheless, topological differences exist related to character choice, coding discrepancies, and taxon sampling. Strict consensus of the Carr and colleagues dataset^27^ recovers all mid-Cretaceous tyrannosaur species in an unresolved polytomy at the base of Eutyrannosauria (Fig. 5b; Supplementary Fig. 13); whereas, the Loewen and colleagues dataset^8^ refines *M*. *intrepidus* within Eutyrannosauria, as sister-taxon to a clade containing *Appalachiosaurus* plus all later diverging tyrannosauroids (Supplementary Fig. 14) (a single MPT from the Carr et al. dataset^27^ recovered this topology also; Supplementary Fig. 13d). Taken together, these data suggest that *Moros* diverged penecontemporaneously with the establishment of the North American tyrannosaur lineage, providing a long-awaited evolutionary precursor for taxa that would radiate into top predator niches across both western and eastern NA prior to the extended isolation of Laramidia and Appalachia in the middle Cenomanian^29^.

Reconstructed growth dynamics of large-bodied eutyrannosaurians from the Campano-Maastrictian of NA demonstrate that gigantism in the most massive species (i.e., *T*. *rex*) was achieved largely through acceleration of growth rates, with extension of the active growth period having only minor effects^3^. However, only a single pre-Campanian tyrannosauroid species has been histologically sampled (*Guanlong*^11^) leaving growth rates throughout 90% of tyrannosauroid evolution (~80 million years) unexplored. This is despite the recognition that body size (using femoral length as a proxy) jumps six-fold between the earliest tyrannosaurs (e.g., *Dilong*^30^) and their large-bodied early Campanian descendants, and is inextricably linked to their ascent into top predator roles.

To assess the terminal age, relative growth rate, and ontogenetic stage of NCSM 33393, we processed histological ground sections from mid-diaphyseal fragments of the tibia and femur. Microanatomy of these elements is typical for theropod dinosaurs in bearing an expansive medulla and compact cortex^31^. Bone tissue of the tibia and femur exhibit highly degraded microstructure due to extensive microbial invasion and bioerosion of the outermost and deep cortical regions. Structures reminiscent of Wedl tunnels, resulting from the invasion of the vascular network by fungal hyphae that spread into the bone matrix, are visible throughout the cortex^32,33^ (Supplementary Fig. 6a). Of the two sections examined, the femoral histology is better preserved due to lack of surface exposure and was used to determine growth stage.

The periosteal cortex is zonal, consisting primarily of parallel-fibered bone matrix (presenting bulk anisotropy in polarized light; Supplementary Fig. 6b–d) dominated by longitudinal simple vascular canals and primary osteons (Supplementary Fig. 6b–d) and delineated by single, doublet, triplet, and even quadruplet Lines of Arrested Growth (LAGs; Supplementary Fig. 6a; Fig. 2g–i). We counted up to 16 LAGs throughout the cortex, identifying six–seven growth cycles based on the main extinction patterns (Fig. 2g–I; Supplementary Fig. 5b). Doublet to quadruplet LAGs are commonly observed in non-avian theropod dinosaurs^31,34^ and we interpret these as single growth events^35^. The predominance of a parallel-fibered bone matrix dominated by longitudinal vascular canals suggests a slow to moderate growth rate for this animal^36^, in contrast with the highly vascularized laminar to plexiform fibrolamellar bone tissues and rapid growth rates observed in large-bodied tyrannosaurids^3^.

The bone at the periosteal surface is degraded, therefore the presence of an external fundamental system and changes in vascularization cannot confidently be assessed; however, tissue thickness between growth cycles gradually diminishes towards the bone periphery (Fig. 2g–i, Supplementary Fig. 5b), implying a slowing of growth. Cortical regions preserving histological integrity are comprised exclusively of primary bone tissue; no secondary osteons are observed. However, secondary remodeling cannot be ruled out within the deep cortex as various portions are obliterated by bioerosion. If present originally, remodeling must have been limited. These indicators, together with the presence of a single, thin layer of lamellar endosteal bone tissue lining the endosteal margin (Supplementary Fig. 6e and f), attest that the expansion of the medullary cavity had ceased and growth was slowing, and confirm that *M*. *intrepidus* would have remained a diminutive species at skeletal maturity.

We conclude that NCSM 33392 derives from a skeletally immature individual nearing adult size, exhibiting a slow to moderate growth rate, and a terminal age of at least 6–7 years. This pattern of growth, including relative mean rate (a function of size and years to somatic maturity) and actual age at somatic maturity, compares well with *Guanlong*^11^, a significantly more primitive tyrannosauroid from the Late Jurassic of Asia, which reached senescence at 7 years of age and was similarly sized (femur length (FL) = 342 mm). By contrast, large-bodied, Campano-Maastrichtian tyrannosaurines (e.g., *Gorgosaurus*) were already triple the mass at similar ages and approaching an exponential growth phase^3^. Although limited, these data raise the possibility that growth rates throughout much of tyrannosauroid evolution were relatively conserved. Of particular interest is whether an independent trend of increasing mass observed within pre-Campanian taxa from the Cretaceous of Asia (e.g., *Yutyrannus*^37^, *Sinotyrannus*^38^ was also achieved via rate acceleration or if dramatically heightened growth rates were one of the last evolutionary innovations of tyrannosaurines.

Although fragmentary, *M*. *intrepidus* is key for refining the timing of, and morphological trends leading up to, the successful radiation of Campanian tyrannosauroids. Comparative quantifications for a subadult *M*. *intrepidus* result in an estimated FL and tibia length (TL) of 355 and 440 mm, respectively; a skull length of 300–400 mm; an interpolated mass of 78 kg (range 53–85 kg) (Supplementary Table 3); and an approximate limb length of 1.2 m (Supplementary Table 2). Thus, *M*. *intrepidus* ranks as one of the smallest Cretaceous tyrannosauroids yet described. This finding, together with evidence for diminutive tyrannosauroids in the Albian (ca. 108 Ma^12^) indicates that: (1) North American tyrannosauroids were restricted to small sizes for a protracted period of ~15 million years of the mid-Cretaceous and (2) at some point during or after the Turonian, tyrannosauroids embarked on a trend of rapid body size increases, more than doubling in femoral length during a window of no more than 16 million years between the Turonian and Campanian (Supplementary Table 3).

Aside from its diminutive stature, *M*. *intrepidus* is notable for possessing a near complete, remarkably gracile hind limb and highly compressed pes that elucidates the timing of biomechanical changes associated with enhanced cursoriality in tyrannosauroids, including elongation of the distal limb, increasingly slender limb segments, and more proximally located muscle insertions^39^. Proportions of the zeugopod to stylopod of NCMS 33393 are most similar to those of North American ornithomimids (e.g., *Struthiomimus* AMNH 5339^40^, *Ornithomimus* ROM 852, 797), *Alectrosaurus*^40^ from the Iren Dabasu Formation and immature growth stages of select large-bodied Campanian species (e.g., *D*. *horneri* MOR 1130; *G*. *libratus* TCMI 2001.89.1, and *Raptorex*^41^) (Supplementary Fig. 10; Supplementary Table 3). Pes slenderness of *M*. *intrepidus* is even more extreme, falling well outside the range of juvenile tyrannosauroid specimens and clustering with ornithomimids (Supplementary Fig. 11; Supplementary Table 4). *Moros* also possesses a combination of the youngest evidence of proximally located fourth trochanter (insertion point of the primary limb retractor, *M. caudofemoralis longus*^22^ and elongate tibia; together with the oldest record of an arctometatarsalian pes bearing a wedge-shaped third metatarsal, distally diverging articular surfaces, and rugose, oval scar for the *M. gastrocnemius lateralis* (Fig. 5b). This combination of traits refine the origin of the arctometatarsalian pes in tyrannosaurs as having occurred no later than Cenomanian (Fig. 5b), and suggest small-bodied taxa inhabiting Cenomanian–Santonian ecosystems were among the last to maintain adaptations for extreme cursoriality, prior to their ascent into top predator roles.

Although pulses of mass extinction are studied intensely for their swift and dramatic impact on ecosystem composition and stability, other, more gradual transitions are recognized to have fundamentally restructured trophic networks during Earth’s history. Key among these is the mid-Cretaceous, a dynamic interval that records a substantial faunal and floral reorganization of food-webs, including top predator guilds. Ecosystem reassembly during the mid-Cretaceous is linked to the dispersal of terrestrial vertebrates across northern landmasses via subaerial exposure of Beringia and coincident extirpation of endemic terrestrial vertebrate lineages^7,12,14^—phenomena that together, rank as the seminal event leading up to the establishment of the iconic Late Cretaceous biotas of NA.

Transcontinental interchange during the mid-Cretaceous is well-established, having now been recorded in phylogenetically contextualized biogeographic data from an extensive array of lineages including mammals, squamates, turtles, ornithischian, and coelurosaurian dinosaurs (see Zanno and Makovicky^12^ and references therein); however, the tempo and stepwise pattern of faunal restructuring has yet to be detangled, and for some key clades (e.g.,Tyrannosauroidea), definitive evidence of interchange has been notably lacking. Significant progress was recently made in refining the timing of apex predatory guild restructuring in NA through documentation that allosaurians maintained their role as top predators from at least the Late Jurassic to the early Late Cretaceous^7^. The discovery of *Siats* in Upper Cretaceous beds of the Mussentuchit Member, Cedar Mountain Formation (Fig. 5c), alongside the discovery of isolated, diminutive tyrannosauroid teeth from both the Mussentuchit Member and the Albian-aged Cloverly Formation (Fig. 5a) bearing synapomorphic features with mid-Cretaceous Asian taxa^12^, provided intriguing, yet inconclusive, evidence that tyrannosauroids from this interval remained subsidiary predators. *Moros intrepidus* solidifies this hypothesis, providing the first unequivocal skeletal evidence that (1) tyrannosauroids were lower-level predators through at least the Turonian of NA (Fig. 5d); and (2) that the origin of tyrannosauroids in the Cretaceous of NA is most parsimoniously interpreted as resulting from faunal exchange with Asia prior to the Cenomanian^12^ (Fig. 5a).

Top predators play a key role in shaping ecosystems^42–44^ and can act as buffering agents during intervals of ecological disruption, increasing resilience against environmental change^45^, and biological invasion^46^. Current evidence suggests that small-bodied, highly cursorial tyrannosaurs with advanced sensory toolkits invaded the North American continent no later than the Albian, yet were unable to ascend to top predator roles until a combination of physiogeographic, climatic, and paleoenvironmental changes, such as transgression of the Western Interior Seaway and shrinking species ranges^8,47,48^, a global temperature maximum in the Turonian^49^, and shifts in precipitation and climate^50^ likely combined to wipe out allosaurians as a stabilizing effect on Late Cretaceous ecosystems.

## Methods

### Body mass and proportions

We used the MASSTIMATE package^51^ (version 1.3) in R version 3.3.3^52^ to compute the mass of *M*. *intrepidus* (NCSM 33392**)** based on five equations utilized for bipedal nonavian dinosaurs^51,53–55^. We used the default correction factor (2) and the “phylocor” estimation equation for cQE, which uses a phylogenetically corrected equation from Campione and colleagues^51^.

Interpolation of tibial and femoral lengths for *M*. *intrepidus* is complicated by the absence of both proximal and distal articular surfaces, yielding two variables to compound potential error. Our estimates are derived by necessity from the ratio of FL to the length derived from the distalmost origin of the lesser trochanter to the proximal-most origin of the mesiodistal crest, and from tibial length to the distance from the proximal-most fibular crest to the point of distal expansion of the tibia. These are uncommonly reported data; therefore, we used only direct measurement of skeletally mature and immature tyrannosauroid specimens for our estimations (BMRP 2002.4.1 and MOR 1125). We then compared two different femoral ratios independently derived from our estimated and direct measurements to the observed range reported for theropods and tyrannosauroids to check for congruence. The lesser trochanter occupies 21–27% the length of the femur in tetanurans^23^. Our interpolated FL in *M*. *intrepidus* yields a lesser trochanter that occupies 23% of the femur, concordant with this range. Moreover, the distance between the proximal-most femur and distal-most aspect of the fourth trochanter as estimated for *M*. *intrepidus* is 34%, congruent with the proportions reported for early diverging, Late Cretaceous tyrannosauroids (35%, incl. *Dryptosaurus* and *Appalachiosaurus*), and contrasting with the more elongate fourth trochanter of tyrannosaurids^18^ (40%).

We used the mesiodistal basal crown width of the third premaxillary tooth of *Xiongguanlong baimoensis* (FRDC-GS JB16-2-1; Supplementary Fig. 4) to extrapolate the skull length of the tyrannosauroid bearing the largest premaxillary tooth in our sample (NCSM 33393). Basal crown width of premaxillary teeth in FRDC-GS JB16-2-1 are ~6.6 mm, which when compared to basal skull length of 504 mm^9^ yields a skull length to premaxillary tooth width ratio of 76.36:1. Thus, if similar in proportions, NCSM 33393 derives from a skull ~309 mm in length. This value compares well (within 12%) to a skull length for NCMS 33392 (352 mm) interpolated from the skull length to femoral length (510 mm) ratio in *Xiongguanlong*^9^ (0.988:1). Thus, the skull length of *M*. *intrepidus* likely lies in the 300–400 mm range.

### Paleohistology

Prior to sampling, specimens were cast and digitized via photogrammetry. Bone fragments bearing no diagnostic features were sampled mid-diaphysis—the region of bearing the thickest cortical compacta, thereby preserving the most complete growth record^56^—following original cracks in the bones. Petrographic techniques common to bone paleohistology were employed^57^. Sampled bone fragments were embedded in a clear epoxy resin (EPO-TEK 301), cut along the transversal plane with a Buehler IsoMet 1000 Precision Saw, and polished on one side with a Buehler MetaServ 250 Grinder Polisher using a series of abrasive paper disks with increasing grit sizes (400–1200). The polished blocks were mounted on frosted glass slides with epoxy, and ground to desired thickness (≈100–80 µm). Thin-sections were observed with a Nikon Eclipse Ci POL microscope equipped with a polarizer and a lambda filter, and imaged with a Nikon DS-Fi2 digital camera.

### Phylogenetic Protocol

We analyzed the evolutionary relationships of *Moros intrepidus* by inclusion within three phylogenetic data matrices^5,8,27^. Data coding, character tracing and tree manipulation/visualization for all phylogenetic analyses were carried out using Mesquite ver. 3.51^58^. Phylogenetic analyses were executed in the program TNT^59^. We assessed results from all analyses using strict and reduced consensus methods and Bremer support values^60^. Ambiguous nodes were uniformly collapsed following Rule 1 of Coddington and Scharff^61^. We followed original authors in the designation of additive characters and outgroup taxa. Maximum agreement subtrees^62^ were calculated in TNT and used to identify labile taxa and common topology among all MPTs in each analysis. Importantly, we did not include the Mussentuchit premaxillary teeth in the *M*. *intrepidus* OTU. We find the accessory trochanter morphology of *M*. *intrepidus* consistent with that of *Xiongguanlong* and have coded it accordingly. An extensor groove in *M*. *intrepidus* is indeterminate due to preservation; however, a weakly concave extensor fossa is visible. The morphology of the extensor groove is addressed differently in various matrices. When the morphology of the extensor groove and extensor fossa are considered as one trait^5,27^ we coded this trait as present yet, unknown in degree of development. We include updated character scorings for *Xiongguanlong* in all three analyses based on personal observations and research photographs of the specimens; changes are described below. See Supplementary Discussion 2 for data files.

### Brusatte et al. 2016

The analysis includes 154 taxa and 852 characters. We interpret the mesiodistal crest of *Xiongguanlong* as bifurcating (character 716, state 1) and note that serrations are not present on the premaxillary dentition^9,12^ (character 783, state 1). We further changed all tibial characters to uncertain (?) following the suggestion by Li and colleagues^9^ that the tibia is questionably referable to *Xiongguanlong*. We also removed character 472 as it is a duplicate of character 226. We replicated the analytical protocol by conducting a new technology search, with sectorial, ratchet, drift, and tree fuse under default parameters, and in excluding *Kinnareemimus*, *Epidendrosaurus*, *Pyroraptor*, *Hesperonychus*, and *Limenavis* prior to generating the strict consensus tree^5^ (Supplementary Fig. 12). A most parsimonious tree (MPT) was found in five replicates. We followed this with a traditional search on all trees stored in RAM (8 MPTS).

### Carr et al. 2017

The analysis includes 29 ingroup taxa and 386 characters. We conducted heuristic searches on Wagner trees using TBR branch-swapping with 10,000 random addition sequences of 10 random seeds holding 10 trees per replicate, continuing subsequent TBR swapping on all stored minimum length trees (36 MPTs, TL 784). From MPTS we generated strict consensus (Supplementary Fig. 13) and agreement subtrees. *Xiongguanlong* lacks a cornual process on the lacrimal (sensu Li and colleagues^9^) thus we changed character 66 to state 0, and accordingly changed characters 68, 69, and 71, to n/a (?) in that these describe the morphology of the cornual process (although the corunal process was coded as absent for character 67 in the original character scorings of Carr and colleagues^27^). We also changed 359 to state 1 (femoral circular scar, abuts medial edge) but note that some taxa are coded as having a state “2” for this character within the dataset of Carr and colleagues^27^, yet a state “2”) is not described on the character list. Finally, we coded a bifurcating mediodistal crest as present (char 367).

### Loewen et al. 2013

The analysis includes 55 taxa and 501 characters. We follow Carr and colleagues^27^ in excluding the juvenile specimen *Raptorex kriegsteini* and *Bagaraatan* as the latter has recently been determined to be a chimera, but did not combine *Alioramus altai* as with *A*. *remotus*, and relabeled the “Two Medicine Fm. tyrannosaurid” taxon as *D*. *horneri*. Here again, we changed character scorings for *Xiongguanlong* by changing all tibial characters to uncertain, scored premaxillary dentition as lacking serrations (character 294, state 1), and changed character 452, to state 1, interpreting the position of the femoral scar as caudomedial. We conducted heuristic searches on Wagner trees using TBR branch-swapping with 10,000 random addition sequences of 10 random seeds holding 10 trees per replicate, continuing subsequent TBR swapping on all stored minimum length trees (2 MPTs, TL 1727). We tested node strength by calculating Bremmer values from a suboptimal search of 1000 random addition sequences of 10 random seeds holding 10 trees per replicate, continuing subsequent TBR swapping on all stored minimum length trees until 99,999 trees were captured. Strict consensus tree is shown in Supplementary Fig. 14.

### Sedimentology

Sedimentologic analysis of the Mussentuchit Member Formation was conducted between 2014 and 2017. Detailed facies and architectural element analysis was performed following the conceptual framework established by Miall^63^ and modified by subsequent researchers^64,65^. For consistency, a uniform set of facies codes was used to describe both the outcrop sections and the cores. In outcrop, detailed measured sections were constructed at the decimeter scale in each of the three study areas using a Jacob’s Staff, Brunton Compass, and GPS. The following types of field data were collected for each area and section: (1) lithology; (2) the nature of the upper and lower bounding surfaces; (3) external unit geometry and lateral extent (i.e., architectural elements sensu^63^); (4) scale and thickness of units; and (5) sedimentary and biogenic structures. Particular emphasis was placed on understanding and correlating important vertebrate fossil localities in each study area. Many horizons were walked out in order to define the horizontal and lateral continuity of beds and facies.

### Zircon analysis

Sample preparation was conducted at the Central Analytical Facility, Stellenbosch University, Stellenbosch, South Africa. Samples were crushed and milled in a tungsten carbide disc mill and then sieved using both 250 and 500 μm meshes, washed and decanted numerous times to remove the clay-sized fraction, and lights were separated using Tetrabromoethane (TBE) with a density of 2.96. Mineral separates were then washed, dried, sorted via a Frantz magnetic separator at progressively higher magnetic currents, and non-magnetic heavy mineral separates were then handpicking as randomly as possible from the greater population within a defined field of view. For each sample, ±150 grains were mounted, polished, and documented (via a Zeiss MERLIN Field Emission Gun Scanning Electron Microscope) to access microstructures, cracks, inclusions, and other complexities.

Laser ablation U-Pb data were collected at the Central Analytical Facilities, Stellenbosch University, using a 193 nm wavelength ASI Resolution laser ablation system coupled to a Thermo Scientific Element 2 single collector magnetic sector field inductively coupled plasma mass spectrometer (SC-SF-ICP-MS). Over the duration of ablation, groups of 10–13 zircon grains were analyzed, followed by at least two analyses each of a primary (GJ-1, 609 Ma^66^ and secondary in-house zircon standard (Pleasovice 337.13 ± 0.37^67^). Data reduction was conducted via Iolite. If grains exhibited a greater discordance of 15%^68,69^, those grains were omitted from the populations and the study as a whole. All standard analyses were within 2% of the expected ages, and most were within 1% of the expected age. Youngest maximum depositional age was calculated by combining the results for YSG, YDZ, YC1s [+3], weight average, YC2s [+3], and TuffZirc [+6]) (Supplementary Fig. 1) (for detailed methods see ref. ^68^)

### Nomenclatural acts

This published work and the nomenclatural acts it contains have been registered in ZooBank, the proposed online registration system for the International Code of Zoological Nomenclature (ICZN). The ZooBank LSIDs (Life Science Identifiers) can be resolved and the associated information viewed through any standard web browser by appending the LSID to the prefix ‘http://zoobank.org/

The LSID for this publication is urn:lsid:zoobank.org:pub:00135AFF-8A92-4358-8E7A-9B3569C0098B

### Reporting summary

Further information on experimental design is available in the Nature Research Reporting Summary linked to this article.

## Supplementary information

Supplementary Information

Reporting Summary

## Data Availability

The authors declare that all measurements and phylogenetic data supporting the findings of this study are included in this published article (and its Supplementary Information files) and that all paleontological specimens including paleohistological sections generated during the current study are available for access by qualified researchers by request at the North Carolina Museum of Natural Sciences.
