# Supplementary material for: Diminutive fleet-footed tyrannosauroid narrows the 70-million-year gap in the North American fossil record
Source: Commun Biol. 2019 Feb 21;2:64. doi: 10.1038/s42003-019-0308-7 (PMC6385174; doi:10.1038/s42003-019-0308-7)
Supplement: Supplementary file 1 — Supplementary Information [file 42003_2019_308_MOESM1_ESM.docx]

# SUPPLEMENTARY INFORMATION FOR:

**Diminutive, fleet-footed tyrannosauroid narrows the 70-million-year gap in the fossil record of North American “tyrants”**

CONTENTS

**Supplementary Methods 1**: Geological context and age dating

Supplementary Figure 1: Temporal placement of *M*. *intrepidus*.

Supplementary Table 1: Youngest single grain (YSG) results for all samples including

isotope and concentration data.

**Supplementary Methods 2:** Specimen measurements and additional images of tyrannosauroid teeth

Supplementary Figure 2: Additional images of NCSM 33393 (isolated tyrannosauroid

premaxillary tooth).

Supplementary Figure 3: Select measurements of NCSM 33393 (isolated

tyrannosauroid premaxillary tooth).

Supplementary Figure 4: Measurement of mesiodistal basal crown width on third

premaxillary tooth of *Xiongguanlong*.

Supplementary Table 2: Select measurements of subadult specimen of *M*. *intrepidus*

(NCSM 33392).

Supplementary Table 3: Mass estimates of the subadult specimen of *M*. *intrepidus*

(NCSM 33392).

**Supplementary Methods 3:** Additional figures illustrating the paleohistology of NCSM 33392

Supplementary Figure 5: Location of paleohistological section of femur and details on

growth markers (*M*. *intrepidus,* NCSM 33392).

Supplementary Figure 6: Bone microstructure of *M*. *intrepidus* (NCSM 33392).

**Supplementary Discussion 1:** Additional discussion on the anatomy and taxonomic

identity of *M*. *intrepidus*

1.a Discussion of the femoral nutrient foramina in theropods

Supplementary Figure 7: Position of nutrient foramina on theropod femora

2.b Distinguishing *M*. *intrepidus* from ornithomimosaurs and oviraptorosaurs

Supplementary Figure 8: comparative anatomy of the MTIV of tyrannosauroids

and ornithomimosaurs in plantar view

Supplementary Figure 9: comparative anatomy of the MTIV of tyrannosauroids and

ornithomimosaurs in lateral view

Supplementary Figure 10: OLS linear regression of proximodistal femoral length (x-axis)

against proximodistal femoral tibial length (y-axis) in tyrannosauroids, ornithomimosaurs, and *M*. *intrepidus* (NCSM 33392).

Supplementary Figure 11: Biplot of metatarsal proportions in tyrannosaurids,

ornithomimids, and *M*. *intrepidus* (NCSM 33392).

Supplementary Table 3: Femur and tibia measurements of *M*. *intrepidus* (NCSM 33392),

other tyrannosauroids, and ornithomimids.

Supplementary Table 4: Metatarsal measurements of *M*. *intrepidus* (NCSM 33392),

other tyrannosauroids, and ornithomimids.

**Supplementary Discussion 2:** Phylogenetic and Temporal Data

Supplementary Table 5. Temporal data for Figure 5.

Supplementary Figure 12: Strict consensus tree resulting from the inclusion of *M*. *intrepidus*(NCSM 33392) in the updated matrix of Brusatte et al. (2017).

Supplementary Figure 13: Hypothesized relationships of Tyrannosauroidea

including *M*. *intrepidus* (NCSM 33392) in the updated matrix of Carr et al. (2017).

Supplementary Figure 14: Strict consensus tree resulting from the inclusion of *M*.

*Intrepidus* (NCSM 33392) in the updated matrix of Loewen et al. (2013).

Modified matrix file Brusatte et al., 2017 with *M*. *intrepidus*.

Modified matrix file Carr et al., 2017 with *M*. *intrepidus*.

Modified matrix file Loewen et al. 2013 with *M*. *intrepidus*.

**Supplementary References**

# Supplementary Methods 1: Geological context and age dating

*Moros intrepidus* (NCSM 33392) derives from the lower portions of the Mussentuchit Member (6-7 meters above the Ruby Ranch contact), upper Cedar Mountain Formation. The Cedar Mountain Formation was deposited within the San Rafael Swell (distal backbulge to eventual forebulge of the Sevier fold-thrust belt), a portion of the Western Interior Basin (a long-lived retroarc foreland basin)1-8. Transition to the forebulge phase during basin development is preserved within a suit of sediments originally defined by Stokes9 as the Cedar Mountain Formation, with recent subdivisions into the lowermost 1) Buckhorn Conglomerate; 2) Yellow Cat; 3) Poison Strip; 4) Ruby Ranch; 5) Short Canyon; and lastly 6) Mussentuchit Member5, 10-12.

Recovered skeletal remains NCSM 33392 and co-occurring detrital zircons were recovered from volcanilithic-rich, intercalated drab grey to light grey silty-mudstones to muddy- siltstones (lithic wacky/mud rocks) of the lower Mussentuchit, which, in general, is characterized additionally by muddy siltstones, with sporadic laterally discontinuous lenticular crevasse splays, sheet floods and distal, ribbon-like deltaic-channel sandstones. These lower mud-rich sediments are in contrast to the overlying upper Mussentuchit sand-silt rich units. Sediment and volcanic detritus was supplied by fairly regular periods of hyperpycnal channel flow of bedload-rich rivers draining from adjacent high relief tectonically active terrain(s) to the west 13-14. Sediment would splay across the mud-flat, with wave and tidal alterations thereafter14. This is in agreement with Bhattacharya and MacEachern13 suggesting similar input into the adjacent Greenhorn Seaway and the eventual construction of the Last Chance Delta around the mid-Turonian. High rates of sediment output, coupled with sea level rise at or between 101-95 Ma indicates that the lower portions of the Mussentuchit Member likely represent the predominance of wave to tidally influenced coastal aggradating sedimentation during the onset and middle phases of the Greenhorn Transgression with periodic mass-shedding of detritus from the westerly lying

arc13,16-18.


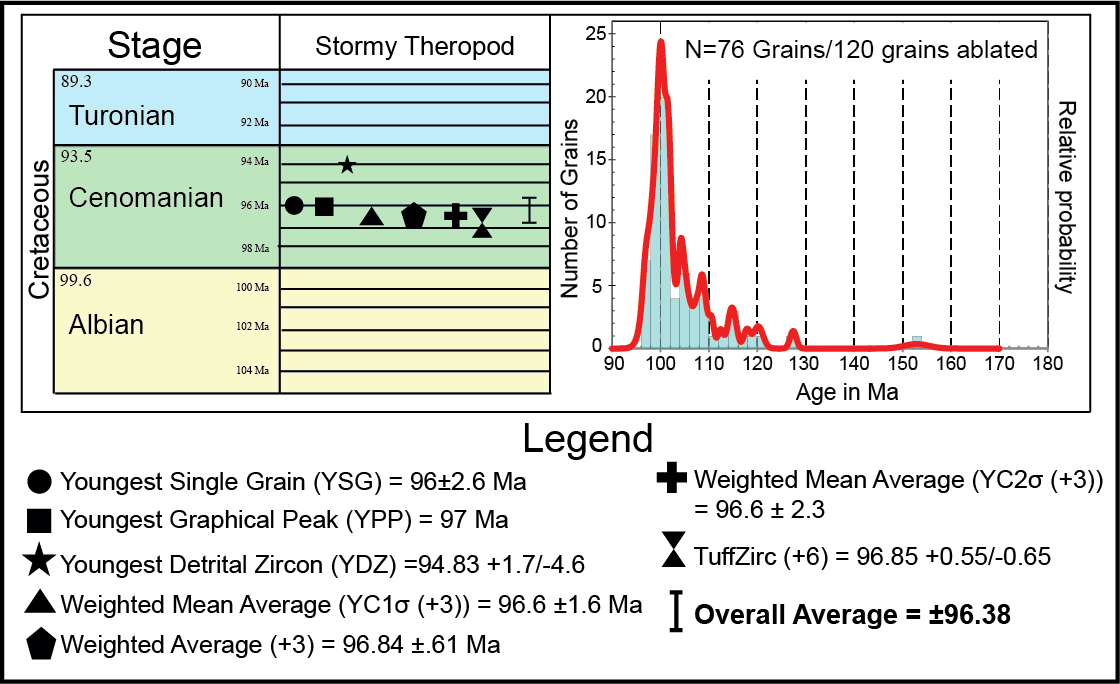


**Supplementary Figure 1: Temporal placement of *M*. *intrepidus*.** Analysis was conducted at the Central Analytical Facility (CAF) of Stellenbosch University, South Africa. During each analytical session the zircon reference materials GJ-1 (Jackson et al., 2004) and Plešovice (Sláma et al., 2008) were ablated between groups of 10-13 unknowns. Zircon GJ-1 was used as a matrix- matched primary reference material to correct for mass discrimination on measured isotope ratios in unknown samples and simultaneous correction for instrumental drift (based on Horstwood et al., 2016), and secondary standard Plešovice was utilized to validate the results and assess the quality of the data for each analytical session. Plešovice: Wtd avg 206Pb/238U age = 341.4 ± 0.5 Ma (95% conf, MSWD = 1.2; n = 82); 91500: Wtd avg 206Pb/238U age = 1060 ± 2 (95% conf,

MSWD = 1.0; n = 44). Data reduction was performed with the software package Iolite v.3.5 (Paton et al., 2011), combined with VizualAge (Petrus and Kamber, 2012). Average of seven metrics utilized within this study yields an age date of 96.4 Ma (see Tucker and colleagues19 for methods). Metrics include: 1) YSG, which is presented with a ±2σ; 2) YPP or the graphical age based on a histogram derived from Isoplot; 3) YDZ is presented with the Age and range (+ or −);

4) YC1σ (+3) is determined with the final age calculated via Age Pick (Arizona Geochron Lab) at a ±1σ error with a three (3) grain population minimum; 5) Weighted Average is determined with the Age, confidence, the number of grains rejected, MSWD and the overall probability; 6) YC2σ (+3) is determined with the final age calculated via Age Pick (Arizona Geochron Lab) at a

±2σ error with a three (3) grain population minimum; 7) TuffZirc is determined with the final age, confidence, and the Group Size (the number excluded against the grains used by the metric).

lll


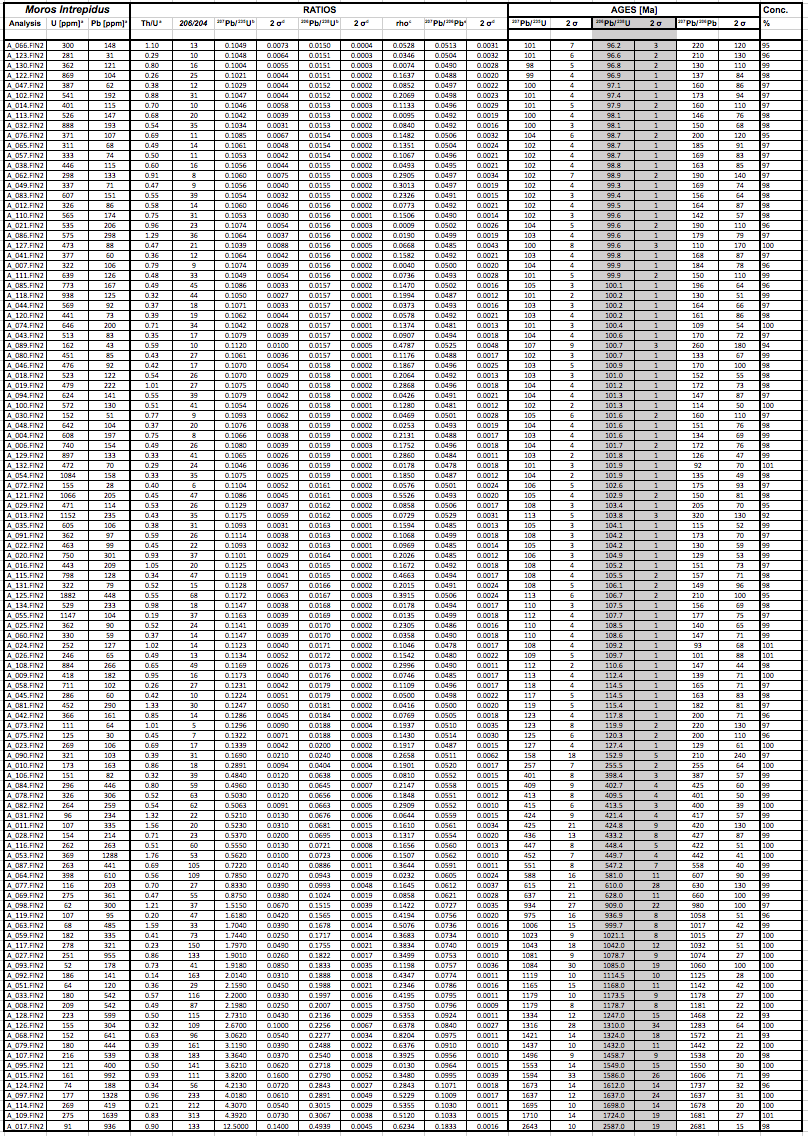


**Supplementary Table 1. Youngest single grain (YSG) results for all samples including isotope and concentration data**: Pb (in PPm) is Lead concentrations measured in parts per million; U (in PPM) is Uranium concentrations in parts per million; Th/U is the divided ratio of Uranium concentrations; Both Pb206/U235 and error calculated at ± 2σ and Pb206/U238 and error calculated at ± 2σ were used to calculate and Error Correction Value (Error Corr.). The ratio values for Pb206/U238, Pb207/Pb206, and Pb207/Pb206 with all error calculated at ± 2σ with the preferred age (Pb206/U238; highlighted in yellow) with error ± 2σ interpreted as YSG. Age is calculated by error propagation and included errors and U and U/Th concentration are within ~ 10% error.

# Supplementary Methods 2: Specimen measurements and additional images of tyrannosauroid teeth


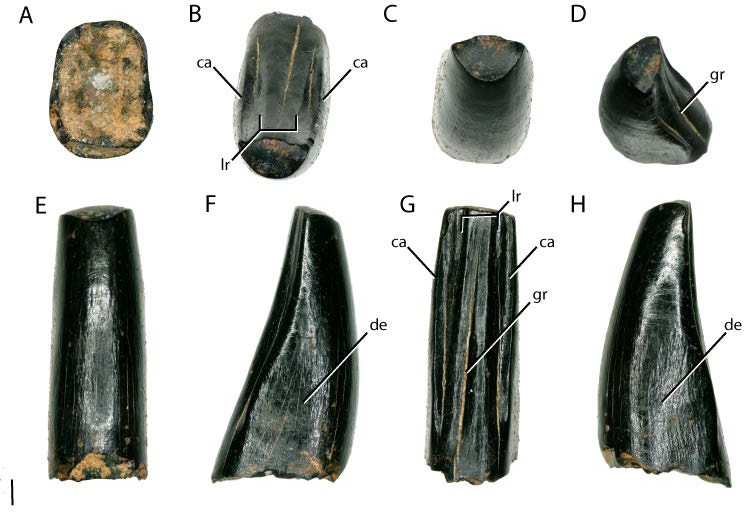


**Supplementary Figure 2: Isolated tyrannosauroid premaxillary tooth (NCSM 33393) from the lower Mussentuchit Member, Cedar Mountain Formation, USA.** (**a**) basal view; (**b**) lingual-apical view, showing lateral ridge; (**c**) labial-apical view; (**d**) oblique view, showing groove on lingual ridge; (**e**) lingual view; (**f**) mesial or distal view; (**g**) lingual view; (**h**) mesial or distal view. Abbreviations: ca, carina; de, mesial/distal depression; gr, groove dividing lingual ridge; lr, lingual ridge. Scale bar: 1mm.


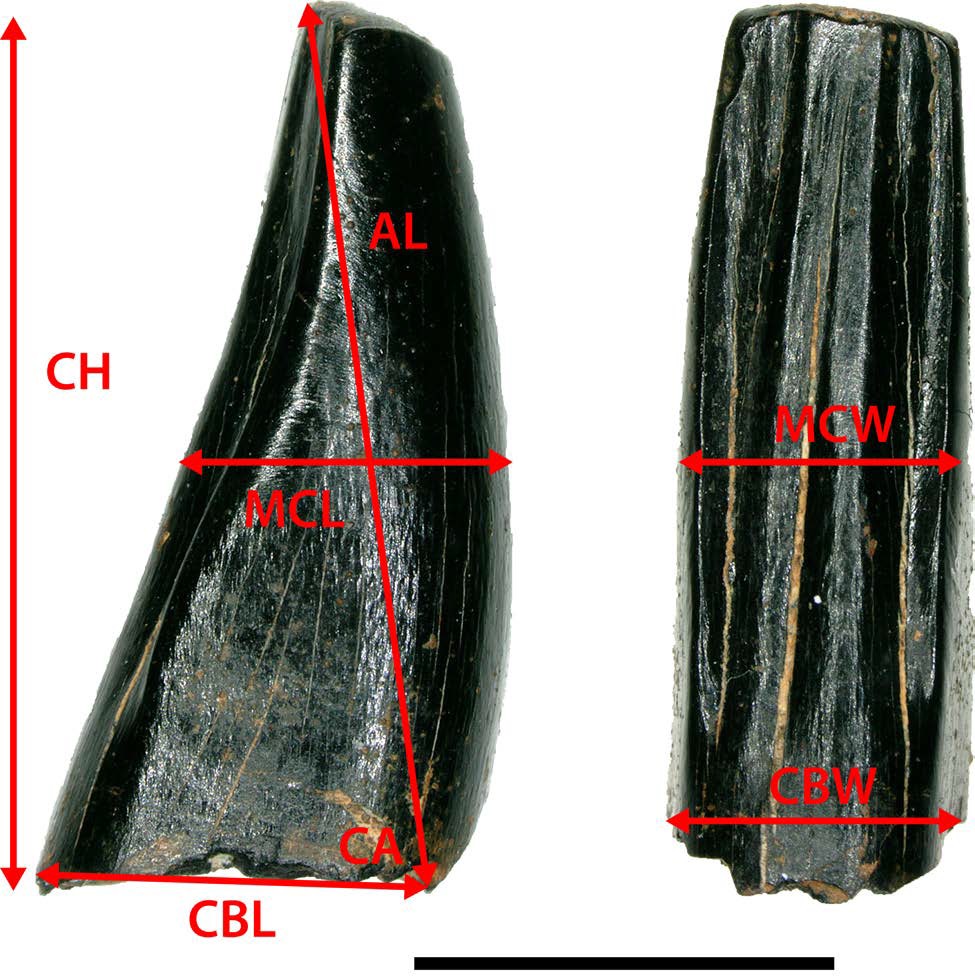


**Supplementary Figure 3: Select measurements of NCSM 33393.** Abbreviations: CBL, crown basal length; CBW, crown basal width; CH, crown height; AL, apical length; CA, crown angle; MCL, mid- crown length; MCW, mid-crown width (sensu Hendrickx20). Measurements: CBL, 5.65mm; CBW, 4.04mm; CH, 11.34mm; AL, 10.92mm; CA, 80.51mm; MCL, 4.53mm; MCW, 3.99mm. Scale bar 5 mm.


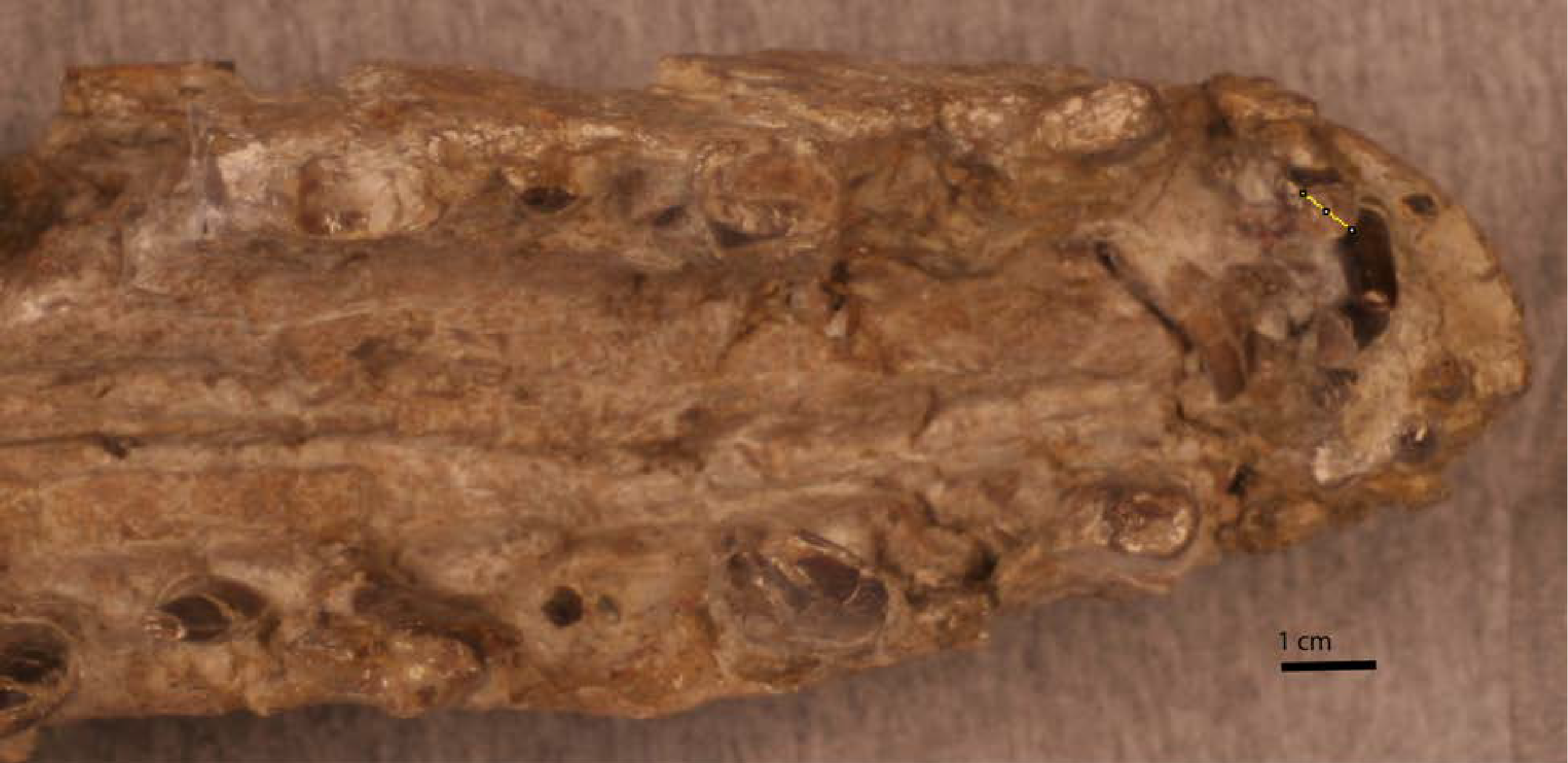


**Supplementary Figure 4: Skull of *Xiongguanlong baimoensis* (FRDC-GS JB16-2-1) in ventral view.** Yellow line showing position of measurement for mesiodistal basal crown width on third premaxillary tooth. Measurement used to estimate skull length of the individual from which the isolated tooth NCSM 33393 derives. Scale bar 1 cm.

|  | **Element** |  | | | | |
| --- | --- | --- | --- | --- | --- | --- |
| **Measurement** | *Femur* | *Tibia* | *MTIV* | *MTII* | *PIV-3* | *PIV-4* |
| L | 355* | 440* | 270* | - | 27.55 | 18.88 |
| PH | - | - | - | - | 14.52 | 11.91 |
| PW | - | - | - | - | 15.18 | 12.26 |
| MH | 32.57 | 26.19 | 17.94 | 24.13 | 9.09 | 7.75 |
| MW | 25.66 | 32.71 | 12.78 | 15.20 | 11.47 | 10.83 |
| MC | 105 | 93 | - | - | - | - |
| FCL | - | 70 | - | - | - | - |
| DH | - | - | 26.51 | - | 10.24 | - |
| DW | - | - | 17.16 | - | 11.67 | 11.28 |
| L/MW | 13.83 | 13.45 | 21.13 | - | - | - |
| MH/MW | 1.27 | 0.8 | 1.40 | - | - | - |
| DH/DW | - | - | 1.54 | - | - | - |

**Supplementary Table 2. Select measurements (in mm) of *Moros intrepidus* (NCSM 33392).** Abbreviations: dh, distal (craniocaudal or dorsal/plantar) height; dw, distal (transverse) width; fcl, fibular crest length; l, length; mh, midshaft (craniocaudal or dorsal/plantar) height; mc, midshaft circumference; mw, midshaft (transverse) width; ph, proximal (craniocaudal or dorsal/plantar) height; pw, proximal (transverse) width. Asterisk denotes estimated measurement.

|  | *cQE lower cQE upper cQE* | *AHR1985 CF2004* | *CM1992* |
| --- | --- | --- | --- |
| Mass estimate in kg | 76.537 57.380 95.695 | 52.719 84.531 | 64.282 |

**Supplementary Table 3. Mass estimates of the subadult specimen of *M*. *intrepidus* (NCSM 33392).** Abbreviations: cQE, lower cQE, uppercQE, upper and lower mass estimates derived from a mathematical correction of the quadrupedal equation (cQE) from Campione et al.21; *AHR1985,* Anderson, Hall-Martin & Russell22*; CF2004,* Christiansen & Farina23*; CM1992,* Campbell & Marcus24*.*

# Supplementary Methods 3: Additional figures illustrating the paleohistology of NCSM 33392


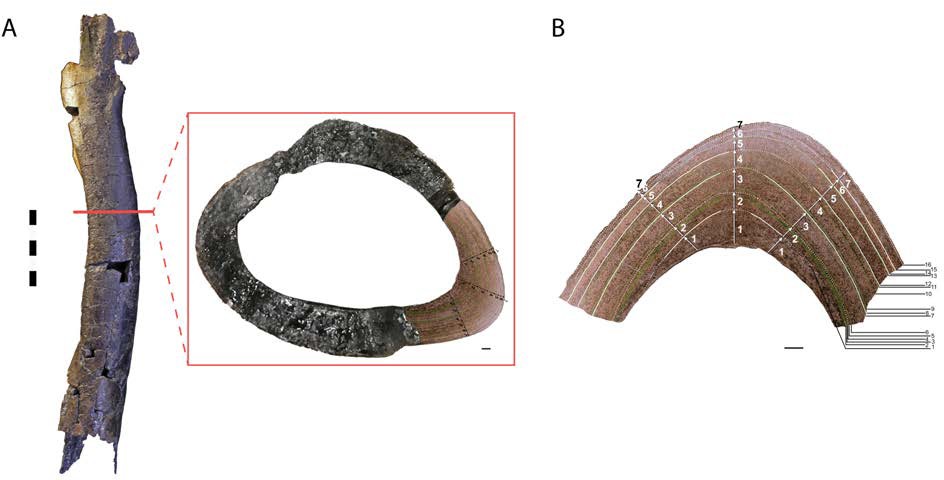


**Supplementary Figure 5: Location of paleohistological section of femur and details on growth markers (*M*. *intrepidus,* NCSM 33392).** (**a**) location of histological section on femur with inset in red showing proportion of cortical circumference represented by the histological section and cortical thickness relative to medulla, (**b**) partial mid-diaphyseal cross-section of the femur with tracings of 16 LAGs highlighted in green and white and interpretations of seven growth cycles numbered in white arrows. Growth cycles are determined from the main extinction patterns visible in polarized light, as can be viewed in the in-text manuscript figure (Fig. 2g). Scale bar for (a), 5 cm. Scale bar for insert and (b), 1 mm.


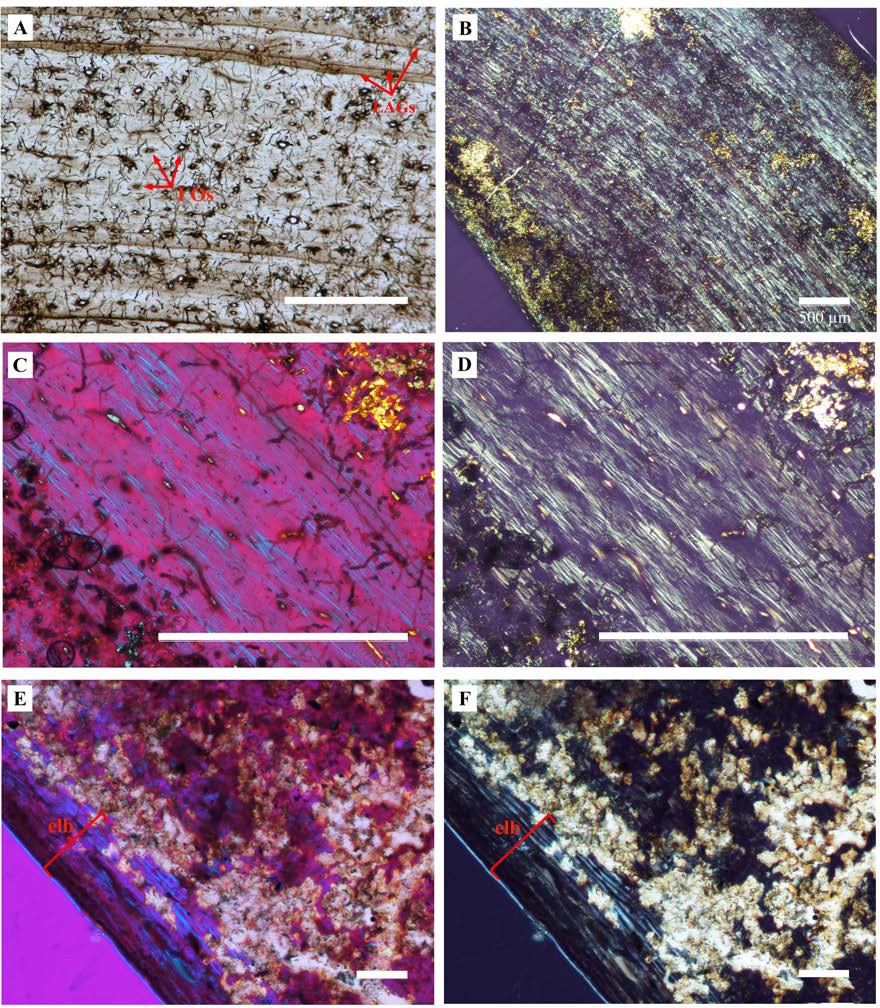


**Supplementary Figure 6: Bone microstructure of *M*. *intrepidus* (NCSM 33392). (a)** cortical bone tissue invaded by structures reminiscent of Wedl tunnels (fungal hyphae); vascularization is primarily longitudinal; and several lines of arrested growth are visible throughout the cortex (natural light). (**b**) cortical bone consists primarily of a parallel-fibered bone matrix; bone tissue presents a bulk anisotropy in polarized light. (**c**) close up of parallel-fibered bone matrix containing small longitudinal vascular canals (in polarized light with lambda filter). (**d**) same as “c”, in polarized light only. (**e**) thin layer of lamellar endosteal bone tissue on endosteal margin of bone wall (in polarized light with lambda filter). (**f**) same as “e” in polarized light only. Abbreviations: IOs, primary osteon; elb, endosteal lamellar bone; LAGs, lines of arrested growth. Scale bar (a-d) 500 μm, (e-f) 50 μm.

# Supplementary Discussion 1: Additional discussion on the anatomy and taxonomic identity of *M*. *intrepidus*

1. **Discussion of the femoral nutrient foramina in theropods**

Averianov and colleagues25 conflate the intertrochanteric nutrient foramen (or foramina) with the principle nutrient foramen of Madsen26, the latter of which is commonly observed on the proximal femur of theropods just distal to the lesser trochanter, by suggesting these features are homologous. As a result, Averianov and colleagues25 propose a new phylogenetic character describing the location of this foramen within or distal to the intertrochanteric fossa25. We note that these foramina are distinct features as evidenced by the presence of both an intertrochanteric and a principle nutrient foramen on the proximal femur of tyrannosauroids (e.g., *M*. *intrepidus* and *Tyrannosaurus rex* (BMRP 2006.4.4) and should not be coded as a single trait. However, we agree that the presence and position of these features is of taxonomic utility and that closer scrutiny and more thorough description of these foramina in theropods is warranted.

Among non-avian coelurosaurs, presence of an intertrochanteric nutrient foramen is limited to tyrannosaurs and ornithomimids (e.g., *Moros* [NCSM 33392]; *Tyrannosaurus*27; *Alioramus*28; *Gallimimu*s, IGM 100/14; Bissekty Fm. materials29; and possibly *Coelurus*, YPM 2010). The feature is absent in therizinosaurs (*Falcarius*), Alvarezsaurs (*Patagonykus*, MCF PVPH 37; *Mononykus*, MPD-C 107/6), and troodontids (MOR 553S). Among the sample we examined, only ornithomimids possess more than one intertrochanteric foramen (e.g., *Gallimimus*, MPD-C 100/14; NCMNS 33395) and thus the number of intertrochanteric nutrient foramen may be of taxonomic utility although a greater survey of specimens is required. Brusatte and colleagues28 note intraspecific variation in the presence of the intertrochanteric foramen within the tyrannosauroid *Alioramus* raising the possibility that this feature is too individually variable to be of use. In some theropods, (e.g., *Avimimus* and *Ceratosaurus*), there is an pneumatic fossa as opposed to a nutrient foramen in this region; however, the homology between these features is unclear. Thus, we hypothesize that the presence or absence of intertrochanteric foramina and the number of foramen has phylogenetic utility.

In contrast, it is the position, rather than the presence of the principle nutrient foramen that may be a taxonomic indicator. The principle nutrient foramen is located medial to the long axis of the lesser trochanter in immature and mature specimens of all species of tyrannosaurids we examined (e.g., *Moros*; *Gorgosaurus,* ROM 1247; and *Tyrannosaurus*27, as well as *Rahonavis* (UA8656), *Avimimus* (PIN 3907/1), and *Allosaurus*26; however, it is laterally positioned in therizinosaurs (*Falcarius*), ornithomimids (ROM 851, 852), and *Troodon* (MOR 553S), and sits directly on the axis in *Ceratosaurus*30 (UUVP 56). A femoral nutrient foramen is reported, yet not figured on *Khaan* and *Citipati*31 thus its identify and location in oviraptorosaurids is unclear. We hypothesize that a medial principle nutrient foramen is a synapmorphy of tyrannosaurs and supports referral of *M*. *intrepidus* to this clade as discussed below.


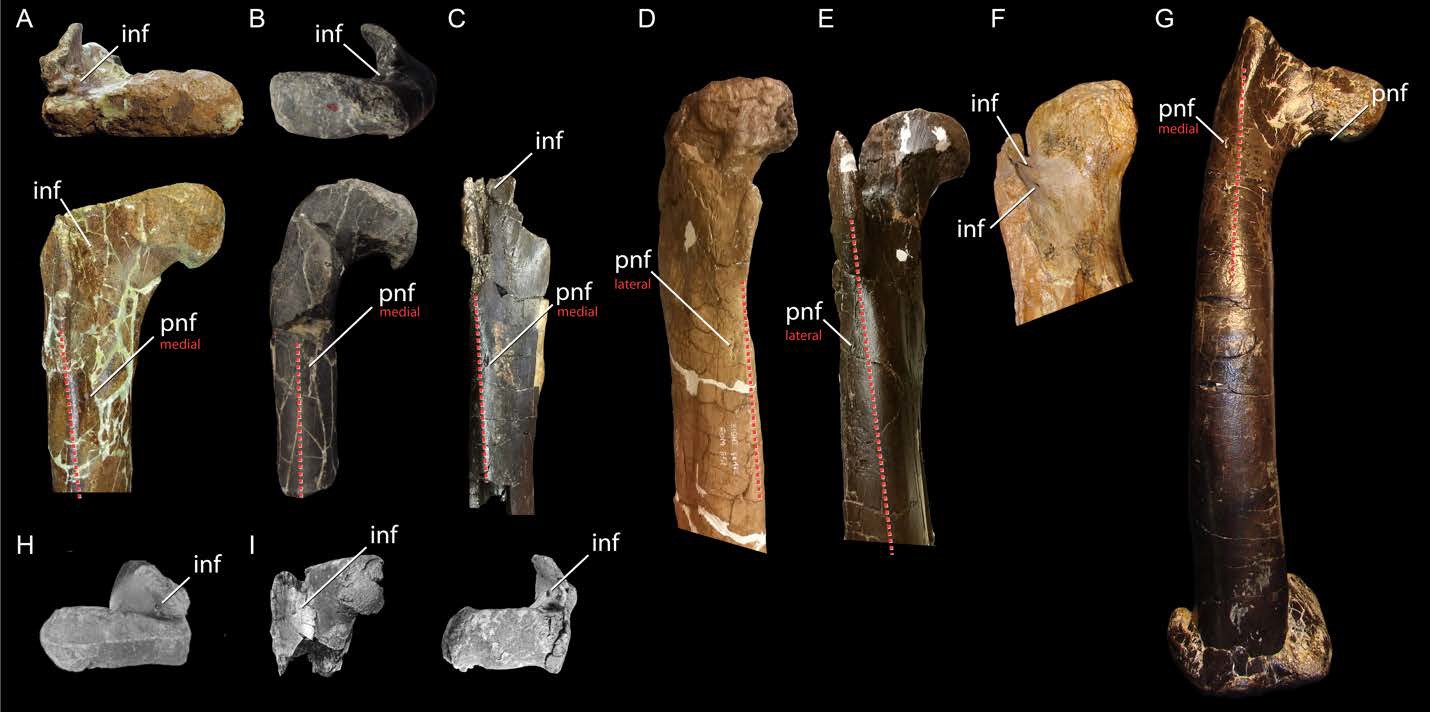


**Supplementary Figure 7: Position of nutrient foramina on theropod femora**. (**a**) *Tyrannosaurus rex* (BMRP 2006.4.4) in proximal (superior) and cranial (inferior, reversed) views; (**b**) *Coelurus fragilis* (YPM 2010) in proximal (superior) and cranial (inferior) views, (**c**) *Moros intrepidus* (NCSM 33392) in cranial view; (**d**) *Ornithomimus edmontonicus* (*Struthiomimus currelli*) (ROM 851) in lateral view; (**e**) *Ornithomimus edmontonicus* (ROM 852) in cranial view; (**f**) *Gallimimu*s, MPC- D 100/14 in cranial view; (**g**) *Troodon* (MOR 553S) in cranial view, reversed; (**h**) *Tyrannosaurus rex* (reproduced from Brochu27) in proximal view; (**g**) *Alioramus* (reproduced from Brusatte and colleagues28) in cranial (left) and proximal (right) views. Abbreviations: inf, intratrochanteric nutrient foramen; pnf, principle nutrient foramen. Images not to scale.

# Distinguishing *M*. *intrepidus* from ornithomimosaurs and oviraptorosaurs

*Moros intrepidus* is referable to a non-maniraptoran coelurosaurian theropod based on the presence of an alariform lesser trochanter; deep cleft separating the greater and lesser femoral trochanters; well-developed fourth trochanter, absence of a posterior trochanter on the femur, and a fully arctometatarsalian pes. A variety of features preclude an oviraptorosaurian assignment for *M*. *intrepidus* including a fibular shaft that is appressed to the cranially surface of the tibial shaft throughout its length, preventing the lateral aspect of the ascending process of the astragalus from contacting the lateral tibia32. However, differentiation from ornithomimosaurs is less obvious. The small and unusually gracile bauplan of *M*. *intrepidus* relative to other Late Cretaceous tyrannosauroids, paucity of well- preserved specimens of mid-Cretaceous coelurosaurians for comparison, poor preservation of NCSM 33392, and generally similar hind limb morphology of tyrannosauroid and ornithomimosaurians, especially when disregarding features expected to strongly covary with body size, lends us to specifically address the hypothesis that *M*. *intrepidus* represents an ornithomimid.

With regard to the preserved morphology of the femur of NCSM 33392, both ornithomimids and tyrannosaurs possess an alariform lesser trochanter that rises directly from the most pinched aspect of the cranial shaft; a lateral depression on the caudomedial femoral shaft bounded caudally by a mound-like trochanteric crest and laterally by a dorsoventral ridge on the lesser trochanter; intertrochanteric and/or principle nutrient foramen; slightly concave caudal aspect of the femoral shaft in the region of the fourth trochanter; overlap between the fourth trochanter and the lesser trochanter; a cranomedially/caudolaterally expanded cross-section of the distal femur; pronounced mediodistal and ectepicondylar crests; and a flat to slightly concave extensor fossa. Some of these features (e.g., development of the trochanteric crest and dorsoventral ridge on the lesser trochanter; degree of depression of the extensor fossa) are somewhat more pronounced on Late Cretaceous tyrannosaurids (e.g., *Gorgosaurus*, ROM 1247; *Albertosaurus*, ROM 807); however, the degree of development could reasonably be size related. Two features of the femur argue against an ornithomimid referral. The base of the groove separating the lesser from greater trochanters on NCMS 33392 is indicative of a shallower, narrow cleft; this is opposed to the condition observed in ornithomimids, in which the groove is distally extensive and the lesser trochanter extends cranially on an expanded “neck”. Moreover, the principle foramen pierces the femoral shaft medial to the long axis of the lesser trochanter on NCMS 33392. As described above, this feature is lateral to this axis on all Late Cretaceous ornithomimids specimens we observed (Supplementary Fig. 7).

Tibial morphology of NCMS 33392 is consistent with those of select ornithomimids and tyrannosaurs in possessing a sharply defined facet for the fibula and a fibular shaft that shifts abruptly from lateral to the fibular crest to the cranial shaft and remains cranially oriented to brace the ascending process of the astragalus. This feature is present on *Tyrannosaurus* (BMRP 2002.4.) and *Ornithomimus edmontonicus* (ROM 852), yet absent from other specimens referred to this *O*. *edmontonicus* (ROM 797, 851), in which case the fibular shaft transitions back to a lateral position at the contact with the ascending process, and several tyrannosaurids (e.g., *Gorgosaurus,* ROM 1247), in which the fibular facet is not well defined.

Both ornithomimosaurs and tyrannosaurs possess an arctometatarsalian pes. Proportionally, the slender metatarsus of *M*. *intrepidus* (NCSM 33392) clusters closer to ornithomimosaurs than tyrannosaurids (Supplementary Fig. 12). However, the general morphology of the *M*. *intrepidus* permits referral to Tyrannosauroidea. Features of the fourth metatarsus of *M*. *intrepidus* that argue against an ornithomimosaur referral are numerous and include: the highly symmetrical “D” shaped cross- section of the shaft (it is rounded or askew in ornithomimosaurs); the high degree of lateral deflection of the of the distal aspect (Supplementary Fig. 8) (relatively straight in ornithomimosaurs); the presence of a concavity emarginated by a raised ridge on the plantar surface and a well-developed scar for the *M*. *gastroccnemius lateralis* (absent on ornithomimosaurs) (Supplementary Fig. 8); a ventral bend to the distal aspect in

lateral view creating a concave margin (absent on ornithomimosaurs) (Supplementary Fig. 9); and a lateral groove delineating the craniolateral margin of the distal articular surface and absence of an isolated collateral ligament pit (absent on ornithomimosaurs) (Supplementary Fig. 9).


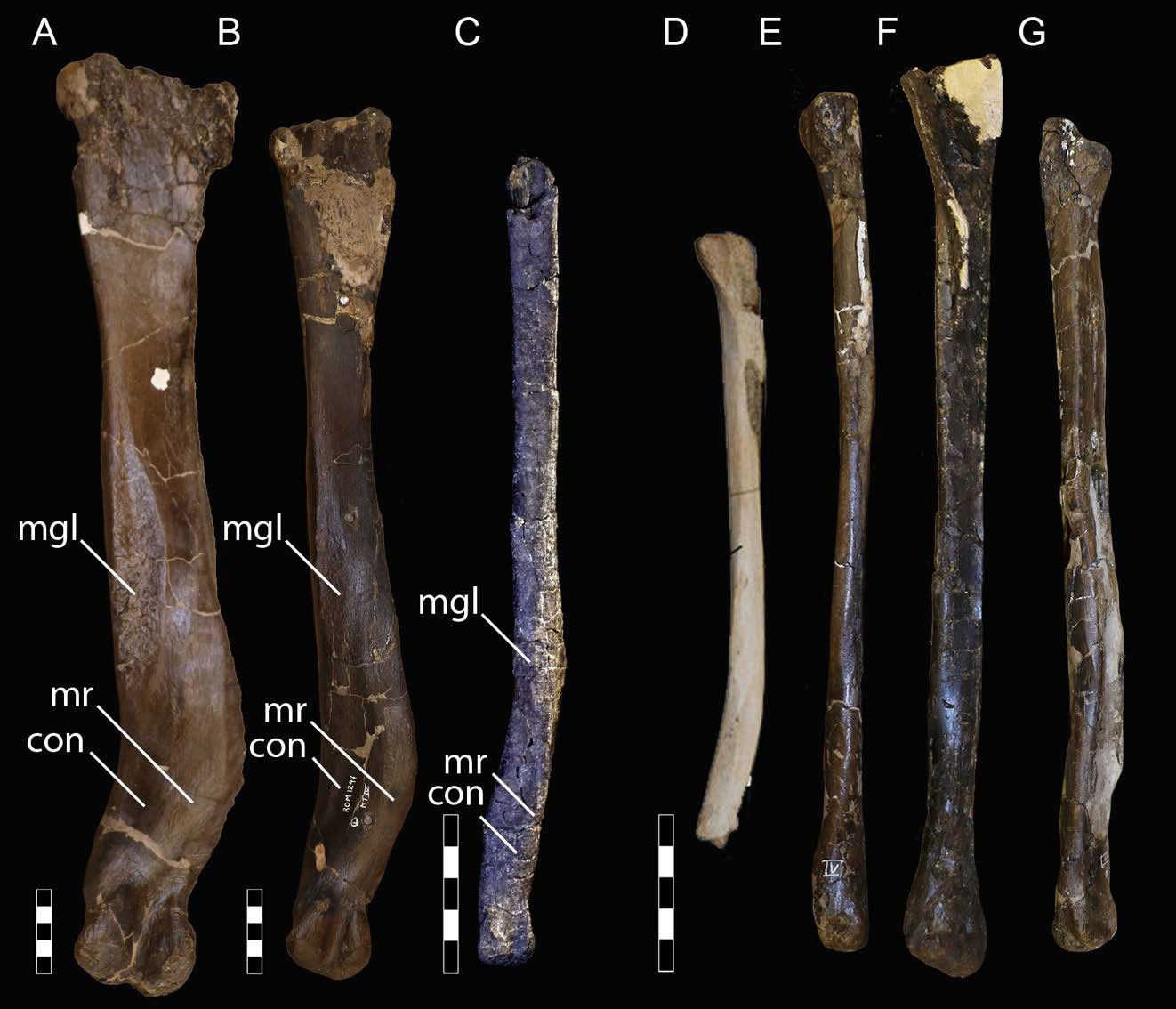


**Supplementary Figure 8: Comparative anatomy of the MTIV of tyrannosauroids and ornithomimosaurs in plantar view**. (**a**) *Albertosaurus sarcophagus* (ROM 807); (**b**) *Gorgosaurus libratus* (ROM 1247); (**c**) *Moros intrepidus* (NCSM 33392); (**d**) *Aepyornithomimus tugrikinensis* (MPC-D 100/130); (**e**) *Ornithomimus edmontonicus* (*Dromiceiomimus breveritus*) (ROM 797, left pes); (**f**) *Rativates* (ROM 1790); (**g**) *Ornithomimus edmontonicus* (*Dromiceiomimus breveritus*) (ROM 797, right pes). Abbreviations: con, concavity just proximal to distal condyles; mr, medial ridge originating at proximal junction point of distal condyles; mgl, scar for the *M*. *gastroccnemius lateralis*. Scale bar 5 cm.


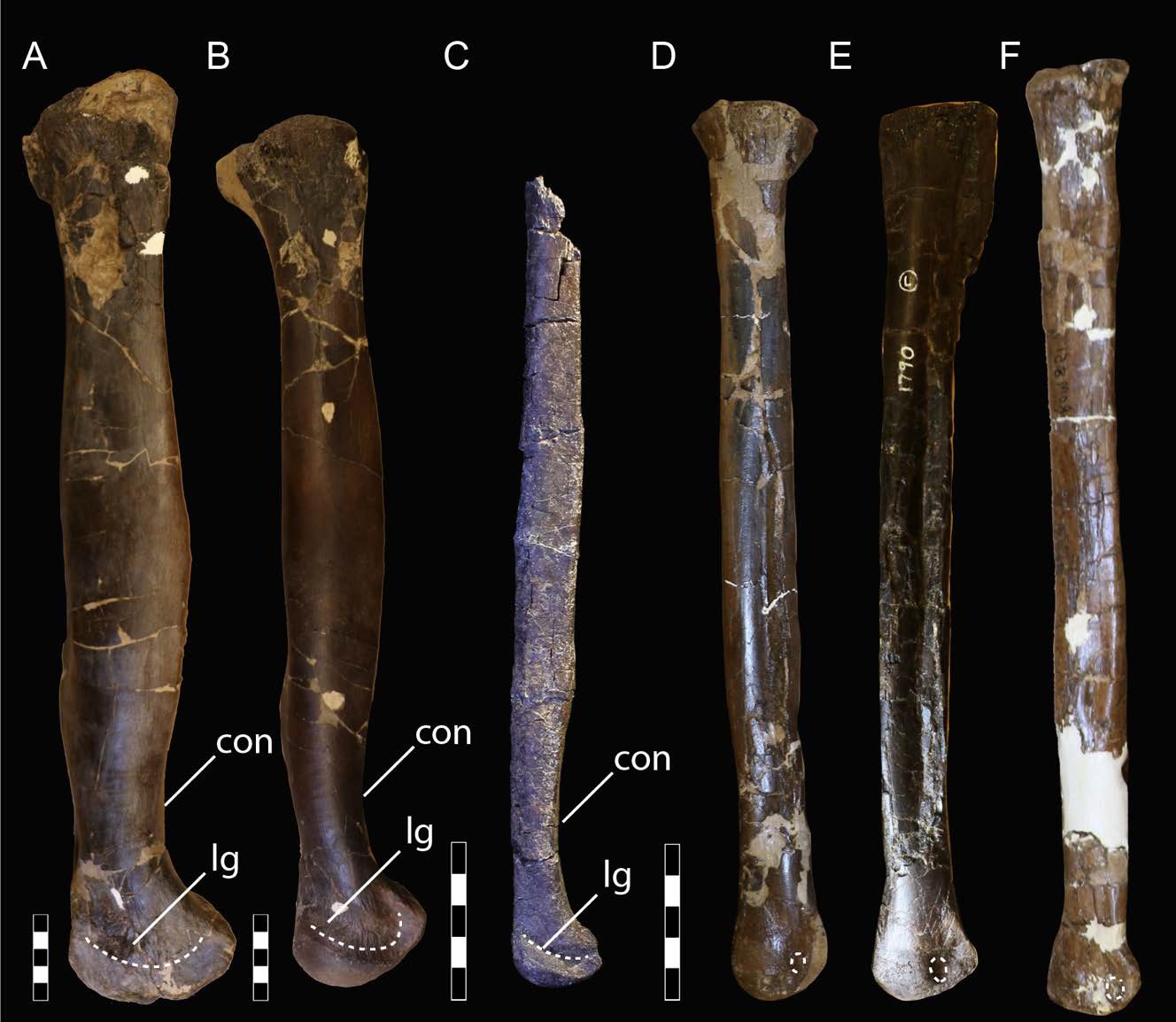


**Supplementary Figure 9: Comparative anatomy of the MTIV of tyrannosauroids and ornithomimosaurs in lateral view.** (**a**) *Albertosaurus sarcophagus* (ROM 807); (**b**) *Gorgosaurus libratus* (ROM 1247); (**c**) *Moros intrepidus* (NCSM 33392); (**d**) *Ornithomimus edmontonicus* (*Dromiceiomimus breveritus*) (ROM 797); (**e**) *Rativates* (ROM 1790); (**f**) *Ornithomimus edmontonicus* (*Struthiomimus currelli*) (ROM 851). Abbreviations: con, concavity just proximal to distal condyles; lg, lateral groove on distal articular surface. Scale bar 5 cm.


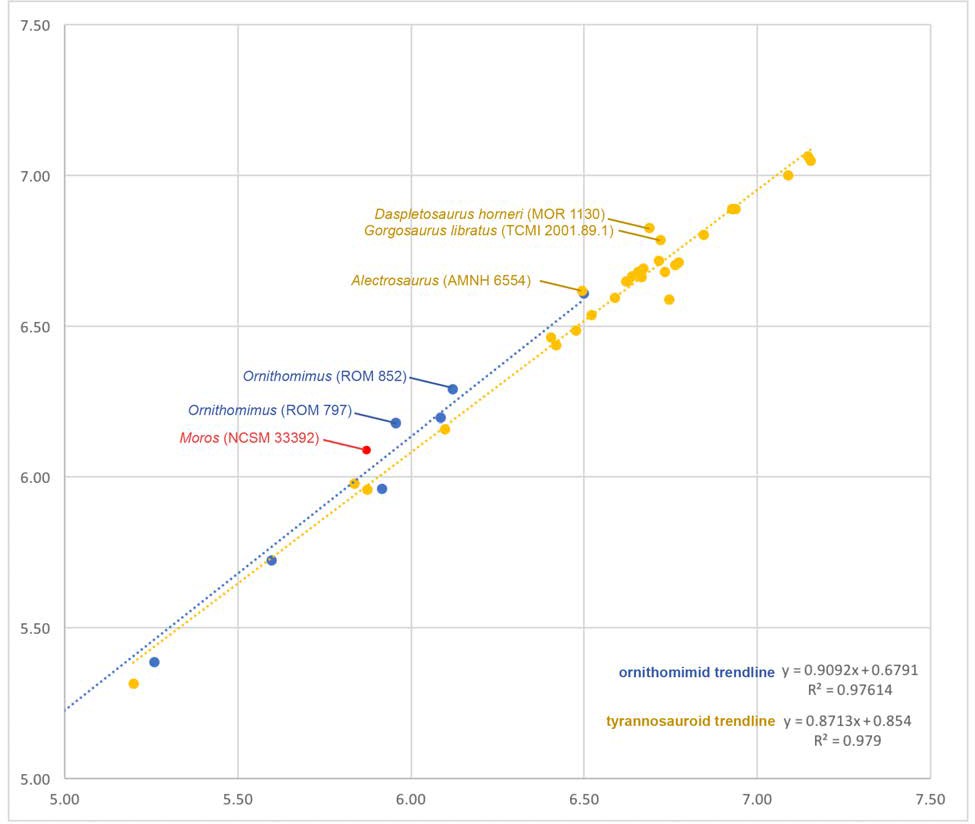


# Supplementary Figure 10: OLS linear regression of proximodistal femoral length (x-axis) against proximodistal femoral tibial length (y-axis) in tyrannosauroids, ornithomimosaurs, and *M*. *intrepidus* (NCSM 33392).


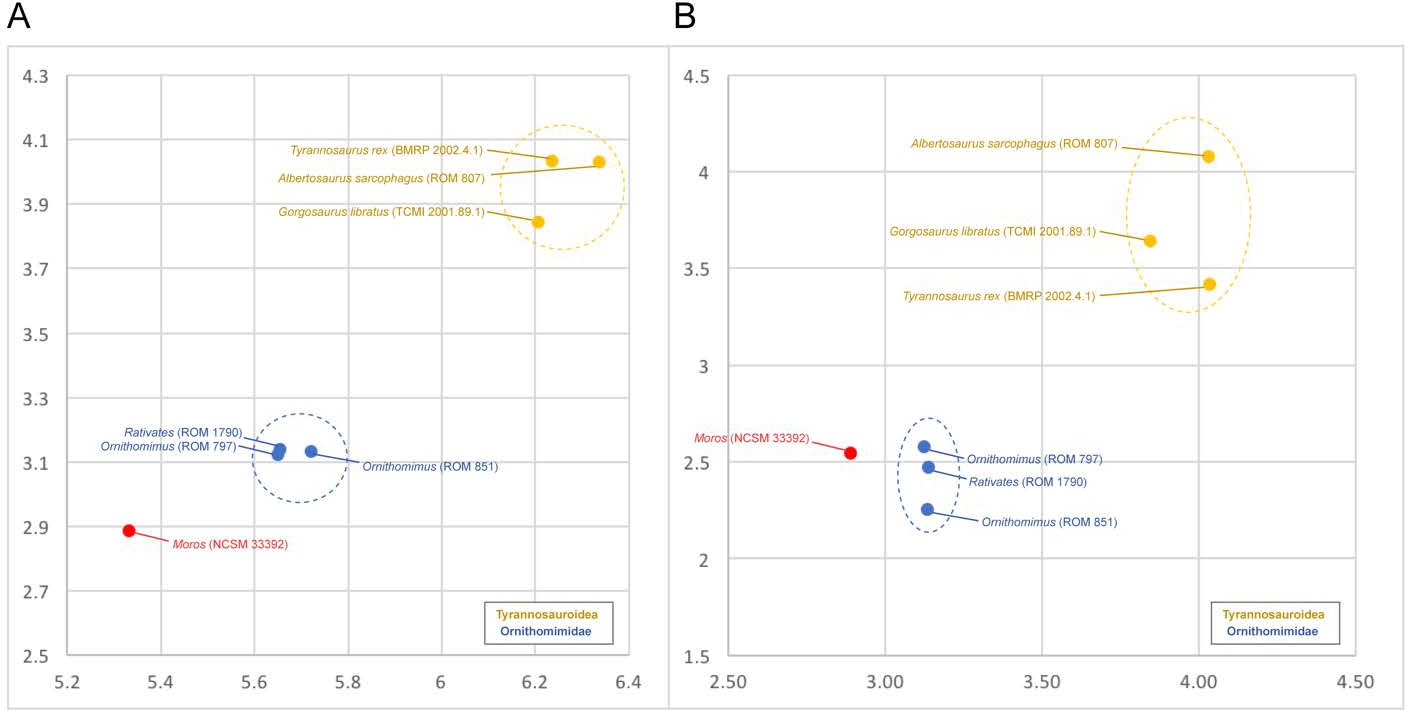


**Supplementary Figure 11: Biplot of metatarsal proportions in tyrannosaurids, ornithomimids, and *M*. *intrepidus* (NCSM 33392).** (**a**) MTIV proximodistal length (x-axis) to MTIV mid-shaft craniocaudal height (y-axis); and (**b**) MTIV mid-shaft craniocaudal height (x-axis) to MTIV mid- shaft transverse width (y-axis).

|  | **Specimen #** | **Measurement Reference** | **Femur Length** | **Tibia Length** | **log FL** | **log TL** |
| --- | --- | --- | --- | --- | --- | --- |
| **Tyrannosauroidea** |  |  |  |  |  |  |
| *Tanycolagreus topwilsoni* | TPII 2000-09-29 | Carpenter et al. 2005^33^ | 356 | 387 | 5.87 | 5.96 |
| *Dilong paradoxus* | IVPP V14243 | Xu et al. 2004^34^ | 181 | 203 | 5.20 | 5.31 |
| *Guanlong wucaii* | IVPP V 14531 | Persons & Currie 2016^35^ | 343 | 395 | 5.84 | 5.98 |
| *Yutyrannus huali* | ZCDM V5000 | Xu et al. 2012^26^ | 850 | 725 | 6.75 | 6.59 |
| *Yutyrannus huali* | ZCDM V5001 | Xu et al. 2012^36^ | 650 | 655 | 6.48 | 6.48 |
| *Yutyrannus huali* | ELDM V1001 | Xu et al. 2012^36^ | 613 | 623 | 6.42 | 6.43 |
| *Alectrosaurus olsoni* | AMNH 6554 | Persons & Currie 2016^35^ | 727 | 730 | 6.59 | 6.59 |
| *Alectrosaurus olsoni* | AMNH 6554 | https://paleobiodb.org | 661 | 748 | 6.49 | 6.62 |
| *Albertosaurus sarcophagus* | NMC 11315 | Persons & Currie 2016^35^ | 680 | 690 | 6.52 | 6.54 |
| *Albertosaurus sarcophagus* | TMP 1981.10.1 | Persons & Currie 2016^35^ | 940 | 900 | 6.85 | 6.80 |
| *Albertosaurus sarcophagus* | ROM 807 | https://paleobiodb.org | 1020 | 980 | 6.93 | 6.89 |
| *Albertosaurus sarcophagus* | TMP 1985.98.1 | Persons & Currie 2016^35^ | 750 | 770 | 6.62 | 6.65 |
| *Appalachiosaurus montgomeriensis* | RMM 6670 | https://paleobiodb.org | 786 | 781 | 6.67 | 6.66 |
| *Daspletosaurus torosus* | MOR 590 | Persons & Currie 2016^35^ | 865 | 815 | 6.76 | 6.70 |
| *Daspletosaurus horneri* | MOR 590 | Carr et al. 2017^37^ | 875 | 820 | 6.77 | 6.71 |
| *Daspletosaurus horneri* | MOR 1130 | Carr et al. 2017^37^ | 802.7 | 920 | 6.69 | 6.82 |
| *Dryptosaurus aquilungensis* | TMP 84.181.2 cast of ANSP 9995 | Persons & Currie 2016^35^ | 778 | 796 | 6.66 | 6.68 |
| *Gorgosaurus libratus* | AMNH 5423 | Persons & Currie 2016^35^ | 605 | 640 | 6.41 | 6.46 |
| *Gorgosaurus libratus* | TCMI 2001.89.1 | Persons & Currie 2016^35^ | 830 | 885 | 6.72 | 6.79 |
| *Gorgosaurus libratus* | FMNH PR. 2211 | Persons & Currie 2016^35^ | 445 | 472 | 6.10 | 6.16 |
| *Gorgosaurus libratus* | TMP 91.163.0001 | Persons & Currie 2016^35^ | 755 | 770 | 6.63 | 6.65 |
| *Gorgosaurus libratus* | NMC 2120 | Persons & Currie 2016^35^ | 1030 | 980 | 6.94 | 6.89 |
| *Gorgosaurus libratus* | ROM 1247 | direct measurement | 840 | 795 | 6.73 | 6.68 |
| *Tyrannosaurus rex* | LACM 23845 | Persons & Currie 2016^35^ | 825 | 825 | 6.72 | 6.72 |
| *Tyrannosaurus rex* | RTMP 81.12.1 (cast of 9950) | Persons & Currie 2016^35^ | 1200 | 1095 | 7.09 | 7.00 |
| *Tyrannosaurus rex* | MOR 555 | Persons & Currie 2016^35^ | 1280 | 1150 | 7.15 | 7.05 |
| *Tyrannosaurus rex* | CMN 9380 (cast of AMNH 973) | Persons & Currie 2016^35^ | 1269 | 1166 | 7.15 | 7.06 |
| *Tyrannosaurus rex* | BMRP 2002.4.1 | direct measurement of cast | 790 | 805 | 6.67 | 6.69 |
| *Tarbosaurus baatar* | MPC-D 100/63 | Persons & Currie 2016^35^ | 1020 | 980 | 6.93 | 6.89 |
| *Moros intrepidus* | NCSM 33392 | Estimated, this paper | 355 | 440 | 5.87 | 6.09 |
| **Ornithomimidae** |  |  |  |  |  |  |
| *Ornithomimus edmontonicus*  *(Struthiomimus currelli)* | ROM 851 | direct measurement | 439.78 | 490.37 | 6.09 | 6.20 |
| *Ornithomimus edmontonicus*  *(Dromiceiomimus breveritus)* | ROM 797 | direct measurement | 385.81 | 481.98 | 5.96 | 6.18 |
| *Ornithomimus edmontonicus* | ROM 852 | direct measurement | 455.38 | 539.07 | 6.12 | 6.29 |
| *Nedcolbertia justinhoffmani* | TMP 96.90.2 (cast of CEUM 5071) | Persons & Currie 2016^35^ | 145 | 199 | 4.98 | 5.29 |
| *Gallimimus bullatus* | MPD-C 100/10 | Osmólska et al. 1972^38^ | 192 | 218 | 5.26 | 5.38 |
| *Gallimimus bullatus* | z.pal.no.mg.d-1/94 | Osmólska et al. 1972^38^ | 270 | 306 | 5.60 | 5.72 |
| *Gallimimus bullatus* | MPD-C 100/11 | Osmólska et al. 1972^38^ | 665 | 740 | 6.50 | 6.61 |
| *Garudimimus brevipes* | MPD-C 100/13 | Kobayashi & Barsbold 2005^39^ | 371 | 388 | 5.92 | 5.96 |

# Supplementary Table 3: Femur and tibia lengths of tyrannosauroids and ornithomimids.

|  | **Specimen #** | **L** | **SH** | **SW** | **DH** | **DW** | **L/SW** | **SH/SW** | | **DH/DW** | |
| --- | --- | --- | --- | --- | --- | --- | --- | --- | --- | --- | --- |
| **Tyrannosauroidea** |  |  |  |  |  |  |  |  |  | |  |
| *Albertosaurus sarcophagus* | ROM 807 | 565.2 | 56.26 | 59.23 | 114.93 | 69.5 | 9.54 | 0.95 | 1.65 | |  |
| *Gorgosaurus libratus* | ROM 1247 | 495.4 | 46.71 | 38.22 | 80.51 | 48.91 | 12.96 | 1.22 | 1.65 | |  |
| *Tyrannosaurus rex* | BMRP 2002.4.1 | 510.48(r) | 56.52(r) | 30.55(r) | 57.6(r) | 56.17(r) | 16.45 | 1.85 | 1.03 | |  |
| *Moros intrepidus* | NCSM 33392 | 207 | 17.94 | 12.78 | 26.51 | 24.58 | 21.13 | 1.40 | 1.55 | |  |
| **Ornithomimidae** |  |  |  |  |  |  |  |  |  | |  |
| *Ornithomimus edmontonicus*  *(Struthiomimus currelli)* | ROM 851 | 305.45 | 22.91* | 9.55* | 34.9 | 16.78 | 32 | 2.4 | 2.08 | |  |
| *Ornithomimus edmontonicus*  *(Dromiceiomimus breveritus)* | ROM 797 | 284.6 | 22.7 | 13.17 | 29.4** | 18.84 | 21.6 | 1.72 | 1.3 | |  |
| *Rativates evadens* | ROM 1790 | 285.76 | 23.06 | 11.86 | 33.29 | 20.2 | 24.1 | 1.94 | 1.21 | |  |

**Supplementary Table 4: Metatarsal measurements used to assess the relative limb proportions of *M*. *intrepidus* in comparison to other Late Cretaceous tyrannosauroids and ornithomimids.** Abbreviations: L, length of metatarsal IV; SH, craniocaudal height of the shaft of MTIV; SW, transverse width of the shaft of MTIV. *crushed. **incomplete

# Supplementary Discussion 2: Phylogenetic and Temporal Data

|  | **Age Reference** | **Age (Ma)** |
| --- | --- | --- |
| *Albertosaurus sarcophagus* | Fowler 2017^40^ | 70.9–73.5 |
| *Appalachiosaurus montgomeriensis* | Carr et al. 2005^41^ | 78 |
| *Daspletosaurus torosus* | Fowler 2017^40^, Carr et al., 2017^37^ | 76.1–77.03 |
| *Daspletosaurus horneri* | Fowler 2017^40^, Carr et al., 2017^37^ | 74.38–76.02 |
| *Dryptosaurus aquilungensis* | Carpenter et al. 1997^42^ | 66–70 |
| *Gorgosaurus libratus* | Fowler 2017^40^ | 76–77.03 |
| *Tanycolagreus topwilsoni* | Trujillo et al. 2006^43^ | 147–155 |
| *Tyrannosaurus rex* | Fowler 2017^42^ | 66–67 |
| *Dilong paradoxus* | Xu et al. 2004^34^ | 128–139 |
| *Guanlong wucaii* | Xu et al. 2006^44^ | 157–164 |
| *Yutyrannus huali* | Xu et al. 2012^36^ | 128–139 |
| *Alectrosaurus olseni* | Averianov 2002^45^ | 89.8–93.9 |
| *Moros intrepidus* | This paper | 93–96.3 |
| *Stokesosaurus clevelandi* | Trujillo et al. 2006^43^ | 147–155 |
| *Teratophoneus curriei* | Fowler 2017^40^ | 75.51 |
| *Lythronax argestes* | Fowler 2017^40^ | 79.9–80 |
| *Bistahieversor sealeyi* | Fowler 2017^40^ | 74.5–75 |
| *Juratyrant langhami* | Brusatte and Benson 2013^46^ | 148–152 |
| *Tarbosaurus baatar* | Loewen et al. 2013^47^ | 68–70.6 |
| *Xiongguanlong baimoensis* | Li et al. 2009^48^ | 100.5–125 |
| *Alioramus* | Brusatte et al. 2012^28^ | 68–70.6 |
| *Timerlengia euotica* | Brusatte et al. 2016^49^ | 90–92 |
| *Kileskus aristotocus* | http://www.paleobiodb.org | 166–168 |
| *Zuchengtyrannus magnus* | http://www.paleobiodb.org | 73.5–83.5 |
| *Nanuqsaurus holgundi* | http://www.paleobiodb.org | 69.1 |
| *Qianzhousaurus sinensis* | http://www.paleobiodb.org | 66–72 |
| *Sinotyrannus kazuoensis* | http://www.paleobiodb.org | 113–125 |
| *Proceratosaurus bradleyi* | http://www.paleobiodb.org | 166–168 |
| *Eotyrannus lengi* | http://www.paleobiodb.org | 125–136 |
| *Aviatyrannus jurassica* | http://www.paleobiodb.org | 152.1–157.3 |

**Supplementary Table 5. Temporal data for Figure 5.**


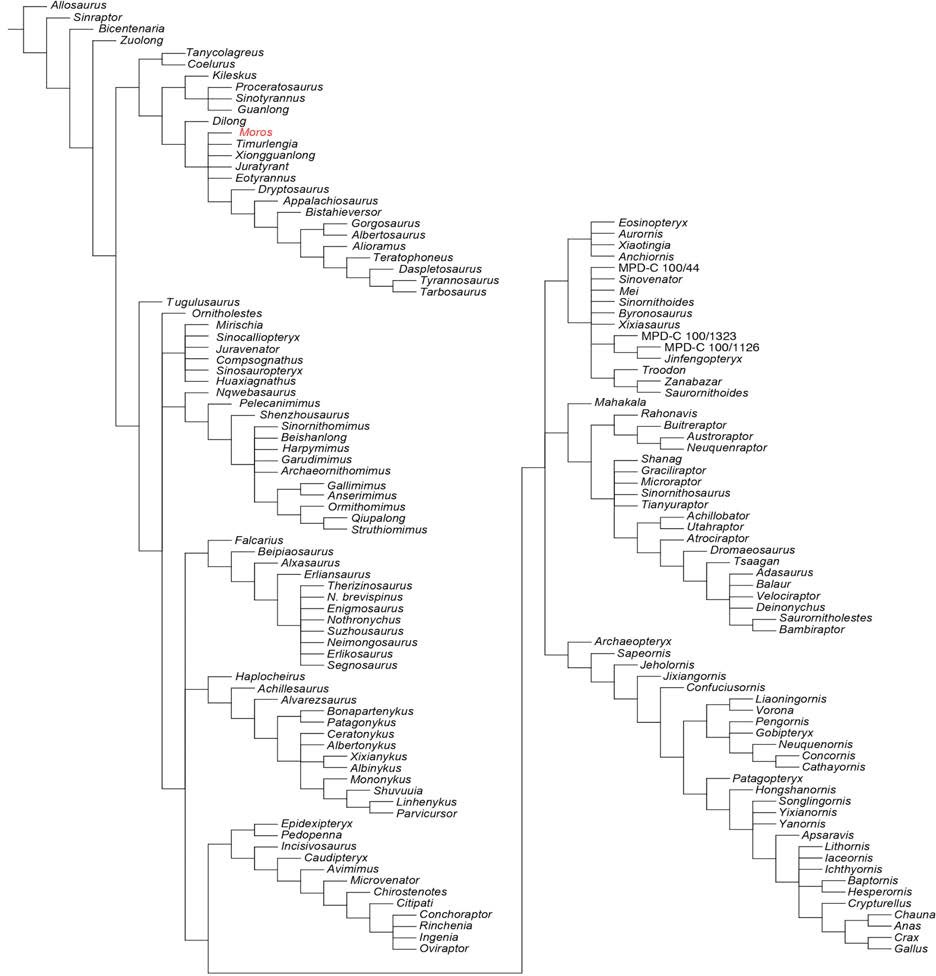


**Supplementary Figure 12: Strict consensus tree resulting from the inclusion of *M*. *intrepidus* (NCSM 33392) in the updated matrix of Brusatte and colleagues49.**


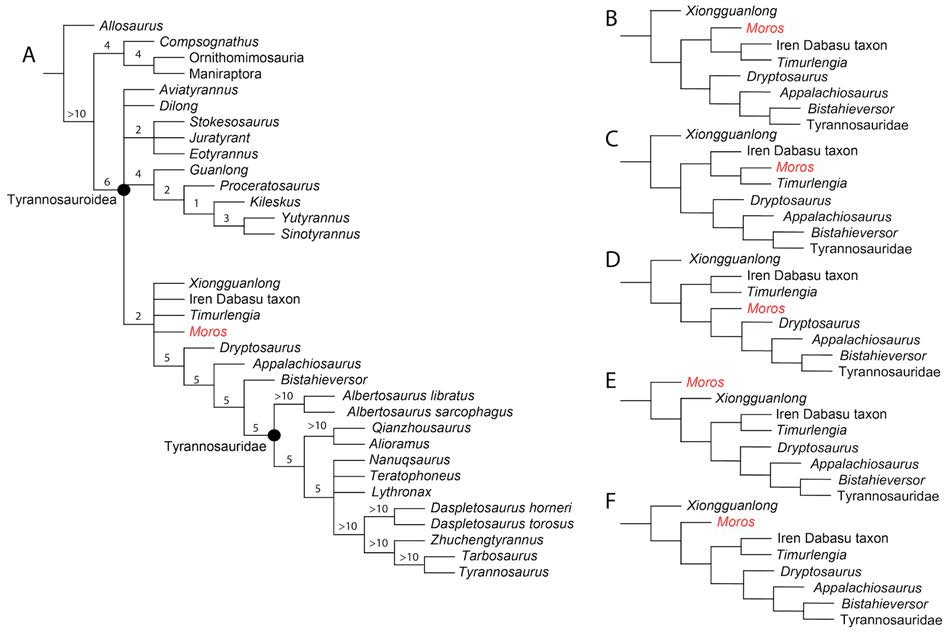


**Supplementary Figure 13: Hypothesized relationships of Tyrannosauroidea including *M*. *intrepidus* (NCSM 33392) in the updated matrix of Carr and colleagues37.** (**a**) strict consensus of 36 MPTs recovering *Moros* as an intermediate tyrannosauroid in a polytomy with other mid- Cretaceous tyrannosaur species (i.e., *Xiongguanlong*, *Timurlengia,* and the Iren Dabasu Formation taxon), and a clade containing *Dryptosaurus*, *Appalachiosaurus*, *Bistahieversor*, and *Tyrannosauridae*; (**b-f**) alternative hypotheses of the relationships of *Moros* and other intermediate tyrannosauroids as recovered amongst all MPTs. A subclade containing *Timurlengia*, the Iren Dabasu taxon, and *Moros* is recovered in 61% of MPTs (in 36% of MPTs this clade also contains *Xiongguanlong*). The remaining 39% of MPTs posit these four taxa in six disparate topologies forming successive outgroups to *Dryptosaurus* plus all more specialized tyrannosauroids. Agreement subtrees identify a substructure in which *Timurlengia* and the Iren Dabasu taxon consistently form a subclade and indicate that lability of *Moros* and *Xiongguanlong* is causing uncertainty in tree topology.


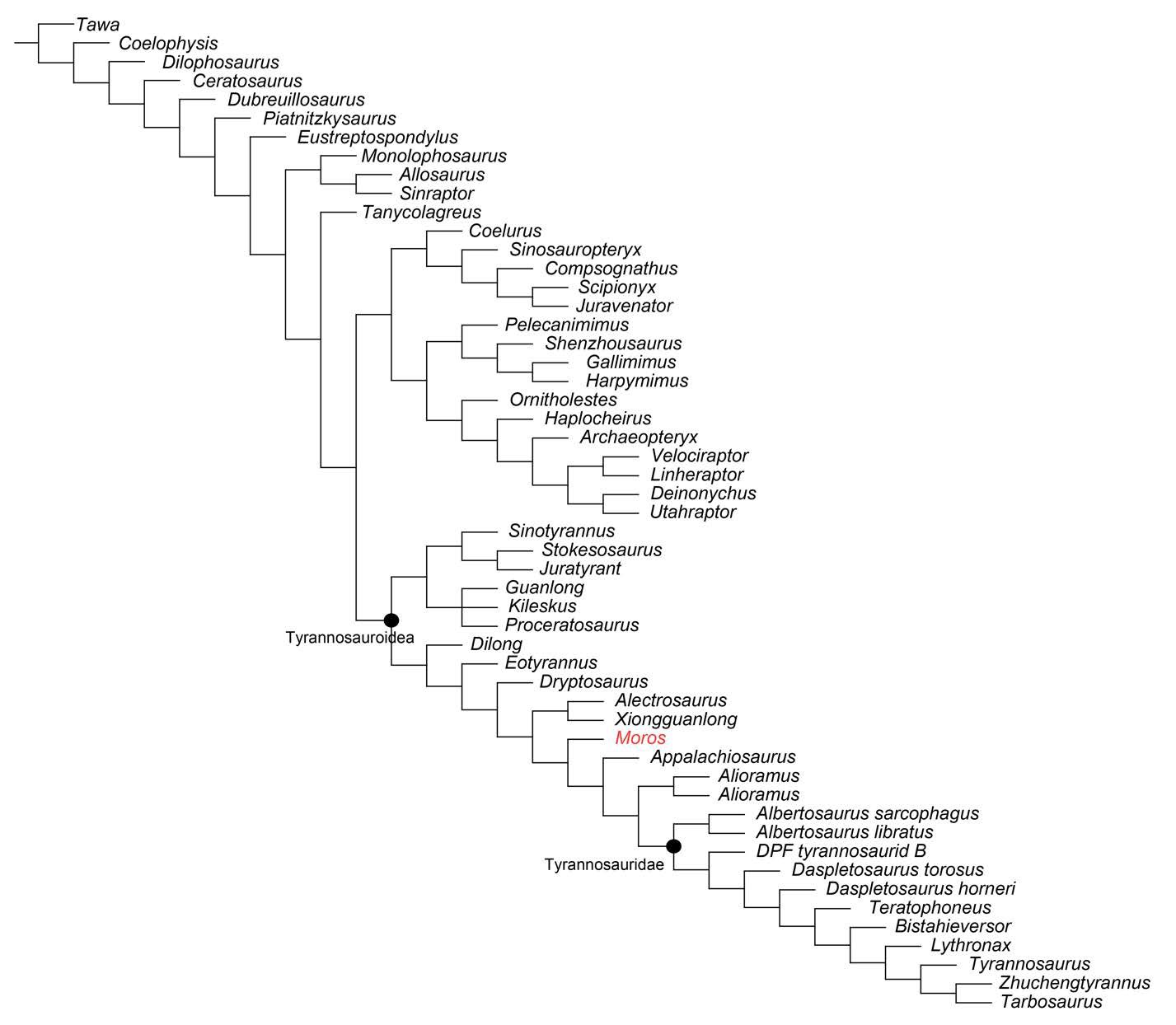


**Supplementary Figure 14: Strict consensus of two MPTs resulting from the inclusion of *M*. *intrepidus* (NCSM 33392) in the updated matrix of Loewen and colleagues47.** Results produce a nearly resolved Tyrannosauroidea positing *Moros* as an intermediate-grade tyrannosauroid closely related to *Xiongguanlong* and *Alectrosaurus*, and sister-taxon to a clade containing *Appalachiosaurus* plus all later diverging tyrannosauroids.

**Modified matrix file Brusatte and colleagues49 with *M*. *intrepidus*.**

xread 852 154

Allosaurus_fragilis

?10000?00000001000110010001012001110110010??0000000000001000000000010001

00000000000101010010000000100101000000000001000000000????000000000000000?10000

00000001000010010100000010001000001100000010000000000000000000100000000000?0?0

00?00110000100000010000111000210110000000002000000000?0000??0000000?00000??000

0000010000[0

1]0?0????????0000020?00?0000000101000?0?0010000?00000000000001000010?01000??0000

000?000?000002000000000000?00000?00?0010000000020000?000100??001100000[0

1]0?0000000100000000110000000000100011000000021121001001000100?1????00000000010

0??10?0000?00000000000011000?100?000?0?1000000000????000?0001001000010100000000

0000000102000000000000000000010000000000020101[0

1]00001000000000000000000100000010?0001000001020110000000001100000000100000000

0000001001000100000000000000000000000000000000000000000000000??000000000000000

000000?000001000000000000000?

Sinraptor_dongi

?10000?000?0001001100000001012000010110010?00?000000000010000?00000100?1

0?000000000101010010000000100101000000000001??????0??1?10?0?0??00??0??????000000

000?010??01001010000001?0?1000001100000010000000000000?000?000?00?000000?0?000?

001100001001000100000100002101???00000000000000000?0000??0000000?00000??0000000

010000???00?00??00?????2????????????1010???????????????????????????????????1??????0??

000??000000002000000000000000000?00?00??0???00020000?0001?0??0011000?????????000

10000000000000000001000001100000002102100000?010?00?10000000000000100??01?0000

?00000000000?11000?100?010?0?100?00?001000?0010000100100001010?0?00000000000112

000000?00????00000010000?00000020101??0??1000000????????????000010010?0000?00001

0201101010?00011000000??1?00000000000001001000??0???000000000100?00000000000000

???????00000000??000000000000000000000000?0010?00000?00000001

Dromaeosaurus_albertensis

?0??001000000000010??0???0?01??01110????1111????101?1001100??0000001001111

1000?0000101001?????????????????????????????????????????????????????????????????1?????

?????????????????????????????????????11?1?0????00??????00?0?00??1?????????1?00??01100

0100?0???????????????0???0000?00000??0000??0000000?00000??00???????????????????????

??????????????????????????????????????????????????????????00???????????????????????????

???????????0????0?????????010121?000??0???00?0?????????0???0??0??0???0??????0?0100?0

0?00????????????????????????0????00000100000???????0???????0001001?0?0????00??00????

?0?0?10???????????000?1000000010????00000000001120100000000?0?00010???????????????

???????????????????????????????????????????????????????????0??000?0?????????????0??0?0

10????????????????????????0200000????????????????????????????????????0?1?0??110???????

?????????000?0?

Deinonychus_antirrhopus

?0010????1???????1??0000?0111000111000111111100?1??00001110100000?0100111?

1000?0100101000?110001100?1121011????00110111121??????1?11011100100111000001002

21110101020220?01111211201?1111000000000100011010010000000001100000?00010?0010

002110000011110110000??00000122000000000?0????000????0???????????00000??00000000

0000?[0

1]0?0?????????????20000?000?000111000?0?0010000?0000000000000101?0???00000??01000

00?000?000000000000000000000000000?00100000000????????0?1????00111?????????000???

??00001?10?0010001?000001010??00?0???????00??0?0??0?????00001000000??01?00?0?0000

0001??????????????????????0?0?1001000?000?00?????????????????00000000001120100?0000

?0?????00000000?00001000100?????00000?1101010000100??1?0000?????????????????00000

?000000000?00?100?00??0001?1??0?000010?100000000010?010??00?0000000000000000000

00000??0????000000000100??1101?000110???200?10001?0

Velociraptor_mongoliensis

?001001001000012011200001011100011100012111?10?0101000011101000000010011

111000101001010000110001100111210111110001101111211110101111111100100111000001

002211101110202202011112212011111?000000000111011010010000000001100000000011?0

0101021100000111001100000000000112000000000000?000000?0000??0000000??0000??0000

?00000000[0

1]0?00??000?00000020000?000?000111000?0?001000???00000000000010100???00000??0100

000?0000000000000000000000000000000?0110000000010[1

2]21?0001101100011100000?10000000?0100001?10?0010001?0?00010101000000???0??0?01?

?00?0????000001000000??01?0000?00000001001?0?0???000??00110100?0???01000?000?000

001001000?00000000000??00?112010000???????00000??????????????01001000?00000111010

1?0?0?00?0100000110?01?0100000000000000000000000?000100000?000?101010?000010010

0000000010?01002000000000000000000000000000??0000100000000010000?1?10?00110100

20001000100

Balaur_bondoc

?????????????????????????????????????????????????????????????????????????????????

???????????????????1?2??1????0???10?????????0????111[0

1]11101001?1?00001???2???0111?202?02?0?1?22122????????????????2110001001000??00??1

???????????0?00??0??????????????????????1?????2?00?????????????????????????????????????

??????00000000?[0

1]???0????????????20??????0???0111000?0?001000???010?00?????010?00???21??0?00000020

?100??0000??????00???0?000000000?00??0?00000????????010????????1????????0000???????

????????????????????????????????????????????????????????????????????????????????????????

????????????????????????????????????????????????????????????????????????????????????????

???????0100?0?0??0?00?1101010000?00?01000?0110?01?????????????000?00??00?????00100

000?00??1??????0000100100?0000?????0?0???????????000000000000000000??000????00000

0210????????000?10???200?1??????

Tsaagan_mangas

?00100100100001201120000101010001?1000121111100010110000100??000000100?1

1110001010010100001100??1??????0?????????????????1????????11?111????????????????????

???????????????????????????????????????????????????00??00??00?0000???????????21100000

11?00?0000000?00001????0000000????000000?0000??0000000?00000???000???????????????

???????????20000???0?0?0??10??????????????????????????????????????????????????????????

??????????????????????????0????????0121?00???0??00011?0???????????00?0100001?10?0010

001?0?00010101000?00???0??0?01??00?0????000001000000??0??0000?00000001001?000???

000??001101?0?0???????????????0001001000000000000000??00?112010000000????000000??

?00?00001??????????????00?110???????????????????????????????????????????????000?000??

???????0?10?010?????10?????????????????02?0000000????????????????????????????????????

?000?1?1????110????0???000?0?

Bambiraptor_feinbergorum ?0010??001000012010[0

2]00?010111000?1100012111?1000101?00011?0?0?000?0100?1111000?010010100??1110?11

00?1??100?0?1100[0

1]1011??2????0101011011110?001?100000111?211?0101?202202?111022100???11100000000

010001101?000000000001100000000011?0010?021100??11?110??0000?000000122000?00000

?0??00000??0000??0000000?1?000??000000000100??0??0?0?0?0?0000020000?00000?011?00

0?0?0?1000??000000000?00010??????00000??0000000?000?000000000000000000000000?00?

011?100000000121?000110??00011101100?1000000010100001?10?0110001?00000100010000

00???0??0??1??00?0????000001000000??01?0000?00000001??1?0?????000??001101?????????

??????????0????????????????0?000??00?1120100?????????????0000000?000010001011000?00

0001110101000010?00100000110?01?010000000?00000?000000000?0001?000??00001?10?0?

100010?100????????????????0?000?????00??00????000000??00001000000000100?0???100001

10100200?10001??

Tianyuraptor_ostromi

??01?????????????????0????111?????????????0??1???????????????????????????????0?0

?0?1010??0???0?????????0?0?0?1??01?0112???????1??00?011110?001?1100001112?1??0?110

2223?21111022?0???1?110??????????00?11????000??0???1??00?0??01???1011?0???0??????1?

0??????????0????2?000?0??????????????????????????????????????0??????????0?00?00????000

00?0?00?000?0?0??????????????????????????????????????????????????00?1?0????10?????????

?????0?0????????20?101????????????1???????????0000?10?000??1?????????????????????????

??0????????????????????????????????????????????????????????????????????????????????????

??????????????????????????????0?????????????????????????????0??????????????????????????

?01?1?1???00??0?0????000???????????????0?????0?0???00????0????0?0???0???????0???0????

?0??0??????????????????0?????0???0?????????????????????????0010????????0?0???????????0

?0???

Sinornithosaurus_millenii 0001??????0??????????00???1110????1000111100?1????1??0????????00?00100??1????

010100??100????????1??1???????0???00?1??1???????01?1?11011110?0?????0000?00?201?01?

112023?2?111022?2?1???1?????????110001?1100?0?00010001??0000???01101111002000000

111100?00?00???000???20000?000?????0??000????0????00000??1000??00?00?0?????0?0??0?

??????00?0?[1

2]0000???0?000100?00?0??010?0??00????????????????????0000??0000?0000??0?0?00?????00

0??0?0000?00?0???0111?11000????????01??10000111?1100?100000?010100000000?0000000

?0?00000001000200???0??0??1??00?0????000000000000??00?0000?00000001??1????0??000?

?001101?????????0??????????????????????????001000?010112010000???????100?0??????????

????????????????0011101????00?000?????????0?01???????????????0?0???00000??0??00000?0

00?1??0?0?????10?????0?00?????0?0???0100??????0????00000????001?000?????0??000100?0

11?????0110????0???000???

Microraptor_zhaoianus

0??????????????????100?????????????????????????????????????????0??010?0?1????0?

01000000?????001?1??01?2100?01???0110?1212111?01?101111111010??111000011012111??

0112023?2?1110221201?11111??00?0001110111110?100??000?11?000????1010111100?0?0??

?1?110???0?00??0?00???20??0?000???????????????????????????0?0???????00001000?[0

1]0?00?0000?001000200???0?000?01???10?0???10100?000??1?0?000?????????000???????0?0

0?0000000000????000?00??000000000??1211111??00?021???011011???1?1?1100?100000????

??????????????????????????0????????????????????????????????????????????????????????????

?????????????????????????????????????????????????????????00???1?????????????????????00?

????????????00001????????01???1?10000?000???0000??0?01??10?????0?00000?0???00????0?

???0?00??0????1????0000???10??0?00???0?010???????0???0?00??000??0?000000?000?10?00

0000010??????????0?1??0?????1???1??

Graciliraptor_lujiatunensis

???????????????????????????????????????????????????????????????????????????????02

0???1??????????????????????????0???11?12????????????????01011?1100001????????????????

?????????????????????0??0?010?[0

1]11?1??0??0??00?????????????0???????????????????00???????????????200?????????????????

?????????????????????????????????????????????????????????????????????0??????10?0???000?

?0???????????10?0000??00?000??????????????????????????????????????????10??????????????

?????????????????000???????????????????????????????????????????????????????????????????

????????????????????????????????????????????????????????????????????????????????????????

????????????????????0????????????????????????????????1????00?00????????????????????????

????0000????00??????100??0??0???????0?????????00?0?00?????01??????????????????0???00?

???????????????0?000?10????????0?0?????????????????

Hesperonychus_elizabethae

?????????????????????????????????????????????????????????????????????????????????

????????????????????????????????????????????????????????????????????????21??010????????

?????221211?????????????????????1????????????1??????????0??1??????????????????????????

????????????????????????????????????????????????????????????????????????????????????????

????????????????????????????????????????????????????????????????????????????????????????

????????????????????0??????????????????????????????????????????????????????????????????

????????????????????????????????????????????????????????????????????????????????????????

????????????????????????????????????????????????????????????????????????????????????????

01000?0110?01?????????????????????????????????0???0?????1?????????????????????????????

???????????????????????000???????????????????????????????????????????????

Pyroraptor_olympius

?????????????????????????????????????????????????????????????????????????????????

????????????????????????0????????1????????????????????????001??????????????????????????

????????????????????????????01??1?????????0?????????????0??????0???????????????????????

????????????????????????????????????????????????????????????????????????????????????????

????????????????????????????????010??0?????????????????????????????????????????????????

???????0???????????????????????????????????????????????????????????????????????????????

????????????????????????????????????????????????????????????????????????????????????????

????????????????????????????????????????????????????????????????????0?0??????????0?????

????????????????????????????????????0???????????????0?????????????????0????????????????

?????????????????????????????????????????????????????????????????????????

Rahonavis_ostromi

?????????????????????????????????????????????????????????????????????????????????

?0???????????????011121???1?01?011112?12???????????0??11??011??????0?01111?10111120

?3?2012101?121??21110001000?011101101?0100????0??1?????0??10001011111????????????

????????????????????????????????????????????????????????????????0011000[0

1]0???????????????2????????????1100?????????????????????????010100???????????????00000

0?000000000100000000?00000000?001??01???0????????0?1?11?????1????????0?????????????

????????????????????????????????????????????????????????????????????????????????????????

????????????????????????????????????????????????????????????????????????????????????????

????100?1??0?0??0011??????0?????0?100000????01??10???0?0????00?0???00???????????00??

0?00?1????0000??0?0???????0???010???????????000?????????0000000?000?10??00?0001????

????00?0?11?0??0?????????

Buitreraptor_gonzalozorum

?0010???????????????00001011?00??????????100?10??01?0?????????00??001????????

??0210??100?0010111100111?110???100011012[0 1

2]121???????01101111010?1?1??0??????11?101??12[0

2]23??01?1?1?120???11?0??0??1?011?0?1110000?????0??1????????1101000110011000???0??

????0000??0?00?10200?00?0???????????0???????0?000101???0?????00000000000[0

1]0??????????01000100?0?0?00??0?10000?0?001100??00000100000001?100???????????????0

00????00???0?00??????????0?00?000?0010?00??????????????1????0010??1100?000???00?0??

??????1?11?00?0???010?0101?0??0????00000??1100????????0000???0?0??0??0??????????????

100?????000??00?0?????????????????????0????????????????0?000???0?1?2????????????????0

00???????0001001000??0??00000111010100???0?001??000???????01000???00??0?????????0?

0?000??????????00????0?1000100?0???????01000?????0?00010000000000????000000??0??01

000?0??001???????10????11100?00010001??

Neuquenraptor+Unenlagia

?????????????????????????????????????????????????????????????????????????????????

???????????????????11211111111???1????1????????????0??1001???????????01111?101111202

2020111?121202011?10000??0?010??111100000????0??11????0??110110?110???????????????

???????0?????220????????????????????????????????????????????0?00000?0?????????????????

1????????????1100?0?0?0011000?00000????????????0???????????????00000?00000000000000

00000?00000000?00?????????????????????????????????????????????????????????????????????

????????????????????????????????????????????????????????????????????????????????????????

??????????????????????????????????????????????????????????????????????????11011?0?????0

011???01???????00100000110?01?010000000?00?0100???00??????????000?00?00?1????10???

?01???????00???010?????????????00???0????000000??000010??0?000010?????????0??111001

000???????

Austroraptor

?0000???????????????????2?1?0????????12?0001????????????????????0?011??????????

?210??10?????0011?001????11???????????????????????????????00????????0????????????????

???????????????????1??????0?0?0?0??[1

2]1??????????0????0????????????????11?00??01??00?????????????1[1

2]20??0?0??????????????????????????????????????0??00?00????????????????????????????????

?????0?0?001100???00??0000000????????????????????????????????????????????????????????

?????????????????????????????????????????00?0??????????0?10100?0000010??1???????1????

???????????????????????????0??01?01????????????????????0?10?00????????????00000?000?0

0?????????????????0??0010000???????????????????000???????0001001101??????????????101

???????????????????????????????000????????00?00????????????0?????0?0??????????????????

?????????0?0001000?????0???????0000???????00??????0?????01?1????1?????1?0??0001??

Shanag_ashile

???????????????????1??1010110?????????????????????????????????00??011??????????

01001011???????????????????????????????????????????????????????????????????????????????

?????????????????????????????????????????????0????0????????????????21100001???00??????

?????????????00??0?????????????????????????????????????????????????????????????????????

????????????????????????????????????????????????????????????????????????????????????????

???????????????????????????????????????????????????????????1?10???00?0?000000?????????

????????????????????????????????????????????????????????????????????????????????????????

??????????????????0??00??00????????????????????10??????????????????????????????????????

?????????????????????????????????????????0?0????????????????????0??????????????????????

???????00????????????????????????????????????????????1?????????????????00????

Mahakala_omnogovae

???????????????101??????????????????????1?00???????0?0011??????????????????????

02????1???00??0?11?011?211??1?100011012210????????????????00??1110000?100?211101?2

??????????????????0111100????0?011001101?00000??00??11?0?????0000?????00????????0??

??????????0????1[0 1]2?0??????0?????????????????0??1?00???????????0000010000[0

1]0????????????????????????????10??[0

1]0???0?0000???0?????????????100????0??0???000000??0??001??0???000000000?00000000?

001??00?000????1???0??????011?0????????00000???????????????????0???0?????????????????

??????????????????????????????????????????????????0?????000?00????????0???????????????0

0010?????????????????????0????????????????????0000?????0?01?000???????000??????????00

????00100000???????0100000?0?000?100?0?000?0????0?0?0???0????1?10???00???????????0?

???010?20????0?0??0???????000000000??0??0100000000010?0????1?000???10????01????0?

Atrociraptor_marshalli ???????????????????[0

2]00?0??1010????????????????????????????????00??010????????0?0000101001??????????????

????????????????????????????????????????????????????????????????????????????????????????

????????????????????0????0????????????????21?00??0??111?????????1???????????0?????????

?????????????????????0????0????????????????????????????????????????????????????????????

????????????????????????????????????????????????????????????????????????????????????????

???????????????????????????00001?1????1???0?0?000?01??????????????????????????????????

?????????????????????????????????????????????????????????????????????????????????0?????

0?00????????????????????0??????????????????????????????????????????????????????????????

?????????????????0?0?????????????0??????0????????????????????????????000???????????????

?????????????????????????0??1??????????????????00????

Utahraptor ???????????????????[0

2]00?0??????????0?001??????????????????????????????????????0????0101??1????0?1100?1??

1????????0??011?????????????101?1???????????0?????????0?01????????????11?????0111?00?

?0?001000100???10????????11??0???????0?????0???????????1???0???????0???10???0???0????

????????????????????????????????????00000???????????????????20000?000000??????????????

?????????????????????????????????????00???????0??0000000000000?0000?????0?10?????????

????????????????????????????00?????000001??????????????????????????1???0??00010?0?????

????????????????????????????????????????????????????????????????????????????????????????

????????????????????????????????????????????????????????????????????????????????????????

???????????01?????????0?0?????????????0?????0????????????????00????????????0??????????

?????????????????????0?0???????????????????1????????????????

Adasaurus_mongoliensis

?0010?????0????2??????????????0?????0?1??11?1?0?101??001??0?????0????0?1???0??

???????????01100?11?0111?10?111[0

1]100??011?1?1??????1?111111??????????????1022111010102?2202?1?11221001?111000000

?000?11010010010??00??00110?0?0?0001??00100?????????11????????00?0????1??????????0?

????????00??00???000?0???????????000?0010000[0

1]0???????????????200?0???00??01110??????????????????????????????????????????????00?00

0000000000000000000000000?002??11?00???00???21?000??0??0001?1????????????0??0?????

????????????????????????????0?????????1??00??????0000010??000??0??00?0?0?00????????0?

?????????0????????????????????????0??????????????????0???????11201?????????????????????

???????????????????????????????????????100000??0?01???????????????0?0???00?0????????00

0?00????10?????????????????????????00??????0??00???????????000000??000010000??00010?

?????????01????????????????

Achillobator_giganticus

?????????????????????????01?1??????????????????????????????????????????????????0

000101??????0?01100?11210???????0??011?11??????????101????????????00??10220??110101

02102?011011?001?21110?0????00?000?101????0???????1???0????001??00100021100??0??1

00????????0??????????????????????????????????????????????????????0000??????????????????

??20000?0?0?0?0??10??????????????????????????????????????????????00???0?00000??000???

????0?0000????????0???????????????0??????????0??????????00???????????00?001???0?0?000

??1????????????????????????????????????????????????????????????????????????????????????

????????????????????????????????????????????????????????????10??????????????0100???????

???????????????????100000??0101?0100000?1000??????????0?????????0??01?????1??0??0???

???????????01??0?????????0??00?????????0000000???0000100???????1???????????????1?1???

??0?00??

Saurornitholestes_langsto

?????????????????????????????????????????111????????????11????0?????????????????

100101?00?11000110011121011011100?1011?1?1????????????111?????????000111221?1?1?1

?2?0?????????????????11??0????00??001101??00???00?0?1??00?0000010?001000????????111

0011??????0????1220???????0????????????????????0000???????????0???????????????????????

?????????0???00??1????????????????????????????????????????????????0????????????????????

?????????????????0???00??????????0??????00111????????0?00???????????00?01??0?0?0?000?

00????????????????????????????????????????????????????????????????????000??00?1???0?00

0??????????????????????????????0??0000000????????????????????0???????????????1?0??????

?????????????????????????????????????????????????????????0??????0????????????????0?????

??????????1??01????????????0?????????00???????????????????????????11??????????????????

0????

Saurornithoides_mongolien

?00??1?1??1101???0?110001?1000????????2?????????????????1?010?100?0010??1????

0001110101?????????1???0??1?????100??1??????????????????????????????????????????????0

2012020?010??10[0

1]1?11110???????????0???10?01??????0??1?0???????????010??11000??0?1?01?0?????0?000??

????0??0000????0?1000?0??0???????????0000???0????00100?0?????????????????????????????

???????????????????????????????????????????????????00????????0?0????00000000000000?20

?02100000000000201001?10???1110?1???????????00?0100000??2??11?0?0?1?0100010100??0

0???0??0?????0??0?????????????00????????????????????????????????????????0???????1?????

???000????????????????0?????????????????0???????00000??????????????0????????0?0???????

??????????????????????1?010???????????00????00000????????00??00????0?0???00??????????

?1??????0??000?0?????0???????????????????0?00???????0?101?0????????1???0??????0?0?00

Zanabazar_junior

?00101?12?110100?001?000??100000????2022000?21??0??11100??????100?001???1??

??000111010100??????????????????1?1000?1020?1????????????????????????????????????????

??2?????????????????????????????011????2???????????00??00???11????????0??110000000100

000????1??00???????00?000?????001??0?00?????????????0000??0?00????????0[0

1]0?????????????????????????????????????????????????????????????????????????????????????

??????????00010000??000??2????1??0???00000201001?11???1110?1???????????00??100000?

?2??11?0?0?1?0100010100?000???0??0?????0??0?????????10??000??00?0???????????????????

???000?00011010??????????????????000?00000????0000???000?001???????????0????00000??

?????????????????0??????????????????????????????????0?????????????0?0????????000???????

??????0?????00???0?????????????????01?00?0000????????????????????????????????0?00????1

00100???????????1????000?0?

Xixiasaurus

???????????????????01000101110??????2?22????????????????????0?00??001????????0

00201??01?0?????????????????????????????????????????????????????????00?1??????????????

??????????????????????????????????????????0???00?????????????????????1?000000???01?000

0????00???????000000???00????????????????????????0????????????????????????????????????

?????????????????????????????????????????????????0??????0??????????????????????????????

??????????????0?0????????????????????????????????00????110100??2?01100?0?1?010001010

00000???0??0?????????????????????????????????????????????????????000??0???????????????

?????????????????????????????010?000????????????????00000?????????????????????????????

??????????????????????????????????????????????????000??????0??????0????0?0?????????????

?0????????????0100???????????????0????????????????????????1?010??????1??????????000???

Byronosaurus_jaffei

?????101???101?1100110001011?0??????20220??????????1?100??????0000001??11???

?000211??01?0?0???????010121????????0??02????????????????????????????????????????????

????????????????????1????0?0??0???????21??????????0????0????1???????????11000000?1?00

????0??0?000??0???000000????????????0??????????????0?0????0????0010??????????????????

???????????????????????????????????????????????????????????????????????????????000?????

????????????????????????000200011??0????????1???????????00??100000??2?011?0?0?1?0100

010100??00???0??0?????0??0?????????????00????????????????????????????0???????????0????

??????????????00?000000????????0??00??00?????????0???????0?0000??????????1???????????

????????????????????????????????????????????????????????000?????????????0????000???????

?????????????????00?0100?????????????????????????????00?????????1?0?0?1????1??????????

000?0?

Sinornithoides_youngi

?0??01?????????????1?000??1??0????00???2???????1??????????????00??0010???????0

001110?01??????11??001???????????0011?102121011???1?00?1?101?0???100000????21??01?

?02??30???11?0??001??1110?????0???1?000110001?0??0?0?1??00?0???0???0010?0??00000??

??0??0??0???00???0?2000000?0????????????????????00?????1?00??000??????????[0

1]0?0????????0??0?20??0?00000?0??1000???0?1000???00?0?????????0??010?0000???000000?

?00??0??000????00000000000000020?02100000000????????101??????1?010100??1?000?????

????????????????????????????????0??????????????????????????????????????????????????????

??????000??0???????????????????????????????????????????????????????????????????0?0?00??

???????0???????????????00?110101?000?000???????????????1??????0?????000???00?0???0???

0?00??0??1????0?????100????0?0????????0???000?000???00??10?000?????0??00???00000000

010????0?1?0?0101????0????0????

Mei_long

?0????????0????????11010?????0?11?002012100001?1010??100??????00010010??1?00

00?021???01?0011011100011?2110?01???0110222111?1?????111?11100?0?1?1000001?01211

1?1??1200302?1110221?[0

1]?0111100?0101001000??110011?0?0000???00000??000100?1??0?1?000000?001?0?0000?00

0001020000?000?????????00?0??0??00??0101????????0?0000010000[0

1]0?0????????0100020?00?00000?0111000?0?0000000?0000000?000?01?1?0???00000????000

00?00000010000??00000000000?000021?01100000??0???20???1010??101100?1100?10?000?1

10??0??0??????????0?1?01????0?????00???????????????0?????????????00??????????????????1

???0??????0????0????0????????????????????????????????????0?000?????????????0???????0?0

000?????????0?????????????????????1?1?0????????????????????????????0??????00???00?00?0

??????0????0????1?0?000?1001??00?0001?0?010???0???0?0000000000?000????00???0???00?

000000100?????????01??????????0?0???

Jinfengopteryx_elegans

?0?????????????????1?01???2000????????1??00?0????1???100??????00?00??0??1??0?0

00?1???0???1??????????????????????02????2?200???????1101110??????100000?????1???????

???????????2??0??????????0?????????????????0?0??00????00??????????????01?000001???0??

0?0??????0????200?0?000???????????????????????????0??????0?000???0?0??0?0????????????

??00?0???0?0?01???0????????????????????????????????????????????????????????????????????

??????????????10?0000???????????0??11?01????????????000?110?????0?01??1????0?????????

0?????00?????????????????????????????0??????????????????????????????0????0????0????????

????????????????????????????0??00??????????????????????????0?????????????0????????0????

1??0????00????0??????????????????????0???????????????0?0??00??????00????1?0???0???????

?0????????????????000??????????????0?0???????00?????????001?0?????????????????????000?

??

Anchiornis_huxleyi

000100?????????????11010??11100?????0?2?000??1???11???????????000??01??1?????

00020???01??0?101???00?0??010?01???01101220??01?????011111110000??100000010?21?10

011020?202?1?1122121???1?1?000??1?01?000121100010?1000?1??00?0???????0?11?0110000

01???0??00?????000???030000?000??????????0??????0????0??????0??????00000?0000[0

1]0?0????????0000020000?0?00??0?10000?????10?0???0????????????????10?01000??0?00?00

??00?00000????????????00000?0?00??0100000000????????00??1110?1?1?1100010?000?110??

0??0?01?010?0?0?1??101?00??0??00???????????????0?????????1???000??0???2??????????1??

???????00????0????0????????????????????????????????????0?000??01?????????0???????0?000

0?????????0???000??????000??????1???00??0000100000??0?01?????????0?????0?0???00?00?0

??100?00??00???11?0?0000???????0??????????0????1000????0????0??0?0000000???00?0????

???00100???0???0?01??????????000???

Xiaotingia

?00?????????????????1?1???111000????0?2??????????11????????????001001??1???00?

?020???01??0??0???1?0?????10?01?????1??????10??????01?01111?10???1000001011?1110?1

102122?????102?12[0

1]??????????10????????1?010001010000?1??0?10??00???0010?011000??1???00??0?????000??

?22000??000?????????????????????0000?????????0??000?10000???0????????010002?????????

???11?000?0?00???0???0?0?0????0???????????0100??1?00000???0?0000???????????????????0

??0?02????10000????????00??1??????1?1100?10?000?110??0??0?01??1????0?????0???0?????

0????????????????0?????????1???00?????????????????????????????0????0????0??????????????

??????????????????????0??00???1?????????0???????????00?????????0???????????????0011???

???0???000010?000???????????????0???????0???00000?0??100?00??00?1??1?0???????0????0?

?????????0?????000?????0???0??000?000?????0??????????0?100???0?????0101????0???000??

?

Aurornis

?0010??????????????0101???11100???000?1?0??????????????????????00?001????????0

?020????1??0????????0????????0????0??0?221?00??????01?01111??0???10000??001?011????

02122020??10???????????????????????0??100000?000000?1??0??0??00????111?011?0000????

0???000???0000???20000?000???????????????????????????0??????000??0???000?0??????????

00???2??0????????0?0?010????????0????????????????????10?0??00??0?00?00?0000000??????

??????????0???0??????1??010000????????0???1?1?1??1??1000100000?110??0??0?01??10???0

?1??101?001???000???????????????0?????????10??000??0??02???????00???????????00????0??

???????????????????????????????????????0???0???1?????????0??????????00?????????????????

??????0???01???1???00??0000100000???????????????0???????0???00??0?0??100?00??00????1

?0???0????????0??????????0?????00??????0??????0?000000???????????????00100???????????

0?????????00????

Eosinopteryx

00010??????????????1?01??????000????0?1?0?????0??1?????????????00?001????????0

0020????1??0????????0?????????????0??0?231?10???????1101111??0???100000?001?1?1??1?

02002?20??10???????????????????????00?1??0001000000?1??0??0??00????011?0???00??????

00?0?00???0000???20000?0000??????????????????????????0??????0000?0?10000?0?0????????

00???2??0????0???0?1?010????????0???????????????????????0??00??0?00?00?000?000??????

??????????0?????00??01???000??????????0???11??1??1??1??0??0000?11???0??0?????????????

???????0?????00???????????????0?????????10??0??????????????????????????????0????0?????

????????????????????????????????????0???0???1?????????0???????????0??????????????000???

???0??0011??1???0???000?100000???????????????0?????0?0???00??0?0??100??0??0??1????0?

0?0????????0??????????0????100??????0??????00000000???????????????00100?????????0?01?

???0????0????

Troodon_formosus

???1?1112?1101000001???0?011?0??????20220000210?0??01100????0?10??001??????

????0111010100???1111100101211111?1000?1020??11?????????????1?010?????000010??????

????0?0?2?20?11001?0??01111000???00010000021??01????000??1?0??010???1??0??0?1?000

00?0?0???????00??????????????????????001??0?000?????0??1?1?????????00????????????????

????????????????????????????????????????????????????????????????????????????????????????

???????????????????????0?????00000201001??0???1110?1????????0000?????????????????????

?????????????????????????????????????????????????????????????????????0?????0000??00110

????????????????????0000001000??000000??0000000????????????????????00???????0?0??00?

????????????????????0?????????????????????????????????00000?000??0????????00??0???0??0

????00??01??0000?0?1??01?01?00?0????????????0?0?????????????????0?000?10?0?10?0?0?0?

???????????0??0?

Sinovenator_changii

?0???0002?000011110010101?1110?011?02??2??0011???1?10100????0?000?001??????

0000011110?1?????11?10100012110?001000110222?1?????????110111???????100?0???0?211

?010112[0

2]0302?1110221201?11110000??10011000111??110?000000???0000110001000110011000??1

??011??0000?00000??0??00000000000?000000?00?0??0000010100[1

2]00??00000?0010000?????????????????20000??00?0101?10???????????????????????????????

???????????????00?1000000100000000000000?0?00?020?0110?00000000020?010?10??10110

00???????00000?????0000?02?011??00?1?0100010?????0??????????????????????????????????

????????????????????0????????0?00????1???????????????????0????????0???00000??00???0???

?????????????????00?????????????00????0??????00111?????????????0000?0??0?01???????1??

0????0?0?0???0?0???????????0?0?1?1??0?????100????????0????0??01?01?0?0000?00??????0?

00??0???0000?0000000001?1???0?????0??1??1?????000?0?

EK_troodontid_IGM_100/44

?????0012???????????????????????????????????????????0???????????????1??11???????

???1??????0????????????????????????????????????????????????????1?00000?????????????????

??????????????????????????????01?1?001???00??????0?0??1?????????0?????????????????????

????????????????????????????????????????1?1????????????????????????????????????????????

????????????????????????????????????????????????????????????????????????????????????????

??????????????????????????????????0????????000?????????????????????????????????????????

??????????????????????????????????????????????????0????????????????????????????????????

????????????????????????????????????????????????????????????????????????????????????????

?????????????????????????????????????????????????????????????????????????????0?????????

?1????0????????????0000????????????????????0?1?????????????????????????????

IGM_100/1126 ?0????002?0000?11[1

2]01?010??2000?011?020121000110?01010100??0??000000000?11?000000211??00?0????????

??????????0????01102???????????????????????0??1000001???211?010?020020200010131012?

?111000????0??2?001210001?00000011?00?0?020000?00100?1?00000111?01?0?00?000003??

????000000?????00??00?00?0??0??0000?00?00???0?0?????????0???????????????????????????

?????????????????????????????????0?00???0000???0100000?00??0???0?00?00?????00?1?000?

20?02????0000001020?111??0??101?001???????0000??????????????????????????????????????

????????????????????????????????????????????????????????????????????????????????????????

????????????????????????????????????????????????????????????????????????????????????????

????????????????????????????????????????????????????????????????????????????????????????

???????????????????????????????????????????????????????????????????????????? IGM_100/1323

?00???????0???11???1?000??20000?1??020121?00?1??0?0??????00?0?000?0????1??00

000021???0000???????????????????????011???????????????????????????????????00??1??????

020?20200[1

2]102?1011???????00???0????0??2???01??00??0????0?0???00?0?00100?1?000000?1?01?0?00?

00?003??????0000000????00?000?00?0?????0000?0?000??0?00?????????????????????????????

????????????????????????????????????????????????????????????00?????00?00?0?00?????????

1000??2????????????????????????????01??????????????????????????????????????????????????

????????????????????????????????????????????????????????????????????????????????????????

????????????????????????????????????????????????????????????????????????????????????????

????????????????????????????????????????????????????????????????????????????????????????

???????????????????????????????????????????????????????????????????????????????

Archaeopteryx_lithographi 100?0000??000??112010010??1110?011000012100?10?0000??100111?0?00000100000

2?00000200??00100?1?1???00?0??1?0?0???0021012311000????1011111111000?11000000011

2111010?12003020121022?2?2?111100000?0001000000030?0000000001100000??00?0001100

11100001001?01000000???0000?030000?0000000?00110??00?0??000?00??000???00?0000000

00[0

1]000?0????????00000?0000?000000010000000?0011000?000??000?0000100000??00000?000

000000000?0?00000000?00?0?[0

1]1000000?00???00000100001020?00001011101?0101100?1000000110?00000?01?01?0000?0

?0100?10100?000???0000?01??00?0????00???10??000??????????000000?0?0????????000??00

11000?????0??????????000000011??????00000?000000000??00???0???0???0?0100???????000

?0000????????0?0011101?10000?000?100000??0?01?01??????0????00?0???00?00?00?100000?

000?1?1000?0?0010010??000000?0?010020?1000?000000??0?000000?0000?000?????0?00001

00001001???0111????00?100010?

Confuciusornis_sanctus

10010??????????????1?000?00??0001???0??2??0??0??00??01???????000010000?10?00

01?1?????????0??????????1021?0?2???0?2????4??111?11010??13111000?11110000001121?1?

1??12000?2?111023?2??12??10??11??120211010030?0000000001102000??00?0101??11?1000

11001??????0?????000?123001[0 1]00120?????????10??00??011200??01[1

2]0111110000001100001001000120000?00?0000?0??0??010000000?000211??0010000000010

102101011[0 1]?10?0000001010000000001010100100011000011000?0000000[0

1]000???2000001101110?00??1100?10?000?110?00000??1?00?0000?0?01?0?10100?00???????

???1??00?0????0000000??000??01?02???00100000?0????????000??00???0??????????????????

??????????????????00010???0?0??00??10????????????0???????0001?000???0????0?00111?0?1

?000?100??????0??0??1?????????0????00?0???00?00?0??100000??0??1??0?????0?????0??00??

?????010???????0?????0?0000?0?0??????0??0??????0?0000100?0?0?????01???????????1????

Jeholornis_prima

10????0????????1???0???0?????001??????????????????0???????????0000?1???00?00?1

?1?????????0????????0????1?0?11???02111031??01?0???011031110001??1000001011?1??0??

01?0???2???1?221?10???????01????0101101003000000000??0??1000??00?010????1???000?1?

????0???0??0??0??12300?10010??????????????????0????00?00000??00?000000100000?0??00

1???0000?00000?0?1000120001000?0010000?000?0000?00001000010110000?000000000000?

00000?010000000001000001000?00000001100????????011?1110???1?1100?10?000?110?0000

0??????????0?0???????0??????????????????????0??????????0??00???????????????????????????

??000??00????????????????????????????????????????0??00???0????????????????????????????

??0?0??0000???????0?0011101?1?0???000???0000??0?01?????????0????00?0???00?0??0??100

000?00??1?10???0?00???????00???????010???????0?????0???0??0?000?00???000?????0?0000

100???0?????0??????????1?1?1??

Jixiangornis_orientalis

100?????????????????0010?????001????0?0??00000??????0100??????000001????0?0??

1??0????????00?0???1?00????1??20???02??1031??11?1?0001103111000110100000111121010

10212000020101?221?10?2?11?001??1?01[1

2]1101003000?00?000?0??1000??00?0100??111?001?1?????????00?000000?12300?00010????

??????????????????0?????000??00?0000?11?0?10?11?0?????0000000000?0010011210010?0?0

000000?000???0??0????0??010?00100??0000?001000000?00??0000??00000100000?00?0000?

001100????????00??11????01?1100?10?000?11??00000??????????0?0???????0???????????????

???????0??????????0??0???????????????????????????????????0?????????????????????????????

????????????00010???0??????????????????????0???????????????????????????????0?1?00??00?

??????????????????????0?????0?0???00?0??0??100?00??0?????0??????????????00?????????0?

????????????????0??0?0????????????????????00100???0?????0????????????1????

Yanornis_martini

?00????????????????1?010?????0?11???0??2??0??????00???????????0000011???0????0

00?1???00??0????1???0????????4?1???????04??????110?011031110?011?130010????????????

????????????23?010?2??10??1??0?0121[2

3]0000300000??0001???00?0???????0???21?1?0??????????????????0?????300100012?????????

?10???????1?????????????????0?0011?00???0210?[1

2]??10100001010?1?11?112100111??0101?0101?10000?0?????021[0 1]101311[0 1]1100[0

1]100?0?1?0??0?00??????0??00?1110[0

1]?1?00??1?0??01100????????01??1????????1100?11?001?11??????0??????????0?0???????0??

???0????????????????0??????????0??0????????????????????????????????????????????????????

?????????????????????????0???0???0??????????????????????0???????0?0??0?0??????????0011

1?0?1?00??100???0000??0?01?????????0?????0?0???00?0??0??100000??00?1??0?????????????

?00?????????0?????000?????0????????000?00???????????????00100?????????0??1?????????0?

1??

Apsaravis_ukhaana

???????????????????????0??????????????????????????????????????0002?1?0??0???????

?????????1??11??10200??1???5?100021??24??????1?010110311000011113003??000?1???1?20

1000?2?021?23?03?12?010?????1??12130100?00000??20???1?20?0?????0?00??21???????????

?????????????????300?10?????????????????????0?1[0 1]?????1?0????????20010?[0

1]001??21??1????????00000?0?1000121011111?01011010[1 2]0111110000101021110?[2

3]120100?11??20?1[0

1]0010?11?110?1012112111111?0000?1?0??01101????????011??????0?1????????001????????

????????????????????????????????????????????????????????????????????????????????????????

?????????????????????????????????????????????????????????????????????????????????0?????

????01???0?????0??0?0011100010001?1?0?1?0000????01?010??????????0000??000??????????

??0??0??1????????00?00100?????0??????0???????0????0000000????00000????000????0?000?

10????????0?0?11????0?????????

Yixianornis

100?????2?021??1?????0?01????0?1?1?0????????????????0100??????000?010???0?00?

00120???10?01???1???0000??1?0?40??0???0024???11?1100011031110?0010130000100021?1

01?212003020001023?23?0211?0??1??????2130100100000??000?0??1000?20??0?000001?????

??????0??0??0???0??0?1230010001???????01??1????????11[0

1]?????1?0?????10?20011000?11021??[1

2]??10100001010?11110112100111100?0110???01?000???0?01021[0 1]10?31201100[0

1]100?00100?00?002?101??????11110[0

1]?1?00??1?0?001100??????00011?11?01????0100?10?000??1??????????????????????????????

???????????????????????????????0?????????????????????????????????0?????????????????????

?????????????????????????????????0??????????????????????0?????????????00?0????????0011

1?0???000?100?1??000????01?????????0?????0?0???00????0??100?00??0??1????????0???0???

?00?????0???0???????0?????0???0????000?00????00??????0??00100?????????0?11????0??1??

????

Sapeornis

?001???????????????0?010??11000????00??21?0?00????0??????0????0000010??10?000

00020??????00????1???000????0?211??0210004??100?????21101111010011100000101121??0

0?212000?201[0

1]00221210????1??01??1001210?10030000000000????2000??0??0100??0111?000???1???00??

0???0000??23000000000??????????????????????????0?00???0000000010000100????????0000

020000?00000?010001000?0?01010?0010000?0???010?1[0 1]10?11100??0[0

1]00?0000?0000?00?000??00?00??000001?00??0?00001000????????001?1?1011?1?110001000

00??1?000000??????????0?0???????010??00?????????01??00?0?????????00??0??????????0???

??000??0????????000??00????????????????????????????????????????00000???0?????0???0???

????0?0100???????000??000?0??????0?00111?1?1?000?000???0000??0?01?0???????0?????0?0

???00?0??0??100000?000?1?10?0?0?0??0?1???000000?0???0????1??0???0?0??????0?000?000

??000?????000000100?0?0?1???00?1?????????1?1??

Neuquenornis_volans

???????12????????20?????????????????????????00?????00???????????????????????????

???????????????????00??????????????????????1?11011?1031110?01111000??????????????????

?????????????????010???????????[0

1]010030??0????0??????0?00???????????1???????????????????0???????0200?????????????????

????????????????????????????0110???????21?????10110??0100?001?001?0??[1

2]??1???01?0???0?????[0

1]?????????2???20?1??01????????????????10?1????????1?0???1011???????0?000???20000011

0?????????????????0??0?????????????????????????????????????????????????????????????????

?????????????????????????????????00???0???????????????????????000??0100???????????????

???????????????????????????????????????????????????0111?????0??????????????????????????

????????0?0???00???????????00??0??1???0??????????????????????????2???????????????0????

?????????????????????0010?0????????0?????????????????

Patagopteryx_deferrariisi

?0????????????????????????????????????????0010??????????????????????????????????

??????????????????2????????????????????????????????????????0?1???????????211101???????

??????????????2?010001??100121301001?000???????1????????00?0?????1??????????????????

????0?????0??????????????00?1??????00??111110000?????????010000000????????0????????0

00????0101?1210[0 1]00???[0 1]?10?0???01000001[0 1]0001?[1

2]00???30???????00?2000000?010120????0?000[1 2]11001100000?[0

1]?0??0?100?1?200??01?0???0?001????????0???0?1???????????????????????????????????????

?????????????????????????????????????????????????0??????0?0??0?110????????????????????0

??????????????????????????0020000??00??????????0?0?00??0001?00000????????00111?00100

???????1?0000???????0100??0?0???0?000???00????0??????00??0??1??????0?10???????????00

???010???????00000000??0?????000000???00?1???00000010??????1???0????0???0?1???10?

Cathayornis

?0?????????????????1?010?????0?1??????????0???????????????????00?????????????00

001???????0????????[0

2]????????3?????2????4???0??11?02?1031110?011?1300?0?01111?0?00?12000?201?1022?23?

????????1???0??[1 2]1[0

1]1?0?3000?0???00????0??1??0??0100???111?????????0??0???????0????2300[0

1]0?012???????????????????????????0??????0????0111??0010?20??1??10110?101????010?012

00010010110[0 1]?01020111110?[0 1]?1?????210120?11?01?0101011?0?000001???1?0?0?1[0

1]1?0???????????0??01110????????01????????01?????????0???110?00000??????????0?0?0100

?10100?000???????????????0??????????0??00?????????????????????????????000??00?0?0????

????????????????????????????????0??00??00?????????????????0?0100???????0??????????????

???00111?0???0?????????0000???????????????0?????0?0???00?0??0??????00??00?1?1??0????

???????????????????0????100??????0???0?????00?00???????????????00100?????????0????????

???000???

Concornis

?????????????????????????????????????????????????????????????????????????????????

???????????????????00????????????????????0??11?02?1031110?0?1???0030????????????120?1

02??11?23??3?0???1???1???0?1[0

2]11??0?3000?0???0????????????????001??1???????????????????????????2300???????????????

??????????????????????????????0111????10?20??1?0101100101?0?0010001?0??[0

1]001?110??0102?1???10???1[0 1]1?[1 2]??????????????[0

1]????????????00?????0?100??1?00??1?[0

1]1??0??????110?????????????????????1100110?01????????????????????????????????????????

????????????????????????????????????????????????????????????????????????????????????????

????????????????????????????????????????????????????????????????0??????????00111?0?1?0

????????????????????????????0?????0?0???00???????100?00??0??1?????????????????????????

???0?????????????0????????0?????????????????0??0010??????????0?????????????????

Gobipteryx

?0?????????????????1?01010?0?0?????????????????????????????11?000?0??????????1?

1?????????????????????????????????????????????????2????????????????????????????????????

????????????????????????????11???????????????????????????????????1????0????????????????

??0????????1100100?00110?10?????00?????0???0??0???1?????????????10??????????11??1000

0?0?100012000[1 2]001?1?0110???0????????????1?121??[0 1

2]0?????1?0??1????????000?0???10?001111000?1?11?0????????????????????????????????????

???????????00000??????????0?1?01????0???0?0???????????????????????????????????????????

?????????????????????????????0????????????????0??????????????????0?01000?000??00???000

????????????????????????????????????????????????????????????????????????????????????????

???0????????????????????????????????????????????????0??????????????????????????????????

?????????0???0??????????????????0????

Vorona

?????????????????????????????????????????????????????????????????????????????????

????????????????????????????????????????????????????????????????????????????????????????

??????????12?010011101001112110???00????????????????????0?????2??????????????????????

????????????????????????????????????????????????????????????????????????????????????????

???????????????????????????????????????????????????????????????????????1010111100001?0

00000?????????????????????????????????????0????????????????????????????????????????????

????????????????????????????????????????????????????????????????????????????????????????

????????????????????????????????????????????????????????????????????????????????????????

?????????????????1???????00000000?00??????????????????????????????????????????0?10001

????????????????????????????????????110?00000010??????????0????0?????1???1??

Songlingornis

?????????????????????0?0??????????????????????????????????????00?????????????000

?0????0??????????????????????????????????????11??[0

1]???3?????????????????????????????????????????????????????????????????0????0?????0????

?00?????????????????????????????0???????0?????????0?01?????????????????????????????????

??0???????????????21??[1

2]?000100?010???1?11011???????????????????????????????????????????????????????????????

?????????????????????????0??0?????????????1??1????????????????????????????0????????????

????????????????????????????????????????????????????????????????????????????????????????

???????????????????????????????????0??00??00?????????????????0?0?0?????????????????????

???????1?1?????0????????????????????????????????????????????????????????????1??????????

????????????????????????00????????????????????????????????????0?1??????????????????????

??0????

Pengornis_houi

?0010??????????????10010??0?00??????0?12??0??????0?????0??????000?001????????0

?0?00??00???01??????20???0???2??????????4????1?????2?1031111?0??????03??001?1???????

???????????2??2??????????1??????211???0?000??0??000???0??0??00???0???11?1?000???????

???00????00001230000?000?????????????????????????????0?????0???0011??00100????????01

[0 1]00?00??????0??1??012111[0 1]0?011010001[0 1][0 1]1?????1??1???10[1 2][1

2]0??1??1?11??????0????00?0????0??0[0

1]?1?00??1?11??0?0??0?100????????01?????00?01?1001011??1??110?00000????0??0??0?0?01

00??0?????00???????????????0??????????0??000??????2?????????????????????0?0??0????????

?????????????????????????????????0?0?0???0?0??00???0???????0?0100??0?0??0??1?0???????

?????00111?0?1000????0???0000???????????????0?????0?0???00?0??0??????00??00?1??0?0??

?0????1??????????????0????1?00?????????0??????0?0?????00?1???????00100??????????1?1??

??????000???

Hesperornis

?0?????????????????00010100?00??????00021?001????00???00????1?000?001???1?00

01?021???00??10101110?2100??11?50100?210?24??11??10??00103111??????????3??0202100

?1??01000???021023?03?12???0001??11?1213110010??0?00??01?1?0000?000???00??2??1?11

11????0???????10000??10?1?10?1121200110?1010??1110011100??00?0???101012101100001

2?0?000100100?0???10?0???00011??0?????????0???0????0??????????????????????????20?110

0001112110211110021111122122002?0?????00??????0001?0???0?000?0100?11??1??01??000

00??????????0?1?0100??0?????00????????0???0??0??????????0??00????????????????00??0???

?????0?0??0????0????????????????????????????????????0?000???0????????????????????000??

?????0?0??000???????0???01?????????????201??000????01?????????0????00?0???0??00?0????

??00?????1??0?0?0?0???0???????????????0?????000?????????0?????0000?????00?????????001

?0??????0???1??????????000???

Baptornis

?????????????????????????????????????????????????????????????????????????????????

?????????????????2????????5?????????24?????????????????????????????????????????????????

??????????????????????????212??????????????????????????????????2???????????????????????

?????????[1

2]????????????????????11001??00??1??????????121011000012????012?0?????01?10?0???000?

???0?????????0???0??????????01?[0 1]1[0

1]???????????????20?11000011121102111100211111?21020?2?????????????????????????????

????????????????????????????????????????????????????????????????????????????????????????

????????????????????????????????????????????????????????????????????????????????????????

????????????0???????0?0??000???????0???01???????0?????201??000????01?????????0????000

0???0????????????00?????1??????0?0???0???????????????0???????0?????????0?????0000?????

00?????????0210???????0????????????????????

Ichthyornis

?????00????????1220?????????????????????000111????0???????????000??1?????1000?

?0210??10???1?01111?2100?1???5?000?21??34???01?100?001031110?011113003??0102?1??1

0212000????21023??3?12??10011??11?1213110???00?0??20?????00????0??00????21????????

???0?????????????012300[1 2]0?11???????????10[0

1]1?111011101001020???101?1200111?101[1

2]?211022010100001010?1111011211011110010110111010000101[0

1]10112111123120110011?12001[0 1]0010?112110211110021111[1

2]2?100002?0??011?100?20???0110??1??00?00100?11?01??01???????????????????????????0?

00?00??????????????????????????????????????????????????????1?0?????00??????????????????

?????????0????0100?????0?00?0000?00?????????000?0???????00??000??0?01?0000??????0??0

0111?00100?0?10201??000????01?0???????0????0000?0?0???0?0??1???00??0??1??????000??0

0100?0???001??01??2?0?00000?00000000????0000?????00?111?0?00001????10?1?0?0?11?0?

?0????0??0?

Iaceornis_marshii

?????????????????????????????????????????????????????????????????????????????????

????????????????????????????????????????????1101?010311??????113?03???????????????????

?????????????2??1??1???????21?????????????20???1??0???????????????????????????????????

???????????????????????????????????????????????????????????????????21?02[2

3]11???1101010?11110112101???????????????????????????????11131401110111020010??1?1

?12110212110021??????????????????????????????????????????0100?11??1??????????????????

????????????????????????????????????????????????????????????????????????????????????????

????????????????????????????????????????????????????????????????????????????????????????

?????????00111???????0?10???????????????0??0??0??????0???????????????1??????????1?????

??????00????0???00???01??????????????0??????1??????????????1???0?00????????????????11?

0??0?????????

Limenavis_patagonica

?????????????????????????????????????????????????????????????????????????????????

??????????????????????????????????????????????????????????011?13???????????????????????

?????????????????????????????????????????20????????????????????????????????????????????

????????????????????????????????????????????????????????????????????????????????????????

??????????????????????10000[0 1]010101011?11[1

2]313011?011???????????????????????????????????????????????????????????????????????????

????????????????????????????????????????????????????????????????????????????????????????

????????????????????????????????????????????????????????????????????????????????????????

????????????????????????????????????????0?0????????????????????????????????????????????

????????????????????????????????????????????????????????????0000???????????????????????

?????????????????????????????????

Lithornis

100????????????????01010100?00?111??2?02?00?0???????????????10000?00????02?10

1?1?????????1????????2????????[6 7]?1????????????[1

2]??11000010311110011?13003??000210101?20200102??2102?????12?01000???11?12130100

?????0??200?01?2010???0?0?0???21???12???????????00???00000??300?11212100?000111110

10110?11[0 1]1101?1111?01?10121012?0111[1

2]1211122110100?01011011110112101111100110001[1 2]1110000101010101111113130110[0

1]11102[0 1]0120110?11211020110002111122210010[0

1]?000011?1????????011?111??00??????????01???1???????????????????????????????????????

???????????????????????????????????????????????????????????????????0????????????????0??

???????????????????????????????????????????????0???????0?0?????????????????????????????

????1??000?????????????????????????????????0???????????????????????????????????????????

???????0???????????????0000????????????????????????????????????????????????

Hongshanornis_longicrest 101????????????????11010?????0?11???0????00???????????????????000?001??00????1

?1?10???0??0??01??1?0????????[2 3

4]??????????4???1??11?011103111000111130020?0112???01?20????????2??23?23??2??100?1

??????21[2

3]01000000000?000?01?1????????0?0????1?1?0????????????0????0000?1?300???012????????

???????????????????00??????1?????0?????110????????01?0??11???0?10???21000111???110??

?2????0????????????1??2000??000100?0??0?0??000????????1???1100??[0

1]?00??2?0???1????????????????????????0100?11?001?11??????0??????????0?????????0?????

?0???????????????0??????????0??0???????????????????????????????????0???????????????????

??????????????????????0?0?0???0?????????0????????????0???????0??????????????????01?1?0

?000?0?10???????????????????????0?????0?0???00????0??100?00??0?????0??????????1???00?

????????0?????????????????0??0?0?????????0??????????00100?????????0????????????1????

Liaoningornis_longidigitu

?????????????????????????????????????????????????????????????????????????????????

????????????????????????????????????????????????????3??1??0????????????????????????????

???????????????100?1????0?21211003000?????0??????????????????????????????????????0???

??????????????????????????????????????????????????????????????????011?01??????????0???0

?????????????????????????????0?????01???2??????????????????????????01?1?0??0011111102

01000?00???????????????????????????????????????????????????????????????????????????????

????????????????????????????????????????????????????????????????????????????????????????

??????????????????????????????????????????????????????????????????????????????????????0

????????????????????????????0?????0?0???00???????????00??0?????????????????????????????

??0???????????????????????????????????????????0010??????????0??????????????1??

Crypturellus_undulatus

10????012?020002?2001010100?00?111?12002100001011100010???1?10000?000??10

2?001?1?????????1110111122100?01107?110??12?24??121?11010010311011011113003??001

210001?202201120021023?23?12?01000100110121301?0???01000200001?20000200?0?00102

1???120??01??????0????00000103002112121[0

1]010001111101011001101111111110010101210101121121211112?101000210110101111121

00111110110001201100001110101011111031401011111020?120110?11211020211002111122

210010110??011?10102000001111110000??1100?10?01??11???????????????????????????????

?????????????????????????????????????????????????????????0?????????????????????????????

????????????????????????0??????????????????????????????????????????????????????????????

??????????2??????????????????????0?????????????????????????????????????????????????????

??????????????????????????????????????0?????????????0??????????????????????????????

Gallus_gallus

100???102?021002?2000010100?00?111?120021?0001?1000001????1?1001000000000

21101?1?????????101[0 1]1111221?0?01007?120??12?24??1[1

2]1?11012010311020011113003??001211111?2?2001120021023?23?12?0100011011??213010

010001000200100?20100200?0?0???21???121??01???0??00???00000103001102121211111221

101111111111110110001101011121010112011121111211010002101111111111210011111011

0001201100001011101011111131401001211021?121111?112110202110021111322200111?00

?0110001020?000111111?000??0010110?01??11??????????????????????????????????????????

??????????????????????????????????????????????0????????????????????????????????????????

?????????????0????????????????????????????????????????????????????????????????????????2

??????????????????????0????????????????????????????????????????????????????????????????

???????????????????????????0?????????????0??????????????????????????????

Crax_pauxi

1001011?2?021000??001010100?00?111?120021000011100000100??1?1000000000000

21101?1?????????1000111122100?111?7?120??12?24??121?11002010311121011113003??000

2100?1?2?2001120021023?03?12?0100011011?12130100100010002001?1?20100200?0?0???2

1???121??01??????00???000001030021021212111112211011111111111101100011010111210

101120111211112110100021011111111112100111110110001201100001011101011111131401

011211021?111111?1121102021100211113222001101000011000?020?0001111110000?11100

011?01??11?????????????????????????????????????????????????????????????????????????????

???????????0?????????????????????????????????????????????????????0?????????????????????

???????????????????????????????????????????????????2??????????????????????0????????????

???????????????????????????????????????????????????????????????????????????????0???????

??????0??????????????????????????????

Anas_platyrhynchus

100???102?021002?2101001100?00?111?10002??00?1?100010?0???1?10200000100?02

1101?1?????????101?1111221?0?011?7?11???10?2410121?11011110311111011113003??0012

000?1?2?2001120021023?13?12?0100011011?121311?0???010002101?1?20100?00?0?0?1021?

??121??????????00???00000113002102121111111221102111211111110110001111011121010

1020111211113120100101010?1[0

1]111112100011100110001201100001011101111111231401010111021?111110?11111010211

0021111[2

3]2210010210?0011?10?020?000111111?000??0100?11?01??11????????????????????????????

????????????????????????????????????????????????????????????0??????????????????????????

???????????????????????????0???????????????????????????????????????????????????????????

?????????????2??????????????????????0??????????????????????????????????????????????????

?????????????????????????????????????????0?????????????0??????????????????????????????

Chauna_torquata

000??1012?0210002?101010100?00?111?100021?00010100010100??1?100000000??00

21101?1?????????1011101112100?111?7?11???1012412111?10000010311111011113103??001

2101?1?2?200102?021023?23?12?0100011011??213010010000000200101?20100000?0?0?102

1???121??01??????00???00000123001102121[1

2]1111022110111121111111011000111101112101210201112111140101001010111111011121

10211100110001201100001011101011111231401011111021?111111?11211020212002111122

22001001000011000102000101111110000?11101?11?01??11???????????????????????????????

?????????????????????????????????????????????????????????0?????????????????????????????

????????????????????????0??????????????????????????????????????????????????????????????

??????????2??????????????????????0?????????????????????????????????????????????????????

????????????????????????????????????????????????????0??????????????????????????????

Pedopenna

?????????????????????????????????????????????????????????????????????????????????

????????????????????????????????????????????????????????????????????????????????????????

???????????????????????0?0?0??0010?????????????????????????????????????????????????????

????????????????????????????????????????????????????????????????????????????????????????

?????????????????????????????????????????????????????????????????????????????00?000????

?0??0????00????????0???11??????????????????????????????????????????????????????????????

????????????????????????????????????????????????????????????????????????????????????????

????????????????????????????????????????????????????????????????????????????????????????

???????????????????????00????0???00???????????00??0???????????????????????????????0???

????????????????????????????????????????0?10????????????????????????????

Epidendrosaurus

??????????????????????????????????????????0??1????????????????10??????????01????

?0???????0??????1??????????????????????????????????001??1??0??????001?????????????????

?????????????????????0????????0000030?0?0???0?????1???????????????1???????????????????

?????????3?0??????????????????????????????????????????0???????0???????????????????0??0?

0??????1???0?????????0???0???0????????????????0??????000??????????????????????????100?

00000??0?0??0?010????????00??????1???????????0?00????????????????????????????????????

??????????????????????????????????????????????????????????????0????0???????????????????

???????????????????????????????????????????????????????????????????????????????0011????

??0????????????????????????????0?????0?0???00?????????0?00??0?????????????????????????

??????0?????????????0????????0????????????????????0010??????????0?????????????????

Epidexipteryx

?01???????????????????1??????00?????0??2??0011?????????????????100?0?0????0000

1020???10000???????????????0?[0

1]????1?1?123??10??0????0101?010?0??????00?1???????????022?????011?03?0??????????0??

??011?0??0?10?000???00??1?1?0???00?0?00???0?0?????????1??2?????0???????2?0?????0????

??????0???????????????????????000?0????00??0?0??????????????0??0?0??????1???0?????????

????00?[0

1]0????????????????????????????????????????????????????10?0?0?0????10?00???0?????????1

??11??1?????????????00?100?????0??????????0?????????0?????0???????????????????????????

??????????????????????????????????0????0?????????????????????????????????????????00200?

?02?????????0???????0?1????????????????????????????0011??1???0?????00??????????01?????

????0?????0?0?????????0????0?0????0????????0???????????????????0???????00??????0???0??

??0?????????0??????????001?00?????????????????????0????

Incisivosaurus_gauthieri

?00?00101?001??1??100011101001001?010?12000011000101011010111121010000100

1?1?000210??00?0??????????????????????????????????????????????????????????????????????

????????????????????????????????????????????????00??00??01?0??0?0?????????10000000100

0102?00010?100??????0000000000100001??0000???0000101?1100??0000???????????????????

????????????????????????????????????????????????????????????????????????????????????????

????????????????????????????01020?00???0??000?0?0???????????11?1100000????1000000?0?

0000?000?0?000???1000??1??00?0????000?110??010??01?0000?00000???00?00????0100??01

11000????????????????001000000010000????00200??02?1?000000000?????00110???????????

????????????????????????????????????????????????????????????????????100?????????????0??

0?001????????????????????????0201100????????????????????????????????????????0?0?0?????

?0??????????000?0?

Citipati_osmolskae

?001001001001??221000101111?01011?000102100011000010001000110121120?0100

010111?1?????????1011101100101211001??201??002200111?0111200110100100?110000010

00211001??02012020??10111111?2101000000000?000000000000000000011020000000?0?001

000?000002011???0???[0

1]000020001220001000000001102?1??0000??0000010001010??0000020011000[0

1]0?00?0010000101020??????0????111000?0?0010000?000000000000?00100?0??0?0???00000

00????00000?2000000000000000000002?000?000000000020?000110??00[0

1]10?01101100000011?1100001????1000001?0?0001?11000?001???1002??0???0?0????000?00

0??000??01?0000?0000000000100?????100??0111000????????????????101000000010000????

10201???2?1?0000000???0????????????????????????????????????????10???00?00????????????

???????????????0?0?0???00100????000?00??0???0?00?????????0?0?0?00?0???0?00211???????

???0??00?000?0???????0??????0?0000100?0100????10?????????1?00?0?

Oviraptor_philoceratops

?00?0??????01?1????0??1??111?1011???0???1?0?11???01?0???0?11?121120?01?00?01

?1?1???????????????????????????????0????????????????12??1?????00???10??00?00??1??0????

????????????????????????0????0????0??0?????00?00000???2?00?0????????????10000?0?0????

???0??0???0????00???0?00????????10?0?00??0??00?00?1?1????00???00?100????0????????010

10?0?00?0?0????1?100??????1100???0????????00?????????000?0??00000???????????????????

???????????????????0??000?????20????1?0???02?0??1101100??0011?1?????1????10????1???0

?01????????01???????????????0?????0???00??0?0??0??00?????00???????????????????0???????

??????????????????????????????????1020????2?1?0000000?????????????????????????????????

???????1???1???0??????????????????????????????????????????100????000???????????0??????

??????????0??0???????211?????????00??????0??0??????????????????????00??0??????0???????

????00???

Microvenator_celer

??????????????????????????????????????????????????????????????21?20?0???????????

??????????011?0?11000121100???0?1?1002????????????00?000?11000?????0010002???0000?

????????????1110??0101100?0000010???????0??0???00??11?20?0??00???0?1??0?????????????

????????0???0?2200000????????????????????????????????????????0?0001???????????????????

?20000?000?01?????00?0??010?00?000000000000010000?????00????????0???0??0000020000

00000000????????????10??0??????????????1????????0??????????00?????????????????????????

????????????????????????????????????????????????????????????????????????????????????????

???????????????????????????????????????????????????????????????0???00??0001?01001?????

????????010000?0?????100???????01?01??0000????00?????????????0?????0????????1????000

?10?000???0010?0001????1??????00??00?10??00000000??000?0??00000??????????1?01??111

00??001??????

Caudipteryx_zoui

00010??????????????0?111??10?0001?10???21000???0?0????????????21120?0????????

0?1??0?????00????0??00????1???0???01?????30?????0???0??0???01?0???100000?000?1??????

0201202???1????101?11?????0????01000??1?00?0?000000?1102000??00?0?001000100000?00

????0???00?00000?1[0

1]2000000000???????????0??????0000????1000???0000?00?0000?0?0?????????????20??0?0?0

00?11??00???????0?0???00?00????????????1??0000???0000000?000000000??0?0?00????0000

000??0??2000000??0????????01101100??01?110???0?000?10???0000?????0????0?0?0001?00?

????0????????????????0????00???00??0?0??0??0?????????000?0????????1????0??????????????

???????????????????????????00201???2?????????0?????????0??0???????????????????????????

1???????00??000???0000???????????????0?00??0?0???001?0?0???00?0???0????10?????0???00

0??0?00?0???0?0???11??0????00????0?000?0000???0?0?????0?0?00100???0????????????????1

?10???

Ingenia_yanshani

?00?0????????????????1?1?????1????????????????????????????????21120?01000?0111

?1???????????????????????????1???01????2?00???1011120011??00000??100000100021??????

02012020011011?11101101?0000?00011?000000000??0000??11?2000?000?0?001000????????

???????????0002?0??2200?1000000??1?????0?0??0??0000?00??1?10???000??0??1??0[0

1]0??02??????0101??0??0???0?0?0101000?????1100???00?0?????????????????00????0?00?00?

000?0?000??0?0?000?0?0?0??00?0????00?000000????????0110??00?1?0?1101000?000?1?????

????????????????????????????????????????????????????????????????????????????????????????

????????????????????????????????????????????????????????????????????????????????????????

???????????????????0011000?001?0?01??101000??0101??11??0??????00000???00?????1?0000

00010??1?1?0??????10???0???0??0?000????11???0???010000000100000000??000100?000000

010??????????0??1?0??00?1???1??

Rinchenia_mongoliensis

?00?0????0??????????0111?1???1?11?00010??00???0000?????00??1?12112??01000??1

11?1????????????????1??????????????0?????220?????????2??1???00?0???1??0001000?1?????1

???????????????1?????????0????0??0?0???????0??00??001??2?00??????????????0??????00???

?????00??210??????2??0120????????10?0?10??0???0????????????0?????????????????????????

?????????????????????????????????????????????????????????????????00???0?00?????????????

??????????????????????????????????????002?0???????????00?1?1?00001????1??0001?0?0001?

11??0??01???1???????????0????000?000??000??01?00???0?000000?0????????100??0????0???

?????????????????????????????0?????????????????????????????????????????????????????????

?????????????????????1?1001??????????????????????????????0???????????????????0?????????

???????0???????????11????????????????????0??????????????????????0??????????0???????????

?0???

Conchoraptor_gracilis

?0010??????????1???00111?1???1?11?000??21000110?00????1?0????121120?010?0?01

11?1??????????01010110010??1?012?110??10????01?1?????20011000010???????0010002100

010102002020??101101[0

1]1?11010000??00010000010?000??000?001102?0?0000?0?0010001000002001???????0?0?02

00012??????000000000????????10???0100100????????000??0?010??????????????????????????

????????????????????????????????????????????????????????00?00??000002000000000000?00

00?002?01?????????0?020?000??0??002?00??????????0011?1?00001????1??00?1?0?0?01?11?

?0??01????0?2????????0????0?0??00??0?0??01?00???0?00?000?0?0??????100??0???00???????

????????????10???0???????0?????????????????????????????????????????????????????????????

???????????0?0??????????????????????????????0?0????010?????010?????0?????0???????10???

????00?0???????21?????????????????0?000000???????????????0?1?00????????00????????????

0?0?

Chirostenotes_pergracilis

?????1??01?01101??0???1?110?0??????????????????????01010??????21120?00000201?

??1????????????????1101?12????1?12???0????????????????101?1??????????00?100021?00101

022120201110?111[0

1]??01??100???00?100000200000??000?0?1??20?0?000?0?00100????????????????????000????

?[1

2]????10????????001????00????????????01?10???0????0011??00????????????????20000?0000

0?????????????????????????????????????????????????00000?00??00000?00?0????????000000?

0???0000?????001020?00???0????????1?????????001???????????????????1?0?00???0?????????

????????????????????????????????????????????????????????????????????????????????????????

?1000001000???????00200???2?1?000??0000??????????0???????0?0??00???1000?0??????????

????0??0??101000?????1?0100?0??0?00??00000?001?0??1?1?000?01?????1?0??????10?????00

0??0??????02?1???0110?1???????000000000???00????00??00010?0??0?????1?0??0???????0?1

??

Avimimus_portentosus

?00?0???10011?00??0??1?1???????1???1?????00?11??00100110??????2?1???00?0??01

11????????????011010110101?1?00??100????????????????????????0100??????????00?211?01?

?0201202010?011?0?1000100?0???00011110020???00??0?0?01?02?000000?0?001000????????

0?????????00?01??0?02001????????????????????0??0???00??0??????????02001000???????????

???????????????????0?????0?0?0010000?000000000????????????2???????????[0

2]0?000?0000020?010000000000000??00?01??0??????0???????????????????????????????1?0?

????????????????????????????????????????????????????????????????????????????????????????

??????100??0?1100????????????????????0000001?0?00???????????????????????????????????0

???????0001000001??????????????00001???????101001??0101?0100?0?000??001000??00????

???0??0?1001??101????0000100000?????1001101102?????101010000000????00?000???00100

0000000010?0????1??01????0??00?1???1??

Falcarius

??01?11100001111010?????1????00??????????00??????0000000??????100?000??0????

???001010001?1??0001110101210100012000100??0000??????000001000100010?0000100?01

1001010220301010100100010000000000000110000000000000??000?10?00?0?000?0?001000?

???????0?001???00?0?0?0??11?0??000???????000??00???0??00?00100?0?00??0000??0010000

[0

1]0??????????0100020000?00000001110?1?0?0010000?001000000000010000???0100???0000

000?0000000002000000000000?00000000?0000??00000?0?200000??0???00000?1100?000000

0???????????????????000?00?00100????0????????????????????????????????0?????0??????????

????01?00????00?00????????????????????????0000001010010????0??00000001???0??????????

????0?0???????0001000001000??1000??1010000000100000000000?0101?010000000001?0000

001000?0??00101000010?1?01?11?1000100100000000011101002?0?101111000000101000100

000??000001000?000000?0?10?10000?011011002?00?10?

Beipiaosaurus

0??1???????????????????????????????????????????????????????????10?10????????????

?1001001?100????1??????????????000??0220?????????0000??0????0?1000000?0000??001???2

0220??0??0????0??00?0??00??????0000????0???0??00111???0?0??0????0010?0??????????0???

???????0???0??????0?????????????????????????????????????00????001000?????????????00?00

????????????????????????????????10000???????????1??0100???000000???0??0??0?????0?????

??0000000000???0?0?00000????????????10?????1?1100??0?000?1?0?????0??????????????????

??0????????????????????????????????????????????????????????????????????????????????????

?????????????????????????????????0??0?00?????????????????????0???????0?????????????????

?????1???0?00??00???00?1????????10?????0?0?????????00???????1?1?0????????1??1?????10?

??000?0?00???????????????????0??0?01000?01010???0?11??10??0?1?0???1?????????????????

??1????

Segnosaurus_galbinensis

??????????????????????????????????????????????????????????????21??10?0000?000??

?0100?001????????1??????????1?0?0??????????????????0?10?1?00000????0???200111001020

20221??0110201101?11001?00?00?11000000021?0??00?01?1??0?0???00?0?010?00??????????

0?????????00???????0?????????????????????????????????0?0????0??????????????????????????

????0??????0????1????1???0010000?00100000?????????????????????????00?00000??10??????

00?????000000000??21?0????00???????????????????2?????????0???????????????????????????

????????????????????????????????????????????????????????????????????????????????????????

?????????????????????????????????????????????????????????????????????????????????10????

????????0?00???????21020010?0101??02???????????0?00??????????????0??01??0??1??1?????

???2???????0??110????????1???????10100??1?11122210111111112?111201??????????0???1??

???????????

Erlikosaurus_andrewsi

?0012???2?0?1?1??1010011100??0001?1000001000010000000000??111121001000000

20001?001001001?????????????????????????????????????????????????0?????????????????????

????????????????????????????????????0000?021?0??00??11??00?0?00????????????0?0000001

0010??00000?000????0?000002000001???10?0100???0000?0?10000??0000??????????????????

??????????????????????????1?0?0010000?0010000000?????????????????????????????????????

????????????00000000????????0000?0????000?????000?0?????????0???01?0001100????1?100

00?0?00?0?100000000???0??0?00??00?0????000001000000??00?0200?00000101000?00001?0

00??00?0?00????????????????00000001101????0???01001000200000000000000??????0??????

???????????????????????????0?00???????????????????????????????????????????0000???????0

0??0???0?011????????2??????????????11210?11???????1?0?1????????????????????????1?0100

1000????01??????????010?0?

Alxasaurus_elesitaiensis

??????????????????????????????????????????????????????????????210?100???????????

?1001001??????????0?01010000?1?0101002?0?1???????????0?000??0?10?0000120?11??0??1?

20?21???11?????????1????0?????????00000?10000??001?1??00?0??00?0?0???00??????????0??

????????0?????0????0??????????????????????????????????0??00????0??00??[0

1]0????????????????0????????0??11??01??????0?0???010000???????????10?0100???000000??

?0??0?????????????????????????????1000000?0????????0????????????????????000???????????

????????????????????????????????????????????????????????????????????????????????????????

?????????????????????????????????????????????????0??0020?????????????????????0?????????

???11101?????1??0?11??0?00??010?0?????01?????????2????????????????200??0????1?1??0??

0????1??1?100???????0000000??1??0???0?00???11?0????01100??11?1?????1111?????1?00???

10?1???0????????????1????

Neimongosaurus

??????????????????????????????????????????????????????????????21????0???????????

??0??001?1??000?110101?1?0?10?2?1?1?02200????????0001010000??????????????111001?2?

????????????????????0??00????0??000000021?00????0??1??00?0??00?0?????0???????????0??

?????????????1[1

2]200?0?????????????????????????????????????0?????00?0000?0??????????00000200?0?0000

0?0?11001?0?0?10?0???010000?0?00?????????????????????0???????????20?00???????0?00000

000?0210??????0????????0??????????2?1100?000?????????????????????????????????????????

????????????????????????????????????????????????????????????????????????????????????????

??????????????????????0????2??????????????????????0???????0??1??11???????0??01?10?0?00

???????21?2????????????2?1???1?00??00000?1???0??00????00????00??????100010?2???????0

0?????1???0?0111?111001011????1?1121?????111111????10????1??11??0?0??0?100???1?1??

Erliansaurus

?????????????????????????????????????????????????????????????????????????????????

??????????????????10??1??????????1???????????????0?0???10?0?0???00000????1?0?1???????

???????2??1????10?00?0?0001?0??00??????????00?????????????????????0??????????????????

???0??????200?????????????????????????????????????????????0????0???????????????????????

????????????01?0?0?10?0???010000???00010???????100???000000???????????20?00???????0?

???????????????0?????????????????????????????????000???????????????????????????????????

????????????????????????????????????????????????????????????????????????????????????????

?????????????????????????????????????????????????????0???????????????????????????????0?

0?1?0?01????????????????02?0???1?000??????????????00100???????0?????????00???100?????

00?11????????????????011?111111??11?1???0?1?11111011?0?????????1??????????0?1??????

Suzhousaurus

?????????????????????????????????????????????????????????????????????????????????

?????????????????0101??01000??0??1????0??????????001010000???????????200111001?2020

?2?10010020110101100?00??????????????????0???????1????????00???010??????????????????

??????0?????0?0?????????????????????????????????????????????20010000[0

1]????????????????20000?00000?01010?1?0?0010?0???0100000??00?????????????????????00?

000?00?1020000?????????????????????0??????????????????????????2???????????????????????

????????????????????????????????????????????????????????????????????????????????????????

????????????????????????????????????????????????????????????????????????????????????????

??10111100100000?11010?00??????021020010?0101?00201010????????????????????0???0??0

1??00?1????1000?0?2???????00????????????????11?100011????1112212111110?????????????

?????1?0??01102?0?2???????

Nothronychus

?????1112??11?1?0?0????????????????????????????????00000???????????????????????

?????1????????????10101?10?01?1?00?100220?001?????00000?100?00????00000200??10?1?2

020221100100201101?1100100?100011000000021?00???00??????0?0?000?0?010?00?????????

????????????0????1[1 2]200???????????1?????????????????????????????????0010000[0

1]0??????????00000200?0?00000?0211001?0?0?10?0???010000???00010???????000???00???0

0?000000?1020??000000000?00000000?0210??0?000?0????000????????0???1100?000000????

????????????????????????????????????????????????????????????????????????????????????????

????????????????????????????????????000?01100???????????????????????????????????????0?

??????0?0??1010110???0000?110?00001?0?0?021?20010?1101??02???1?10000?0?000?10????

?0?1?0000010?0??1?11?1?0010020000?00001110?1?2??????1?1110?0001???11112221011111

1?12101120??0????1?1?0???10?????1???10?

Enigmosaurus

?????????????????????????????????????????????????????????????????????????????????

????????????????????????????????????????????????????????????????????????111?01?2020221

100??0201101??????????????????????????????????1????????00???010???????????????????????

????????????????????????????????????????????????????????????????????????????????????????

???????????????????????????????????????????????????????00?000000?10???????????????????

??????????????????????????????????2????????????????????????????????????????????????????

????????????????????????????????????????????????????????????????????????????????????????

??????????????????????????????????????????????????????????????????1??1?????????????????

????2?020?10?1101?????????????????????????????????0??01?????1?????????????????????????

?????????????????????????1?122121100?????????????????????????????????????????

Nanshiungosaurus_brevispinus

?????????????????????????????????????????????????????????????????????????????????

????????1???????101?121???0????????????????????????????????????????????????????????????

??????????????????????????????????????????????????????????????????????????????????????0

?????1???????????????????????????????????????????????200??0????????????????????????????

??????????????????????????????????????????????????????0????????????????????????????????

????????????????????????????????2??????????????????????????????????????????????????????

????????????????????????????????????????????????????????????????????????????????????????

??????????????????????????????????????????????0??????????1???1??0??????????????????????

??21?2??10???01????????????????????????????????????01??0??1???????????????????????????

?????????1??????????????11222??11??????????????????????????????????????????

Therizinosaurus

?????????????????????????????????????????????????????????????????????????????????

?????????????????????????????????????????????????0010?0000????0?00200?????????????????

????????????????????0??0?11000000021?0????00??????????????????????????????????????????

?????????00??????????????????????????????????????????????????0??????????????????2000??0

0000?01?1001?0?0?10?0???010000?????010???????100???00000????????????????????????????

??????????0??0100???????????????????????????????00?????????????????????????????????????

????????????????????????????????????????????????????????????????????????????????????????

?????????????????????????????????????????????????????????????????????????????00?1010?00

100101??????????????????????????????????????????001?0????????0??????????10??0000?????

???????????????????1110111111????????????????2?111?0?????????1???01????0?????????

Haplocheirus

?0010???00010?0?12011011101112001?000020100?0?1010??0001?0010000010110110

?1000001101010100??10??0?0111201??0?00001?0???0?00???????100210001210?0?00010???0

01?00?101002010010?011000?00000000?0000?0000000??00?1110001100010001?0???0010?0

1000000000??0000001?00000?102000000000????00???00???0???000000??0??0??0000?000?0

00????0?????????????20000?000000010101??????100???000?00???????????0???00000??00000

00?00?000100200000???????000?0??0???0100?000???0?0??????????00?0??????????00000001

?00001??0?0000000?0?01000000?0??11???0??0?01??01?0????000001000000??00??100?00000

000000?000????00??0??0?0??????001????????0?0??0?0000????0???00000??00?00000??00????

??????000???????0??1???????????00?1010100??0100?00?0????????0?01??0???00?0?????0????

?00000?00?101000??00?0??010?11001001000100000110??00??0?101???00000???0000???????

??000????????00101???0?1?1?010?????0??0000??A

lvarezsaurus_calvoi

?????????????????????????????????????????????????????????????????????????????????

??????????????01000???0???0?20?0?2012????????????000?00????????0??1?0000101?00?2????

??????????????01?000??0??0??11000000??00????????1???0?0??00?0?????0??????????????????

????0????10????????????????????????????????????????????????00100???0???????????????2?0

00?????0?0?0????????????????????????????????????????????????0??000?00???2????00000000

?000???00?02???0?????????????0????????0??????????0????????????????????????????????????

????????????????????????????????????????????????????????????????????????????????????????

???????????????????????????????????????????????????????0???????0001?00????????00?10?0?

0???????????000?000?????????????????0??00?????00????????????0??0??0????????1000??????

?10?????101???????1????00?????????00000???????1???0?000?10??????1???0?0??0??0???????

??

Patagonykus_puertai

?????????????????????????????????????????????????????????????????????????????????

??????????????1?01?112????012010?2?2??????????????00210???210?????1?0???1????1010???

??20????21202??11000000000001210??0????001??0???????0?????????0????0????????????????

?????0?????01?????????????????????????????????????????????0?00100???????????????????20

000?000?00??????0?0?0000??0?0010000???0??????????1???????????0???0????1002000000000

00??00?0???????10??0??????????????????????????????????0???????????????????????????????

????????????????????????????????????????????????????????????????????????????????????????

???????????????????????????????????????????????????????????????????????00001?01100?0??

???000?1100??????11???0?0?01?01?1?000?10??1????0?00?????0????00?000?0??1???0??1001?

101??1?0?1111111????????????0?00000??0?????00??0000?00?0?00??1?????????10??1???0???

0????1??

Achillesaurus

?????????????????????????????????????????????????????????????????????????????????

???????????????????????????020???2??????????????????????????????????????10??01?2??????

?????????????1??????0??1?01000??0???00????????1???????????0?????0?????????????????????

?0?????????????????????????????????????????????????????????????????????????????????????

????????????????????????????????????????????????????????0???????????2????00000000?0??0

??0????????????????????????????????????????????????????????????????????????????????????

????????????????????????????????????????????????????????????????????????????????????????

????????????????????????????????????????????????????????????????????????????????????????

?????00?0??????????0????????00?0????0??????????????????????????????11????????????????1

1?????????????0????????????000???????1???0?00?????????????0?????0????????????

Mononykus_olecranus

??????00???????112???????????????????????????????????100????????????????????????

2????00???????1?1?11102001???2?10?2??????1???1000?000200030210?02201100??1????1?20

1000?2?????3??0??0210100111011212000030000001??00???1??0?0??00?0?0???00???????????

???????????????00011???????????????????????????????????????????0?0010?0?????210???00??

???2?010?0?0?0?010?011?0?0001000?001000000000??0000???30000???0???0??00???????200

0000000000??0000??0?00?0??0??000???????01?0?????0?0????????1102?????????????????????

????????????????????????????????????????????????????????????????????????????????????????

???????????????????????????????????????????????????????????????????0???????1001001000?

???????1010100000100?????011???0???01?010000010101?100000100????0??????00??1??0???

021?1110010111111111101011???????0100000000000?000?0000??000010000?000?10???????

?100?10100?0?01???1??

Shuvuuia_deserti

?00101002?010111120110001011?01111?10020100001010010010???1110?0010100100

2100??0211??000??0111111011102100?0?201012012110100?1000?00020003021??022011000

0101001?201000?2002203??03?02101001110112120000300000011000001100000010000?00??

001000000001?010??000000000?000100000000?101000?10000110?0111000??0[1

2]0????0100?0010000[0

1]0?0210?0?00?????20010?000?010101011?0?0001000?00100000?000?????????30000??000??

00?000?00?1020000000000000?0000000?00100000?0000020?0001?01?1010001???????11020

0?000?000??0?1??0000?0?00000?00000100???0??0?00??01?0????0?????0??000??00?0200?00

0000??0?0?0??00?000?001?0?0???????????????????000000000????????00000?000?0??000000

??00??????0?0???????00010010001011?0001010100000100??0?01110000???01??1??????????0

???0????00001????1??00??1??00110210?110010?1111111??0?01002?0??00??0?0000000?0000

00000??000?????00000010101?0??0?00110????00?1101?0?

Parvicursor

?????????????????????????????????????????????????????????????????????????????????

???????????????????10?????????1??2????????????????????????????????????????1????2?1000?

??02203??03??2??100011?11211000030??00?????????1??????????0?00??0???????????????????

???0?????0??????????????????????????????????????????????0?00?00????????????????????????

???????????????????????????????????????????????????????????0????????1?2000000000000??0

00?000?00?0???????????????0???????????????????1???????????????????????????????????????

????????????????????????????????????????????????????????????????????????????????????????

??????????????????????????????????????????????????????????????????1000?????00??????????

???????????????0???01??10??00?0?01??000??100???????????00??1????????1?111???????????1

1100010????????????0????????????0?????000??00000000010??????????0???1?0????1???1??

Albertonykus

?????????????????????????????????????????????????????????????????????????????????

??????????????????????????????????????????????????????????211?????1?0??????????????????

??????????????????????????????030?????????0????????????????????????????????????????????

????????????????????????????????????????????????????????????????????????????????????????

????????????????????????????????????????????????????????????????????????????????????????

???????????????????????????????????????0?02????????????????????????????????????????????

????????????????????????????????????????????????????????????????????????????????????????

????????????????????????????????????????????????????????????????????????????????1??????

????????????????????????0???00????0??????????????????????????????????11???11??1???????

????????????????????????????????????0????0???????????1???????????????????

Albinykus

?????????????????????????????????????????????????????????????????????????????????

?????????????????????????????????????????????????????????????????????????0?????????????

???????????????????1101??121100300000?????????????????0000?????0?????????????????????

????????????????????????????????????????????????????????????????????????????????????????

????????????????????????????????????????????????????????0??????0????????000000000?0000

000?00??0????00????????0???????????????????1??????????????????????????????????????????

????????????????????????????????????????????????????????????????????????????????????????

????????????????????????????????????????????????????????????????????????????????????????

????????????????????????????0?010000??00???????????00??1??????????????????????????????

?????????????????????????????????????????00000?10??????????0??????????1??????

Bonapartenykus

?????????????????????????????????????????????????????????????????????????????????

???????????????????112??0????????????????????????000210???????????????????????01??????

??????212?2??1????????????????????????0???????????0?0???????0?????????????????????????

???????0????????????????????????????????????????????????00100???????????????????20000?

000?000??10???????????????????????????????????????????????????????100?????????????????

?????????0?????????????????????????????????????????????????????????????????????????????

????????????????????????????????????????????????????????????????????????????????????????

???????????????????????????????????????????????????????????0?1000??1?????0?010?010????

??????????????0?0?01?????????????????????00?????00???????????0??????1???011???????????

??????????????????0??????????????????0?????0????????????????????10????0?????????

Ceratonykus

?0010???2??10?????????????????11???10?201?0?0?0100??0?????????????01?0????????

??????????????????1?1??????????????????????????10????0?2???0???????????????????????????

??????????????????1????????212?0003???00????????????????????0?????0???????????????????

0???????????????????????00???????1????111????????????????????????????21?????0??????000

0?0???01???????????????????0????????????????????????????????????????????????00000000??

000?000?00?0????????????????1????10?0??????????????0??0???????????????????????????????

??0?????????????1?0????0?????0??000??0??02???0?0000??0?0?0??00?000??0????0??????????

?????????0??0?00??????????0?0????????????????????????????0???????0001?????????????????

????????????????????????????????????0?0??1000???????0??????????????????1?????1?00?0???

??????????1???????????????????????????????????????00?00001???????????01???????????????

?

Linhenykus

?????????????????????????????????????????????????????????????????????????????????

???????????111?101?1??1?0???2010020?2???????10?0???1??????????0?21110??????????2????

??????????????0?1010001????2?2?0?030?0000???0????1??????????0?????0??????????????????

?????????01??????????????????????????????????????????????0?0012?0?????210????0?????20?

?0???0?0?0???????????????????10000???0??????????31??????00??0?????????1?200000000000

0??000?000?001?????0?0?????????1???????0????????????02????????????????????????????????

????????????????????????????????????????????????????????????????????????????????????????

????????????????????????????????????????????????????????0???????0001?01000?????0??????

1??????????????????????????0?0??00?0?0???000???00?0??????1??0????????????1011????????

1111?1???0??????????????0????????00???????????01??00?00001???????????0?1?110?0???????

??

Xixianykus

?????????????????????????????????????????????????????????????????????????????????

???????????????????102?00?21201??0???????????????????????????????????0?01?1??1?200000

?20022?3??03?02?010011101121211??3???000???????11???????00?0?00??0?????????????????

?????0????????????????????????????????????????????????????0?0010000???????????????????

?????????????????????????????????????????????????????????????00?000000?1?200000000000

0??000?000?0?????????????????????????????0????????????????????????????????????????????

????????????????????????????????????????????????????????????????????????????????????????

??????????????????????????????????????????????????????????????????????1000?0???????????

???????????000110000???01?010??00?0?00010?0??????????????????????0??1???1011????????

????111??11?????????????0??????????0?0000??000010000000001??????????0????100????1???

1??

Nqwebasaurus

?0????????????????02????2010????????0100?00??1?????0?00????????????????????????

02????1??????00?11001???1??????????????????????????0102?000??0?00?10110?????????????

?????????????00?????0?0000100010000000?000?1??00??????0?0?0?????0????01?00???00????

?????????????10200?????????????????????????????00????????????????????????0?????????????

20????0?0???0101?0??????10?0???????????????????????00000??00000??????????00??????????

??00?0????00??0?0??00000????????0??????0?0??????????0001?????????????0?0??????0???0??

????????0???0??????0??????????????????????????1??????????????????????000??0??0?0??????

????????????????00???????????????????????????????????????????0?????????????????????????

000??01?0?1?0?0????????????????????????0????00?0???00?????0?111?0???1??0??012?????10

0??00010000100001???????0?????????????0?????????????????0?000010??????????0?????????

??1?1???

Shenzhousaurus_orientalis

???0???????????????21000??10?0??1???000??00?000?0???????????0?00000000??0??0?

1?12?0??1????????????000??1???0???00000???0??????????????????????????010000000??1100

0100101?000001100??00001?0??????????????????0??1??1?0011?????00???0010??10000010??

??????00??0?00???0???00?000????????????????????00?????0??????0?00?0?100?0?0?0????????

?????????????????????????????????????????????????????????0?????0?00000?00000?0002?000

?????????????????????????00?????????????????00000??????????01?00??00000??0?0000?00?0?

0000000000??00???0??0??1???1??????????????????????????????????????????????????????????

???????10???????0??????????????????00?00??00?00?0????0?????????????????????????000????

???00?????????????????010100000?0001?00??0?????????????????01?????0?10??00??1??00?2?

0000????????100?0????????00?10????00????????00000000???00?????????????0???0??0???1??

?????????1???0

Ornithomimus_edmonticus

?00010?1101101?101021000?0101010110000000000000001000000??????00000000010

?1001?1???????0?001?1011000011100010100000000100100??????01120012000000?20200100

000011000110101100000110010000011000100010000020??00?011111110121110000?0?0010

001000000001?????0001?000000002100000000?????????0?0?10???000010110??????00000001

0000[0

1]0?0??????????????0??0?0?0?0?01?1001?0?00?000???0000000??00??????1??00000??000002

0?000?000002000000000000?0??0?????0??0??000?1???????0???0??0000000????????0010000?

00000?00?0000?01?0?0100000??0??00???0??0001??01?0????000001000000??01?00????00001

01?0000??000000??0????0????????1??????????1????????????????00210???0?00000?010??????

??????010?00?00001000011???????????????????10?00???1?????????????????????????1???????

0111100111111111?11000???????100??0???00?01????101?0???1??0?0000?10000000000????0

000000000000100?0????????1???????????10?01

Archaeornithomimus_asiati

?????????????????????????????????????????????????????????????????????????????????

?????????00?101100001110001?100000000?0??????????010200?20000?0110[1

2]0???000?01100011010110000010201000001100??000?0000020??0000?10??10??1?1??00??0

?001000??????????????????????????????????????????????????????????????????????????0?001

000?[0

1]0???????????????20?00?00000?0?0??01?0?0010000?000000000000010000???00000??000??

00???00000002000000000000?0000??00?0?10?000?????????????0??????0?0????????0001????

????????????????????????????????????????????????????????????????????????????????????????

????????????????????????????????????????????????????????????????????????????????????0??

?????0??1?00001?00??00000?00020010?010?010100000?01011?00000000000?000001000????

?001?101?001?11?0????000010000001100001??011???????110?000001100000000000??00000

0000?000?10??????101?0?1?000?000????1??

Anserimimus_planinychus

?????????????????????????????????????????????????????????????????????????????????

?????????????????????1?????????????????0??????????1?200?2????00?20200100000011000110

101????001?001????????????????00002???00????11??10??1?1??00?0?001000????????????????

?????????????????????????????????????????????????????????????????????????????????????0?

???????0??1?????????????????????????????????????01000??00000?????????????????????????1

0000??00??0??0??00?1?????????????????????????????001??????????????????????????????????

????????????????????????????????????????????????????????????????????????????????????????

????????????????????????????????????????????????????????????????????????????????????????

???10?0??1?1?????????????????????????1?0??????????11011011001??1?0????????100???01?0

0?????????????????????000?10?000?????????????????????010????????????????????????????

Struthiomimus_altus

?00010?110??0??1010210002011101?1100000000000000010??0001?01??00010000010

20001?1?????????001?1011000011100010100000000100100??????01120012000000120100100

0000110001101011000001?0010000011000100010000020??000011111110121110000?0?0010

001000000001??????00?1?0000??2210????0???????????0?0?1????000?????0?00???00?0000?0

000[0

1]0?0?????????????200?0?0?000?0101001?0?0010000??00000000000?100000??00000??00000

00?000000000200?00000000000000??00?021000000?10022000000?0??0?00?00????????00100

00000000?00?0000?01?0?01000000000000???0??0?01??01?0????0000010?0000??01?0000??0

000101?00?0??000000??00?0?0???????????????????1000011010?00????00210???0?000000000

????????????00??00?00001000011?????000000000200?110101?10100100?01011?0000000000

0001000100000111001111111?1?110000??000010000001100001000110100???1??0?00001100

000000000???000?000000000100?0?0?101?1110000?0000?1010?

Gallimimus_bullatus

?00010?110110101010210002011?0101100000000000000010000001?01000000000001

020001?1?????????00111011000011100010100000000100100??????0112001200000??2010010

0000011000110101100000110010000011000100010000020??000001111110121110000?0?001

0001000000001?????000110000000221000?00000001000000?0000??00000?0?10200??100000

0010000[0

1]0?0?????????????20010?0000000101001?0?0010000?0000000000000100001??00000??0000

020?000000000200000000000000000?000?021000000?1002??0000?00??0000000???????0001

0000000000??0?0000?01?0?01000000000000???0??0?01??01?0????000001000000??01?0000?

00000101000?000000000?000?0?000???????????????0010000111100000???00210?0?0000000

00000000????????000000?00001000011[1

2]000000000000020011?0101010100100?0101100000000000000100?10001110111111011001

?110001??000010000001100001000110100???1100000001100000000000??0000000000000010

000?001010111000010000?1010?

Garudimimus_brevipes

?000?????01101????02?00020101000??00000000000000000?00001?010000000000000

2?001?1???????0??0111????0001?10001?100??00?????10????????????????????????????000000

1100????????????0??001000001?000?000?000001000000?01??1110?2?1?0?00?0?0??00010000

0000??????0000100000010???00?0000?001000000?0000??0000010110000??0000000010000??

?0????????????????????????????????????????????????????????????????????????????00?00000

00002000000000000000000000?021?0????0000????00???0??001000????????0???00000000000

02?0000?00?0?01000000000000???0??0?01??01?0????000001000000??01?0000??0000000000

0000000000??00?0?000??00?010???????001000000010???0???00210???000000000000000????

????0?0?00?0000??0001110000000????????????????010100100?0101?0000000?000000100?10

001111??????001000?1?0001??0000???????????000010110100??????000??????????000000??0

000000000000010000?0010??01??0011??00?111?0

Pelecanimimus_polydon

?00???????1????????2100?2?1??0000??000000?0000??????????????0?000?0000???????

000211??0001??000???0?0?111?00???????????????0??0?00???120????00??0?2010???????????

?????????????????????????????????????????????1001010????0?????0?????0????10000??0?1?0

????00????0000?0?10???0?0??????????0?0?????????????????????00???0?10??????0??????????

????0??0?0?0???0???????????????????????????????????1??00000??00000????????????????????

??????????????????0??000???????????1?????0?00??????????001?00??00010??0?0000?00?0000

00000000??00???0??0?01??01?00?0?0000011??000??????????????????????????00??0?000????

??????0010000????0??????????????????0??00??00?????????????????00000000?10???????0?0?

?????????????????????0?01?????????????????????????????????????010?1??111??????0????0?2

?0?????000001100??????????0?1101??????????0?000????????????????????????1???0??????1??

????????111??1

Harpymimus_okladnikovi

?0?????????????????2100??????0??????000000000000??????????????000000?0000????

1?1200??1???0???1???0?00??10001?100000?00?001????????0?1?001200000000010?10??00??

?????1????????????00?00??????0??0?010000010??000001011????1?0???00?0?0???001??0000?

?1?0????00??0000??11?10000000????????????0??????????????0000??00?00000?0000[0

1]0????????????????????????????10??01?0?0010000?000000000000???0?010?00000??000000

0?0?0000?00?????00000000?00000?00??11??0000?0????????0???????000?????????0001?0000

00000????0????0??0?0?????0000?000???0??0?01??0??????????????????0???1?00??????0?????

???????00000??00?0??????????????????????????????????????00210??000????????0??????????

??0???????000??0000?1??0????0000??2001?00?01?1??0010?????????0??????????00?0??00011

00100011??0??0?11?00????000???000001000????011???0??01???00000110?000000000???0?0

0???0?000?100?01??1?1?01??00??00???1????

Beishanlong

?????????????????????????????????????????????????????????????????????????????????

?????????????????????????????????????????????????010200120000????0100?????????????10?

??1??00??????000001100?10001000?01??000?0???1???0??1?1????????01000????????????????

????????????210??????????????????????????????????????????????????0??0???????????????200

10?0000000101001?0?0010?00?000000000000010???????????????????0??????????200000000

0000?00000?00????0??0?0?0????????0?0?????????????????0?01????????????????????????????

????????????????????????????????????????????????????????????????????????????????????????

??????????????????????????????????????????????????????????????????????????????????????0

0000020011?????????????????????00?????0?00000?0???00?????00??1?0???0??1??????????100

0000?1??00?0?011?????????????000100??00????????????00000000001?????????1???10?0??0?

?0???1??

Sinornithomimus

?0001?????1?0????1?21000??1010001?000000000000000000?000??0???00000000?00?

???1?1?????????0001?0?10?0???100?1?1??0?00?0?0?100??????010200120000?011010010000

00110001101011000001?00?00000110001000?000?010??000001011100121?1??00?0?0010?01

000000001?????00001000000?02100000000?????????????00??000001?1?0??????00000001?00

0?0?0?????????????20010?00?00?0101001?0?0010000?0000000000??01000010?00000??00000

00?000000000200000000000000000??00??1100?0000?????????000???000000????????0001000

0000100?00?0000?01?000100000??0?000???0??0?01??01?0????000??10??000??01?0000??000

??????00000000000??00?0?0?0?????????????????10000????????????00210???0?000000000???

?????????0???00?00001?000?????0?0??000000200?100?01010100100?0?010000?000?00?0??1

?0?1?00111?1001110?1001?11?00???00001000000110000100?11??00???110000000110?00000

0000???00000000000001000??0?101?011?000?0?00?101??

Qiupalong

?????????????????????????????????????????????????????????????????????????????????

????????????????????????????????????????????????????????????????????????00??1100???????

?????01100?????????0??0001000002???00????????1????????00?0?0???0?????????????????????

???????????????????????????????????????????????????????????0???????????????????????????

????????????????????????????????????????????????????????20?0?0?00000?????00000000?000

0?000?02????????1????????0??????????0????????0????????????????????????????????????????

????????????????????????????????????????????????????????????????????????????????????????

????????????????????????????????????????????????????????????????????????????????????????

??????10100?00?0001??????????000?010001??0??????????1??110????0?????????????????????

1??01??????????????????????????0000??000??0000?00001??????????01??????1???????1??

Kinnareemimus

?????????????????????????????????????????????????????????????????????????????????

????????????????????????????????????????????????????????????????????????????????????????

??????????????????????0???00001???00????????????????????0?????0???????????????????????

??????????????????????????????????????????????????????????????0????????????????????????

??????????????????????????????????????????????????????0?????????0??????0000000000000?0

00?02?????????????????????????????????????0????????????????????????????????????????????

????????????????????????????????????????????????????????????????????????????????????????

????????????????????????????????????????????????????????????????????????????????????????

?????????0???????????????000??00000?00??????????????????????????????????????????00???1

????????????????????????????????0???0000???001???????????0??????????????1??

Huaxiagnathus_orientalis

?00?0??????????????0001???1???0?????00??0?????????????????????00?0010????????0

0010?1010?00??0???1????????0??????00???0?002010????000000000?00??010000000020?0??0

00100201?000012?0???0??????0??0?010000000?0?011??000????00?0??00?0?0010?0100000?0

?1?00????0????0000??200???0000??????????????????????????????????00?00?1??0?[0

1]0?0????????0000020?00?0?0?0?010100??????10?0???0???????????????????010????000??00?

00??00000??????000?0??00??0??0????100000000????????000????00000?0?00??00000?01??00

000??0?00?0?00?10000000000???1??????????????????????????00?00???????????????????????

????????????0?????????????????????????????????????????02?00???0?????????????????1?0?00

???????0?0??0000??????0??0001100???1?0?000??00000??0????????????0?????0?0???0?0?0?0

0?101000??01?0?00?0?00001000?000??0???????0?????10?????00????0?0?0?0000????0???0???

?0000100?????????????????????000???

Sinosauropteryx_prima

000?0??????????????0001???1???0?????0000??????00??0???????????00??01???????1?0

001001010?000?01??100????1?0?0????00?110000201??????00000000?10??010000?00020????

??0100201?000?11?0?1?00?0??000?0?01000000000?011??0000??000?0??00?0?000??0100000

00?1?00?00?????000?0??20000?000??????????????0???????0101????????0000?0??0?00[0

1]0?0??????????????0?00?0?00??010?000?????10?0???00?00????????????1??0100???0000000?

00000??0?2?????00??0??00??00?0???0100000000????????000?10???000?????????000?01???0

000??1??1????0?1?0?00??0??????0?????????????????????????00??0?????????????????????????

??????????0?????????????????????????????????????????02?00???0?????????????????000100??

?????0?0??0010??????0??00111?0???110?000??00000??0?0???0??0???0??0?00?0???0?000?00?

11100???0000?00?0?0?001000?0?0?0??????010????1100???0000???0?000?00000??000???????

0000100?????100?????????????000???

Compsognathus_longipes

?00?0???????????????00101?1??00???00000????000????000???????0?00000100?1?001

000010010101000?01??1010???1?0?0???000?0120002010?????0?000000?10????0?000000????

??0?01002010000012?0??????????00?0001000000000001100?00????0?0???00?0?00100011000

00001?00000000?0?000??020000?0000??0?0??0??????0???0000????01?0??0000000010000[0

1]0?0????????00?0?20000?0?0?0?010100????0?1000???0???00??????????????010????0?0??00

??0?000?00??????00??0?0000?00000?00100000000????????00????000000????????0000?001?0

01000?1?0100?0001000000000000??0???0??0?000?00?0????0000000?00?0??01?12????????00

0???????11?000?????????????????????????0??????????????????02000?00000020000?0???????

1?0000???????000??0000?0????0???????01???1????00????0??0?0?0???0??????0??0000?0???0?

000000?101000??00?0??0?000?0010???0?0????0????10????1100??0000????0???0?00??????00

???????0000100?0010100??1?0????????001???

Juravenator_starki

0000???????????????00010??11000?1???00000?0000?0?????00?????0?0000010???0???

?000100101??00??0???1???????0???????00??100002010????000000000000????0000000000?0

1100???????????????????0?????00??????0?0??000000?10?000?0??0??0??00?0??????01000000

0?1?00?0?000??0000???20000?000??????????????0????????????0??????000?00???00??0?0????

????[0

1]00002???0???????0101000?0?0010000??000?000?000???0?0????100???0000?0???00?000??2

??00??????000??0?0000?00100000000????????00???00?0?00???00??0?000?011000?00?01?010

0?00?1?00000000000?00???0??0?00??00?0????0000000??000??01?10????000??00???????11?0

00??00?0????????00?????????0??????????????????02000???0?????0???0???????0?010????????

??????????????????0000??00??1?0?000??????0????????0??????0?????0?0???00000?0??101?00

??00?0??0?0???0????0?0?0??0?0?????0????0?0?????000???0?000?0???????0??????????00100?

0?????00?1??????0???00????

Sinocalliopteryx

0000???????????????0001??0???000???00?000?000??0??????????0?0?000001???????1?

00010010101000?01??10?????1?0??????00??1000020?0?????0000000??00??0100000003?0?0?

?000110201?000012?0?????????00???0??000??0000?011??000?1??0000??00????010?01?0000

00???00?0000???00000??20000?0000?00?????0????00?????0????????????00000001000?[0

1]0?0????????00?0?20000?0?000?010100??????10?0????0??00???????????1??010????0000000

?0000000002??00?0????00?0??00?00??0100000000????????00??10?00000???00??00000?01??

?0?010?0?0000?000??0000000000???0???0??0?00?????0????0????00?00?0??0???2?????00????

???????11?0????0????????????0?????????0???????????????????2?00??00?00????????0?????1??

?0000??0??000?00010??????0??000010????1?0?000??00000??0?0???0??????0??0??0?0???0?0

00?00?100000??01?0?00?0?0?0010?0?0?0????0?0???0??0?0?00??0??0???00?000?0000????00?

???????000101?????10???1??????????000???

Mirischia

?????????????????????????????????????????????????????????????????????????????????

????????????????????????0??0?0???????????????????????????????????????0????????0001?020

00???012100100010110????0?????????????1?????????????????????0010?????????????????????

?????????????????????????????????????????????????????????0?1?0?????????????????????????

?????????????????????????????????????????????????????????0???00?0?000??????????????????

????????????????????????????????????????????????????????????????????????????????????????

????????????????????????????????????????????????????????????????????????????????????????

??????????????????????????????????????????????????????????????0???000??????????????????

????1??????0?0?00?000?1??0?0??????????????????????0??????0??0????0????0???????????1??

??????????????????????????000?0????00??00??????????????????0??????????0????1??

Ornitholestes_hermanni

?0002???0?0?00?1???0?010?01110001?100101?0??1000001?01011????0000001000?0?

000010?00101001?????011?0101210000??000?0010??1???????????????01?00?????????00001?

?1?0?0100101000001??0?1???????0????????00?00????00000?0010?00?0?0000?0?0000?01000

000001?01?00001000002?112000000010?0??000000?0?00???0000?0?0000???0000000010000[

0

1]0?????????????????????????????????0?0?0010000?000000000000??0??0????1000??0000000

?000?00000?0000?????????0000?000?001??00?0????????00??0???0001?0?????????0??00?1?00

00000000000000?000000000??00??0???0000?000?00?0????0000000?0000??01?0000?00?0000

100??00001?000?0001100?00000?01000?????000000000000???0???02000000000020000?0000

0?00000000???????0001000002?10?00?0??????10010?01??000000000???0100????000????0?0

0?0000?0000???????????001??1010?0000???0???0????0?00??001000000?00???000000???0000

00??000?????????0?1000?1001?0001????0??0?1010?01

Coelurus_fragilis

?????????????????????????????????????????????????????????????????????????????????

????????0??010010000121000?????0?0002????????????0?0??0?10001????0??????????????????

????????01100?000001?0?0000110?0?0????0?00??00???0??????????0?0????0????????????????

??????????11200?????????????????????????????????????????????001000??????????????????2?

????????????01000?0?0010000?000000000000010000???0????????0000?????????00200000000

0000??000???????????0000???????????0??????0???????????????????????????????????????????

????????????????????????????????????????????????????????????????????????????????????????

???????????????????????????????????????????????????????????0???????0000100002?????0?00

0?0??100100????????????0?0001?000000000?0000?????0????????????1??00??????????0000??

000???0??00?0100????????110000000000?00?????????00000??00000001?????????0?1?0?1122

1111??????

Tanycolagreus

?000????????????????00?0??????0???00110????????0?00???????????????????????10?0

0??????1?????????????0011?000?????0????0?0??0???????000000000011?1000000????????????

????????????110010000011000000010000000000000??00???0??000?????0?0???00???????????

???????1???1????[0

1]2000???0???????????????????00??0?0??????????0?00001000??0?0?????????????20000?0000

000101000?0?0010000?0000000000000100000??01000??00000??????????00200000000000000

0000000?00100?00000????????0?0?????????????????0000?????001000???????????????????0??

????021000000?000?0?????????????????0??0??00???????0?0?000??00???????????????????????

????????????????????????????????????????????????0??????????????????????00001????????00

11001001000000??????????00???000000000000000000?000?????00100000000??0??0?0?100?1

000000000000101000?????????????0000000000?????????0?0000000000010?????0??0?100101

221011???1??

Tugulusaurus

?????????????????????????????????????????????????????????????????????????????????

???????????????????????????????0??????????????????????????????????0?0??????????????????

???????????000?0?100??00110?0?1????0?????0????????????????????????????????????????????

????????????????????????????????????????????????????????????????????????????????????????

??????????????????????????????????????????????????????????????????2000000000000???????

????????????????????????????????????????????01?????????????????????????????????????????

????????????????????????????????????????????????????????????????????????????????????????

?????????????????????????????????????????????????????????????????????????????????????00

?????????????????000000000000?0???????0?????????0???????????????????????????000001???

1????????????????????????????????????0?????00??????????????????0001??1????1??

Zuolong

?0000??????????????0000010111?0???000?0000001100000?????1000????????????????

?00010?101??1?010001000????1???000000?0000??????????????????000?0??????0?????00?010

0?????????????01100110000110?00?0???00010?0?00?????0?0100??????00???0???0011000000

??0???00??1???0??0002000??0000?????????????00??0000000??????????010?0?10??0?0??????

??????????????????????????0??????10000?000000000000010000???????????????00?0?0?00?00

20000?????????0000?000?0?1???0??????????????0???000100????????0?00?????000000000000

0?00000001000???????1???0??00?0000??????????????????0?????0000?00000?0?000??10??001

0?0001101?00000????????????????????????????????????????????????0??????????100???????0

0000??0???000???0??????00000?????0?00000?0?0?0??0000000000????000?000?00??0????0??

???000??00?0???0???????????000011?????0?0??0000?0?00000??00???0?0???0000000????00??

0?????1?0?10??000??01?0?01??

Bicentenaria

???????????00?1??????0?0??????00??00?????????????00????????????????????????0?00

0000001????????????100?????????0?????????????????????0?00???????????0?1????0??10?????

???????????0???00000?10???0?110???????0??????????1????0?????????????????00??0???0????

???????????1???0??????????00????????0??????00???????????0???0010????0???????????????2?

??0???0???0?????0?0?001??????00000000?0??????????????????????0??0???0????2000?000000

00?????????????0?????????????????????0???????????????00????????0???????????0???00??0??

?????????????????????????????0?0000000???????????????00000000?1??????????????????????

???????????????????????????????????????1?2?00??000??0??????0???????????????????????1?0

0??0????00????????0??0?????????0000?02???00?0???????00????????0?????00??????0???00???

0???????00???00?????0??????00??00?0??????0??0?????00?????00???????????0?????0?0??0?1?

0?????

Kileskus

???????????????????0?01120111????????????????????????????????????????????????00

000?101????????????????????????????????????????????????????????????????????????????????

?????????????????????????????00?00??0??????????????????????????????1?0000?0???0??00???

???1????????0??001?????????????????????????????????????????????????????????????????????

????????????????????????????????????????????????????????????????????????????????????????

????????????????????????????????????????????????????1011201???000?0000?0000?0????????

????????????????????????????????????????????????????????????????????????????????????????

?????????????????????????????100010?????????????00?????????????????????????????????????

??????????????????????????????????00?????00???????????????1??????0?????????????????????

???????0??????????????????????????????????????????1?????????????????????0?????

Guanlong ?000201000000?010[0

1]100011201112000?001100000010000000000?10????00000010?00??200000001010?100?000

000000111000000000000?0???00???????000000100000?0100000003000010000100200000001

110010000011000?0011000010000000000000000000000000?0?0010001100000001100?00?11

101102?1120000?0010????0000?000?00??0000000??0?0????000000010000?0???????????????

20000?0?????0101?00?0?0010000?000000000000010000???01000??0000000?0000000002000

000000000000000000?00100?0000020?20?000?00??0001000???????00000000000112010?100

0?00?1000001010000?003??0000??0??00?10001000001100010??01?1000?00000000000000?1

000011?000000?00000?????????????020?01000100000000000????0?000000010???????010100

???????000010000210000?0?0000010001000000000000001000000?0110000000100000?00000

00?0001100000100?010010?1000100000000000010000000000?00100100000000000000000??

0000000000000010100???1?0011?1001??1000001??

Sinotyrannus

???????????????????0?01???101?????????????????????????????????00??0?1????????0?

0000101??????????????0??????????????????????????????????????????????0??00??0?0?000????

????????????????????????????????????????????????0??0?????00?????????1?0000?0??100??0??

???11????????00?001?????????????????????????????0??????????????????????????????????????

??????????????????????????????????????????????????????????00?000?0?0???????????????????

????????????????????????????????????0??????????00??????01?2?1??10???0??1?00????1??????

????????????????????????????????????????????????????????????????????????????????????????

???????????????????????0??????????????????????????0????????????????????????????????????

??????????0001?????????????????????????????0?0??????0?????????????0?1?????????????????

???????????0??????????????????????????????????????????????????????????????00????

Proceratosaurus_bradleyi

?00?2??????????????00011?01112000?00??????????00?00???????000?00000010?00?12

0000000101011?????????????????????????????????????????????????????????????????????????

?????????????????????????????????????????????00??0????0?0??0???????????110000?0?1100?

00?11?0?1?2??????00000100000?????????00???0??00??10?0????00?????????????????????????

????????????????????????????????????????????????????????????????????????????????????????

?????????????????????????????0???????0?????????????????00?0010112?10?1000?00?1?00001

01???0????????????????0?1000100000110001???????0???0???00000000??0?0????????????000

0??000?????0??0?0??????00100?????0001???10?00000001000?????11000???????????????????

????????????????????????????????????????????????????????????0?00????????????1????0?0???

?????????????????????0??0010????????????????????????????????????????1?0?1??????????????

???000???

Dilong_paradoxus

?0002?1000000???0?100011??1112000?00110?000010000000100???????00000000?00?

?200000001010?1?0000?0001?0??1000?????0??0?0?0??0???????0000001100?????0000000?00

??1??00110201?0?0001?00?000?0??00??00010000100?0000?00000?0?00000?000?0?00100010

00000001100?001111?110201?20?000000????00000???0?0???0000010110000???000??00??00

????0?????????????20000?0?0?0?0101000?0?0010000?0000000000000?0??0????1000??00000

00???0?00000200000000000000000??00?0?1000?0000?0?20?00???010000100????????000000

00010002?11?1000100?0?000000000000103??0100?00??00010111100001100010??00?1100??

000000000010????00000?000100???????????????????020?010?010?0????01001??10?10002?00

?11?????01110000000100000100001???????00000110001????10000?0001??000???011000000

000000??010000000??11000????1?01?01001?????00??00???00???00000?00100?00?0000010?0

?000000????00??0?000?0001?100???1??10112????0??0000???

Eotyrannus_lengi

????????????????????000120??12??0???110???????????0?????????0?000?0?0??1???200

00000101012?00?????01?0??1??????????????????????????000000010????????0?0????????????

?????????????????????????????0???00000?0?00???00?0????00?0?????0?????0?1?000?00??10?

?0121???111?0?211?0000?0???????????????0??00?00100???00??0???0?0010?0??0???????????

????200?0?0?000?0?0?0?0?0?0010000?0000000?0000???????????00???????????????0????????

??????????????????????0????????????????????????1????????????00?????1?00101???????00?0?

01????01000?1010??1??0?00?00????????????????0?????????????????????01?1??0????????????

??????001??????0?00????????????????????000?1002110100??????????2??00???00???00????0?

?????????00??011011?000???????????????????????????01??00?00100?000?0???0??????1?0??0

?00????1000??????0?00????????0000?00???0000100?00??0??????????0000???0?1????0??????

011??????1???0?1?1

Juratyrant

?????????????????????????????????????????????????????????????????????????????????

?????????0?00?0000001?10??00000??0???????????????????????????????????00?00?010000110

10110000011?01???0??10????0?????????????????????1????????00???0010???????????????????

????2????11??????????????????????????????????????????????0?00100?0?0??????????????????

????????????????????????????????????????????????????????????00?000000000?0?00?000??00

????????????1?????????????????????????1?0??????????????????????????????????????????????

????????????????????????????????????????????????????????????????????????????????????????

??????????????????????????????????????????????????????0????????00?100011?00100?0??????

??????????100000011001000?????1100001????????????????????0??01??1??1?????000????????

????0?????????????0000?0??????????000000??000??0000?????????????1??1??????11??0????1

??

Xiongguanlong

?0002???00000?????1?000220???0000?001101000?21000000?0001??000?????????????

??00000?101??2000000000000111000????????????????????????????????????????????0?12?01

000???????????????????0000110??????????????????0??????0?00?????000?????????1?000000??

?00?0?1121?2102011???0??0000?00?000??????00???0??000???????????00?00100????????????

??????????????????????????????????????????????????????????????????????00?0000000??2000

0???????????????????????????????????0???????000100????????????0010?10001????1?00?01?0

?01?0000100??0010?0100??0??0??0?????0???01000?0???0?0100???001?111110010????100?1

?11??00?100????????????001001100010?1???????????????????????????????2?000??00000001

1000011????????????????????????111??001???????01110121??????????????00??0???????????

1??1?010?10?????????????00??????000?1??00001???????????000000?????00???????????10???

?1?????????1???0?0?0???

Dryptosaurus

????????????????????????20??????????????????????????????????????0?0?0??2???????0

000101?1????????????????????????0???00???????????????????10???????10?0????????????[0

1]?0?0?2???????101?01000100010001000??2???0??????????0?0????????0?0???????????????00

????????????????11????0???????????????????????????????????0?????????????0??????????????

???????????????????0?0?0010000?00????????????????????????????????0????????00200000000

0000??000??0?????????????????????????????????????????0?01????????????????????????0????

????????????????????????????????????????????????????????????????????????????????????????

??????????????????????????????????????212?1?1??????????????????????????????????????????

????10?????????????????????1?0???012?1?0111?????1????0??????0?????????????0???????????

?????000000????????????????00?1???00?????????00??0000?00001????01??????????0?1??00?0

?0??

Appalachiosaurus

????????????????????0?0?201110??????120?????????????????????00000?000?0???????

?00001010?2???????????????????????0?0?00???????????????????????????????????????????000

010120?10???10??01000100010001000002?0?00??????0????0????????0?00100?11000000?1??

?1?021??????????????000??0??????000?????0???????????0000???0????????????0????????????

??????????????????????????????????????????????????????????????????00????????0020000000

00000?0000?000?00?????????????????????????????????????0?????1????????000?000?01?0101

11?101??1?0020?01?10??0?0???????????????????????????????????????????????????????????0

00000010100010?00?????????????????11?110??1?????????0??1?????2?0????????????????????

?????0??????????????????????????????1?01110???111111111021?0000????????????0?????0?0

???00???????????0000000???00??0?????0???????????????????00?0??00000001???0???????01?

????1??00000??B

istahieversor

?1102?????????10?2200002?01110000?001201000?21100000??????????00000000?20??

2?000000101012??0?????????????????????????????????????????????????????????????????????

???????????????????????0??????????????2???????10??00??00?0??0???????????1200000011?00

?00213?0?112??????0000000?????????10??0????00001???0?0????00????????????????????????

???????????????00??????????????????????????????????????????????????????????????????????

???????????????????????????11??????????0??????????????????0?1011001000?1010101?01011

101010101002010001001???10000??00000020000211000010000??1111111?1?1001011001211

111?01000?010011111??????11?111101?10111111100?11212101100112?10??2?00????111???

??????????????0111?1???????????????????????????011?????01101111?120010000???????????

?1??1?0?0???????????????????????????0??0????????????????????????????????????????11001?

?????1??????????000?11

Albertosaurus_sacrophagus

?1102??000000?10022000022011100000001201000021100000?0001000000000000002

0??200000001010120????0?10?001?100?0????000??0????0???????000000010000???01100103

1200110000001012001000110??0?00?1000??00100000200000001000000000000?000?0?0010?

01200000011000?0021310111210201100000000???00000100?00??0000010010000??00000000

10000?0?0????????0000020000?00000?0001001?0?0010000?000000000000010??0?????000??

?00??00?0000000002000000000000000000?00??0100000??020100?000??0??001100?0110?00

000101110110010000[0 1]1001[0 1]100101110102011100211[0 1]1[0

1]1101?001000000100010201002001100100011111011111111101011001111111000[0

1]000111[0 1][0

1]1111000010110111111110111111100111212111100?12?10?121011???????1???????????????

??1111102011111?1021111110211101??1??1???111111111122010000000??00000101?011010

?100010?000??00?000000100?000000??0?00??110??00000000??0000?0000000001011001111

010111????0100000011

Gorgosaurus_libratus

?11020?00000011002200002201110000000120100002110000010001000000000000002

001200000001010120?0000010000101000000?000000000000?00001000000001000000101100

103120011000000101200100011010010001000?0001000002000000010000000000000000?0?0

0100012000000110001002131011121020110000000000000000100000??0000010?10000??000

0000010000[0

1]0?0????????0000020000?00000?0001001?0?0010000?00000000000001000010??1000??0000

000?0000000002000000000000000000000?0010000000020100?000100??00110000110?00000

101110110010000[0 1]1001010010111010201120021101[0

2]1?01000100000010001020100200110010001111101111111110101100111111100010001111

1111??00110110111111110111111100111212111100112?10?121011???????1????1101????1?0

?0111110201011111021111010211101?011?1?2?111111111?220100000001000000101101101

0?100010?000010000000001000000000000?0000110?000000000??0000?00000000010110011

1101011110??0?00000011

Alioramus

?01020100000011002200?0?201110000000120100002110000010001000000000000002

00?20??000010101?000000010000111000000?00?00?0??000??????????????????????????????1

20011000[0

1]001012?010??????001000100??0?01000?02??000??10??000000?0?0000????010??110000001

10001?0213101?12102????000?00000000000100000??00000100?0000??00000?00100?0?0????

??????????????????????????????????????????????????????????????????????????00?0???000??2

000000000000000000?0??0?1?0????0020110?00???0??0011000???????0???00111?2????00111

0111110?0111010100120121001021110101110001000010200001000000101101112011111111

001011001111111?101110011011101000110110111101101110111100111212111110??10000?

??000001000011110?101221011111?????????????????1111010??????1?1111121??00?111?12?

0100000??????0???0?1?1?010??000???????????0????01?0000?000000?0????????????0000??0?

?00???0?000?1??1101111?1?1??1011??0?000?11

Teratophoneus

?1?0???0???????0??2??????0111?00????1201???????0000???????????00??000?????????

?000010101??1????????????????????????????????????????????????????????????0????????????

????????????????????0??????????????????????????0????0????????????????1?0000?01????????

?3?????????0???000????????????10??0???00?001???0?????0????????????????????????????????

???????????????1???????????????????????????????????????????????????????????????????????

?????????????????????100???????????????????????????????????????0????0?01??????????????2

?021?0001??10??????????0???01?1??????0???01??1?1??21?111?100111101211??????????????

???????????011?1?120?1????1??1100?11?????????11????????10???????????1????????????????

?1?01???1????????????????????011?1?1???????????????000??????????????????0?0???????????

????????????????0??0????????????????????????????????????????????1??????1??????????00??1

?

Daspletosaurus

?110201000000?1002200002201010000000120100002110000010001000000000000002

001200000001010120100000100001?100?0????000??0????0???????000000010000???1110010

31200110000001012001000110??0100?1000??001000002000000010000000000000000?0?001

0?0020000001100010121310111210201100000000???00000100?00???000010010000??000000

0010000?0?0????????0000020000?00000?0001001?0?0010000?000000000000010??0????1000

??000??00?0000000002000000000000000000?00??01000000?020110?000??0??00110000110?

00000101110120011020110021110211110102101201210101[1

2]111111111111101101020000211001011[0

1]11211212111111100?111012211111110111001101111[0

1]100111110111101111111111100111222111101112?10012101100011101111021111???????1

11111?211?111?112111??1??111?1??1????2?11????11?12???00000??11000????1??1?010?100

0???000?1000?0??????0?000000000???0011000?0????????????????????????111011110??1????

??????000?11

Tyrannosaurus_rex

?12020101100011002200002201010000000120100002110000010001000000000000002

001200000001010120100000100001010000001000000000000?0????0000000010000???111001

0312001100000010120010001101001000100010001000002000000010000000000000000?0?00

100002000000210001012131012121020110000000000000000100000??0000010010000??0000

000010000[0

1]0?0????????0000020000?0000000001001?0?0010000?000000000000010000????1000??000?

?00?0000000002000000000000000000000?0010000000020100?000?00??00110000110?000001

1111112001102101[0 1]0[1 3]01102111101021112010??10121011111011[0

1]1101111021010211001011111211[1

2]1211111110011112123011111111111011011111111111110110201111111111111211222121

101112111112112111111011111021111211111111111102110111111211111102111111112211

2111111111112211000000010000001011011010?1000100000??0000000001100000000000100

001100?10000000??00000000000000101110111101011110110100000011

Tarbosaurus_baatar

??2020?011000?1002200002201010000000120100002110000010001000000000000002

0012000000010101201000001000010100000?1000000000000???????000000010000?01111001

0312001100000010120010001101001000100010001000002000000010000000000000000?0?00

100002000000210001012131012121020110000000000000000100000??0000010010000??0000

000010000?0?0?????????????20000?00000?0001001?0?0010000?000000000000010000????10

00??0000000?0000000002000000?00000000000000?0010000000020100?000?00??00110000??

????0001111111200110210111301102111101021012010??10121111111011111011110210102

110010111112112121111111000111212301111111111101101111111111111011020111111111

111121122212?101112111112112111111?111?1021112??1??1?11111102110111?11211111102

1111111122112111111111112211000000010000001011011010?1000?00000010000000001100

00000000010000110??10000000??0000000000000010111011110101111011??00000011

UzbekAll

?????01001??????021?????20111??????????10000???????0??????????????????????1200

?0000101?12????0?0?01001110???????0??000?????????????????????????????0?0????????????

??????????????????????????????????????????????0???????0?0?00???????????1?0?????1??0???

0???1?????????????????????????????00?????0??00????????????0??00100????????????????????

????????????????????????????????????????????????????????????????????????????????????????

?????????????????????00???00???0???0010???????????010??????????00??00??0100?01??00???

??????????????????????????????????????????????????????????????0?01100?1????????????????

????????02??01000?0???100??????????21?11?1??11??????21000????????01??00?11?????0?0??

??????????????????????????????????????????????????00????????0?????0??????10?1000??????

???00????????0???0??000?00???????????????????????????????????0????1???????????????0???

0?

Moros_holotype

?????????????????????????????????????????????????????????????????????????????????

????????????????????????????????????????????????????????????????????????????????????????

???????????000011?0???00???0??2???0??????????0??????????0?????0???????????????????????

????????????????????????????????????????????????????????????????????????????????????????

?????????????????????????????????????????????????????????????????20?0????????0???00??0?

????????????????????????????????????????????????????????????????????????????????????????

????????????????????????????????????????????????????????????????????????????????????????

????????????????????????????????????????????????????????????????????????????????????????

?????????????0??10???0???????1001?????????????????????????????????????????????????0???

?????????????????????????????????000??0?001?????????????????????00??????

;

ccode + 2 15.18 23 24 26 37.39 44 62 65 71 73 88 102 107 110 113 116 118 120 122 129 145

147 151 153 160 162 165 167 168 170 174 177 178 180 194 196 199 216 221 231 233 234 237

241 249 251 254 255 260 261 264 267 269 278 286 291 298 308 315 318 320 325 327 346 350

358 363 378 383 384 386 392 396 400 408 412 414 416 419 424.427 432 433 442 444 445 458

461 473 480 483 486 492 497 518 538 543 554 555 558 576 577 603 611 620 626 629 633 640

646 649 663 667 668 682 692 693 696 700 709 710 713 725 726 778 803.805 816 821 840 841

*;

proc /; comments 0

;

**Modified matrix Carr and colleagues37 with *M*. *intrepidus*.**

xread 386 33

Allosaurus

101000210000000000101000000000000100000010000110000000000000002101110000

100100100010010001???0?0000000000010?0??0100?0000000?000000000000000000?1100000

1000000000000?0?01000000000000????000?0001000?00100?00000001000?000000000000000

00000000000200000000000000000000000010000010110000002001000[0

1]0000100000000010000000000001001010?000000010001??001000?00010100010000000102

00110000000000001010

Maniraptora [0 1]000000000?000000[0 1]00000000?000000100?00?000011100[0

1]0000000001000000??1??00[0

1]00000?01000?01?00??0?0000000000000?0??0010?0010000?00001000001010010[0

1]000????00000000000000?0?00??0[0 1]000??001000?0?0?0000002[0 1]1[0 1]000?1000[0

1]010000?00000000[0 1][0 1]0000000000000000001[0 1]0000000?0000000[0 1]0001000000[0

1]00[0 1]100001?111100[0

1]1000?000000000000100000000000000000?000000000000??100001100??0110000000001000

01001000100000000000

Ornithomimosauria [0

1]00000000001000000?00000?1?000000000?00?0000100000??00000001?00000??1??0000000

0?0210??00?00000?0000001000000?0???010?0000?00??0000000?01000000[0

1]000??00000?0000?00000?0??0?00?0??00001000?????0?00002000010?00000000[0

1]00??????0011000000???0001000000000000000?0??00000?10?000?00?00000000000000100[

1 2]00000000000000001000000[0 1]000[0 1]010100?010001010[0 1]00??000000?0[0

1]00011001100000000000001000100100?00?00

Compsognathus

0?000100000000000000000010?000000100?00?0?000000?1?00000000??02000??1???0

00000??0010??00?0?????000000000000?10???010?1000??0??000??0?000?0?0??10????0000??

0000?0?000?0????0???????00?????????0???????????????????????????????????0?000000000[0

1]?000000???0?????0??0000[0 1]0[0

1]0?0??????0?0?0000??00000?000?0?0??0000010000?0??00100?[0 1]000000?0[0

1]0??0010000??0?0000?00?0010000?0?00?00????0?1?00?000?0????00

Proceratosaurus

000?12000001101112?01000?0?001000100?00?1?00000010???01????0?0?????????????

????????????0?10000?000001010000?1??????0???0???0?0?????100000000000???0??0????????

?????????????0?000??000?????0??0?0?????????????00?100??????????????0001???110?1?0000

00001000???0??111000101??????????????????????????????????????????????????????????????

???????????????????????????????????????????????????????????

Kileskus

0???12?????110111200100??0?00?000?00?0000?0?000??000????????????????????????

????????????????????????????????????????????????????????????????????????????????????????

??????????????????????????????????????????????????????????????????????????0100010??????

?????????0??0??????????????????????????????????????????????????????????????????????????

??????????????????????????????????????000??????0

Sinotyrannus

0???12??????10?1?1?1110??0?0??00?????0??1?0?0?????????1???????????????????????

????????????????????????????????????????????????????????????????????????????????????????

???????????????????????????????????????????????????????????????????1???????????????????

????????0?01???????????????????????????????????????????????????????00?100?0?1?0100010

00??????????????????????????????????????????????

Yutyrannus

10101221?101101111?11002?0?000000100??1?1?0?000010????110??000?011111000??

0000??0210???0?1000???0?011121?01?12100002?0000??1????00?20?00?0?0????????1???????

11?2???10?101?????????0010000????????????????????????????????????????01010?0?11?1?01

020200100???0?0?11?00000?000?00??01?0100???00100?1????1000000000000?0011?000?000

00010??10100110000000??011?0?101001001?01??1???0[0 1]01010??00?00?0?00

Guanlong

000012?00000101112001000?0?00100?100?00?1000000?10?0?010000000?0012?1?001

10000???010??00?100011000001010000?10???010?1100000?0000001000000000?0?00??0000

000011?0000100000?0??00000?????????????02001?0?010??0000100000?000??0????0000????

10?1?0000000010?????0??101010?010????????00000001000001010000?0?0000001000000100

000000000010000010010000000001010000000000001?011000000001010000000?00?00

Dilong

000002000001101002?01001?0?001000000100?1?00000000??100000000001012?1?001

00100??0010??000100011100001010000?10???000?1110000??000001000000000?100?????000

00000?0000101000?0????????????????????02001?0?0100?0?0010?0?????????????01001??100

?1?010002?00?11???0??10101000100000010000000001000000????????000000110000001???0

??0100001000?0??0?0001110??10??00010??000???011?0000000?00010000??01?00

Eotyrannus

0???0??????11?100101110??????????????00?0?0?1???????2?01010000?101001?001??1

??0???000?00????????????????????0???????????????????????????????0011?1???0????????????

????????????????001??????0?00?????????????????????????????????????000?10000?02110100?

?????????2?2??0?010???00?0?0???00???1?0???????????00???011011011??0000???00?11?????

???????11???????????????????????????????101???000?001?00

Juratyrant

0????????????????????????????????????????????????????????????????????????????????

????????????????????????????????????????????????????????????????????????????????????????

????????????????????????????????????????????????????????????????????????????????????????

????????0????????000?00?10000100?00100?0?????????????????????????111000010010001111

11??10001100100101??????110000101?????????????

Stokesosaurus

0????????????????????????????????????????????????????????????????????????????????

????????????????????????????????????????????????????????????????????????????????????????

????????????????????????????????????????????????????????????????????????????????????????

??????????????????????????????????????????????????????????????????110?001001000100111

1????????????????????????????01?????????????

Aviatyrannis

0????????????????????????????????????????????????????????????????????????????????

????????????????????????????????????????????????????????????????????????????????????????

????????????????????????????????????????????????????????????????????????????????????????

??????????????????????????????????????????????????????????????????100?000001000001110

1?????????0???11????????????????????????????

Timurlengia

00?????????????????00000??????00??00?0100?001?1?0101??????????????????????????

????????????????????????????????????????????????????????????????0?????1?010?210011?1??

???????????????????????????02?0200?11001000??0????01000??0111??????????1??121?11?1??

11??????2?210??1?0????????????????00001?????????????????????????????????10????????????

???????????????????????????????????????????????????0

Moros_holotype

?????????????????????????????????????????????????????????????????????????????????

????????????????????????????????????????????????????????????????????????????????????????

????????????????????????????????????????????????????????????????????????????????????????

????????????????????????????????????????????????????????????????????????????????????????

?????????????1??100?[1 2]??0?????1?1??1?01?0?

Xiongguanlong

0001000000?1102001????0??0???1?0??00?01?0?001?0?01??1?0001010??0000????01?0

100???000??0??0??????0???0111000??0??0?00?0110000???000?21?111110100?010?1??????10

0?10?1111?0??10?0?100????????????001002?00110??000?10?1?????????????????????????????

???????????????2?2?01?0?0??00001001000110000010??????????????????????????????????110

?101????001111?0?????????????????1111001210??????????????????

Dryptosaurus

1?????????????????????????????????????????00????????????????????????????????????

????????????????????????????????????????????????????????????????????????????????????????

?????????????????????????????????????????????????????????????????0?0??1212?1?1?????????

??????1?00???????????????????????????????????????10100?????1?????11???????????????????

??1???????12?????????1012??1?0011111?????1???

Appalachiosaurus

1??1?0?????????????000001001??000100?01?01001111???1200101?1?1?001101?002?0

1010???1?0?0???????????????????????????????????????????????????????????????????????????

???????????000000010100010?00??????????????????????????????????11?110??01?1?????????

?0??1?????2?2?0?0????????????????????????????????0?????????????????????????????????????

??????????1???????12111?1111110????11?1111111110110?0

Bistahieversor

1?0?0011010111200110000010?101000210101?0100111102??20010101101001100110

2000010?02110??1000000?00?0001210001021100010001000001??10?311111110??1??102110

10021001221111110101??00000?010001110??????1???11?111111[0

1]101?1110110011?1?1111100?011001212101100112?1?0?2?2?0100?????111???????????????

????????0111111????????????????????????????????????????????????????????111?1????101?10

111111?110001

Albertosaurus_sarcophagus 10110011010111200110000010011[0 1]1002001[0

1]100100111102112001010111100111011021[0

1]1111002110001000001010101012101010200011100?100010??110?31011111011111112110

1012100112111111010101001000111[0 1][0

1]1110?00110121111011111101111111011001????11111001011101212111100?12?1?0?21210

?0101??????1???11????????1?????????01111110212101111111?0101121011110110111010000

?1211111012111?021???1???1110111111111112001

Albertosaurus_libratus 101100110101112001?0000010010[0

1]1002001010010011110211200101011120011101102001110?02110001000[0

1]010101010021010102000111000100010?011003101111101?1?111?11010021001111111110

10100001000111111110?001101211110111111011111110110011111111110010111012121111

00112?100?2121010101??????1?0111????11101?????1?0??11111102121010111111010112101

111011001101000011211111012111?0111?11?2??110111111111112001

Lythronax

1????0???1?????????00000?0010?00??10?01101101?1102112?011001?1?0????????????

?????????1???001010?02010121000?0??????????????????????????1?11?11?1?1011?0100211?1

?11?111????????????????????????????????????????????????????0?1??????1?????01?0?1222?2

11??????1???????1?0?????????????????????????????????1??????????????????????????????????

?0??????????121111?11?????????????????10?111?1??????0??

Alioramus_remotus_+_altai 00010011?11????????0000001111110010[0

1]1111010011110211210111010120111100002101121011110101110001100101012200010100

00010011001112011113201[0

1]11101111110111010021001121111110101??101110011011101000110121111011111111011

011111001111101111001011101212111110??100000????0?10000010001000111110?11011?21

011111??????????????????????????10?111011?0110100????????????21?111?111111211???00?

11111?11??01

Qianzhousaurus

000?00?10111122001100002?1?111101101111101001111021121011101012011110010

2100111?1111???1?1000?1002?10122000?020000010001001112011113201111101?1?110?110

1002100?1211111101010????????1????????00110???11101??11111?110??11?0?1????0111000

10111?121?11??1011100?00????0?11?01011??1?0?111?102?1011??????111111?110?????????

??????????0???11?1100?10??000?????????????????1??1012?1110?0?11111????0??

Teratophoneus

1?????1??1??????????000010??1??????0?11?01001??102?1???1??????2?011100102?01

010??201010?0???????0???0121?1???201000200?1011???0?1013112111101011?10111011021

11110111111?1?1????????????????????1?0121111?11110102011?11?1?0011?1?1??0100?011?

01222?1???111??1???????11000?????????11?11?102???????????111????1?0211111011???????

???10??1?01101?1??0000?1211111112?11101112111211?10?111???????????

Nanuqsaurus

0????????????????????????????????????????2???????????????????????????????????????

???????????????????????????????????????????????????????????????????1?011??11?1?2101110

0??1?????????????????????????????????????????????????????????110?10?10????????????????

????????????????????????????????????????????????????????????????????????????????????????

?????????????????????????????????????????????

Daspletosaurus_torosus

111100110101122001110002000111101200211102201111021121010211112011110011

2110121112111111111[0 1]111012010121000102111001100101[0

1]11211111321211110111111011?0101211112211111101010110111001100111110011?12111

10111111[0

1]1011111111111????11101111011011222111101112?110021210101010001111011111110211

110??????1?1111111?212111?11111?111112101111011011101?0001121111111211111?12210

?2?111??1111111?11?1??

Daspletosaurus_horneri 111?00?10101?2?00??10002000111[0

1]0120021110220111102112101021111?01111101120100211121111111111111112??0121000

?0211100110010111121111132121111110111101110?012110122111111010101101110011001

1111001101211110111101?101??1?111111????1?1011110110112221211?11121110?2?210101

01111?????01?11?10211110???????11???????11?1?1?????????????10??11????1?1???0????????

?111????1?2122111211110111111111111111

Iren_Dabasu_taxon

0???????????????????????????????????????????????????????????????000?1?0?2?010?1

0????0????001010????????1??010??????????????????????31???1110????????????????????????

??????????00???????????????????????????????????????????????????????????????????????????

?????2?2????10?????????????????????????????????????????????????????????????????????????

????????????????????????????????????????????????

Zhuchengtyrannus

1??????????????????1010??0?????1??00??110?111?1?0211??????????????????????????

????????????????????????????????????????????????????????????????????????????????????????

??????????????????????????????????????????????????????????????1111?01101??????????????

????????11010??????????????????????????????????????????????????????????????????????????

???????????????????????????????????????????????

Tyrannosaurus_bataar

111100211111222001110112001110111211301102111111021121010211012010??1?10

201002111221111101101110121101211010021100021001111112111013212111101?11110110

1100312122201111111111?1111110110111111111111211110111110[0

1]2011111111011111?1111111201101122212?10111211111212111020111111?1111111?1021

11111??1??1?11111110212111011111?111112101111011011101000011211111112111112122

111211110111111111112111

Tyrannosaurus_rex 1111002111112220011101121011101112[0 1]0[1

3]01102111111021121010211112010??1?102010021102211111011[0

1]1110121101211011021100021001111112111013[1

2]1211111111111011111003121222011111111110111111011011111111111121111011111002

011111111011111111111112011011222121101112111112121110201111111111111111021111

012111111111111102121110111111111112101111011011101000011211111112111112122111

211110111111111112111

;

cnames

{0 general_1:_body_size;

{1 skull_1:_occiput_orientation;

{2 skull_2:_general_shape;

{3 skull_3:_rostrocaudal_length;

{4 skull_4:_bony_naris,_length;

{5 skull_5:_bony_naris,_cdl_mrgn,_posn;

{6 skull_6:_orbital_fenestra,_shape;

{7 skull_7:_orbital_fenestra,_size;

{8 skull_8:_orbital_fenestra,_orientation;

{9 skull_9:_laterotemporal_fen_long_axis;

{10 pmx_1:_nasal_processes,_orientation;

{11 pmx_2:_nasal_process,_fossa_or_foramen;

{12 pmx_3:_body,_dorsoventral_height;

{13 pmx_4:_maxillary_process,_orientation;

{14 pmx_5:_tooth_row,_orientation;

{15 pmx_6:_narial_fossa,_form;

{16 pmx_7:_narial_fossa,_extent;

{17 pmx_8:_rostral_margin,_shape;

{18 pmx_9:_palatal_process,_position;

{19 mx_1:_promax_fenestra,_exposure;

{20 mx_2:_promax_fenestra,_position;

{21 mx_3:_max_fenestra,_location_A;

{22 mx_4:_max_fenestra,_location_B;

{23 mx_5:_max_fenestra,_length;

{24 mx_6:_NEW_max_fenestra,_size_in_juveniles;

{25 mx_7:_max_fen,_length:height_ratio;

{26 mx_8:_max_fenestra,_posn_in_mx_antrum;

{27 mx_9:_max_fen,_cdl_mrgn,_form;

{28 mx_10:_2ndry_fossa,_presnc;

{29 mx_11:_aofo,_contact_with_nasal;

{30 mx_12:_lateral_interfen_strut,_length;

{31 mx_13:_interfen_strut_base,_perf;

{32 mx_14:_NEW_interfen_strut_top;

{33 mx_15:_body,_depth;

{34 mx_16:_aofo,_mrgn_below_aofen;

{35 mx_17:_subcutaneous_flange,_presence;

{36 mx_18:_dorsolateral_process,_aofo_covrg;

{37 mx_19:_aofo,_posn_ahead_of_promax_fen;

{38 mx_20:_rostroventral_margin,_profile;

{39 mx_21:_js_for_palatine,_dv_height;

{40 mx_22:_rostral_ramus,_step,_presence;

{41 mx_23:_form_of_nasal_suture;

{42 mx_24:_subcutaneous_surface,_texture_A;

{43 mx_25:_NEW_subcutaneous_texture_B;

{44 mx_26:_rim_between_aofo_&_subcut_srfc;

{45 mx_27:_ascending_ramus,_size;

{46 mx_28:_caudal_region,_shape;

{47 mx_29:_mx_flange,_presence;

{48 mx_30:_primary_foramen_row,_groove;

{49 mx_31:_aofo,_extent_on_main_body;

{50 mx_32:_palatal_shelf,_shape;

{51 mx_33:_dental_pits,_presence;

{52 na_1:_dorsal_surface,_shape;

{53 na_2:_pmx_prs,_separation;

{54 na_3:_midline_crest,_presence;

{55 na_4:_midsection,_texture;

{56 na_5:_midline_rugosities;

{57 na_6:_shape,_dorsal_view;

{58 na_7:_frontal_process,_width;

{59 na_8:_pneumatic_recess,_presence;

{60 na_9:_caudolateral_process,_presence;

{61 na_10:_narial_fossa,_extent_on_pmx_process;

{62 na_11:_medial_frontal_process,_shape;

{63 na_12:_dorsolateral_crest,_presence;

{64 la_1:_shape,_lateral_view;

{65 la_2:_cornual_pr,_presence;

{66 la_3:_cornual_process,_form_A;

{67 la_4:_cornual_process,_form_B;

{68 la_5:_cornual_process,_height;

{69 la_6:_cornual_process,_apex,_position;

{70 la_7:_cornual_process,_inflation;

{71 la_8:_accessory_cornual_process,_prsc;

{72 la_9:_pneumaticity,_extent;

{73 la_10:_rstrl_rms,_lngth_rltv:vntrl_rms;

{74 la_11:_rostral_ramus,_inflation;

{75 la_12:_lpr,_size;

{76 la_13:_lpr,_intersection_with_aofo;

{77 la_14:_rostral_rms,_acc_for,_position;

{78 la_15:_medial_pneumatic_recess_A,_presence;

{79 la_16:_NEW_mdl_pnmtc_recess_B,_position;

{80 la_17:_rostrodorsal_pr,_presence_&_length;

{81 la_18:_supraorb_pr,_size_&_shape;

{82 la_19:_ventral_rms,_suboc_pr,_presence;

{83 la_20:_rostroventral_ala,_height;

{84 la_21:_orbitonasal_ridge,_position;

{85 la_22:_js_for_frontal,_form;

{86 la_23:_frontal_pr,_inflation;

{87 la_24:_rostroventral_ala,_length;

{88 la_25:_mx_pr,_exposure_in_lateral_view;

{89 ju_1:_maxillary_process,_height;

{90 ju_2:_aofo,_edge_on_jugal;

{91 ju_3:_jpr,_rostrocaudal_position;

{92 ju_4:_jpr,_long_axis_angle;

{93 ju_5:_secondary_fossa,_presence;

{94 ju_6:_secondary_fossa,_position;

{95 ju_7:_caudoventral_lac_suture,_angle;

{96 ju_8:_postorbital_process,_fossa_depth;

{97 ju_9:_js_for_postorbital,_form;

{98 ju_10:_js_for_postorbital,_extent;

{99 ju_11:_ridge_caudal_to_po,_presence;

{100 ju_12:_popr,_orientation;

{101 ju_13:_po_pr,_lratio_l:suborbital_height;

{102 ju_14:_ventral_cornual_process,_presence;

{103 ju_15:_lateral_ornamentation,_form;

{104 ju_16:_dorsal_qj_pr,_orientation;

{105 ju_17:_js_qj,_ventral_mrgn,_orientation;

{106 ju_18:_orbit_margin,_shape;

{107 ju_19:_orbit_margin_in_juveniles,_shape;

{108 ju_20:_rim_along_ventral_margin,_presence;

{109 po_1:_cornual_process,_form;

{110 po_2:_cornual_process,_dorsal_extent;

{111 po_3:_cornual_pr,_posn_rltv_to_orbit;

{112 po_4:_cornual_pr,_posn_rltv_to_ltf;

{113 po_5:_sq_pr,_drsl_mrgn,_form;

{114 po_6:_sq_pr,_tip_rltv_to_ltf;

{115 po_7:_subocular_pr,_presence_and_form;

{116 po_8:_subocular_pr,_dv_position;

{117 po_9:_frontal_process,_form;

{118 po_10:_frontal_process,_shape;

{119 po_11:_jugal_ramus,_rostrocaudal_length;

{120 plp_1:_palpebrals,_presence;

{121 sq_1:_dtfo,_lateral_ridge,_division;

{122 sq_2:_dtfo,_dorsl_srfc,_form;

{123 sq_3:_qj_pr,_orientation;

{124 sq_4:_qj_pr,_rstrl_tip,_form;

{125 sq_5:_flange_deep_to_qj,_dv_depth;

{126 sq_6:_cdl_pr,_inflation;

{127 sq_7:_cdl_pr,_length;

{128 sq_8:_flange_above_po_cdl_pr,_presence;

{129 sq_9:_po_pr,_rostral_tip,_position;

{130 sq_10:_pneumaticity,_presence;

{131 qj_1:_contriction_of_ltf;

{132 qj_2:_drsl_pr,_ltrl_srfc,_ridge,_form;

{133 qj_3:_extent_of_jugal_on_jugal_process;

{134 qj_4:_jugal_process,_rostral_tip,_form;

{135 qj_5:_jugal_pr,_posn_rltv_to_ltf;

{136 qj_6:_bone,_curvature;

{137 qj_7:_ventral_qu_pr,_presence;

{138 qj_8:_ventral_qu_pr,_length_&_orientation;

{139 qj9:_NEW_shaft,_pn_for,_presence;

{140 qu_1:_qu_foramen,_size;

{141 qu_2:_quadrate_foramen,_position;

{142 qu_3:_pneumaticity,_presence;

{143 qu_4:_pneumaticity,_caudal_surface,_prsnc;

{144 qu_5:_condyles,_position;

{145 qu_6:_qj_js_on_lateral_condyle,_extent;

{146 qu_7:_q_j_js_on_lateral_condyle,_orientation;

{147 qu_8:_orblt_pr,_medial_fossa,_prsnc_&_depth;

{148 pr_1:_exposure_in_dorsal_view;

{149 pr_2:_ventral_process,_extent;

{150 fr_1:_shape,_dorsal_view;

{151 fr_2:_l:w_ratio;

{152 fr_3:_prefrontolacrimal_process,_length;

{153 fr_4:_nasal_process,_ridge,_presence;

{154 fr_5:_dtfo_l:fr_l;

{155 fr_6:_dtfo,_medial_extent;

{156 fr_7:_sagittal_crest,_form;

{157 fr_8:_sagittal_crest,_length;

{158 fr_9:_postorbital_contact,_form;

{159 fr_10:_contribution_to_the_orbital_fenestra;

{160 fr_11:_forehead,_orientation;

{161 pa_1:_parietofrontal_suture,_form;

{162 pa_2:_parietofrontal_peak,_presence;

{163 pa_3:_sagittal_crest,_presence;

{164 pa_4:_sagittal_crest,_form;

{165 pa_5:_skull_table,_form;

{166 pa_6:_sagittal_crest,_height;

{167 pa_7:_sagittal_crest,_ventral_extent;

{168 pa_8:_nuchal_crest,_height;

{169 pa_9:_nuchal_crest,_cdl_srfc;

{170 pa_10:_interparietal_suture,_fusion;

{171 vo_1:_rostral_process,_shape;

{172 vo_2:_NEW_keel_depth;

{173 ec_1:_body,_inflation,_presence;

{174 ec_2:_jugal_pr,_pneumatic_recess,_presence;

{175 ec_3:_jugal_process,_inflation;

{176 ec_4:_recess,_size_and_shape;

{177 ec_5:_rim_of_recess,_form;

{178 pl_1:_vo-pt_pr,_length;

{179 p1_2:_vo-pt,_neck_orientation;

{180 pl_3:_pneumaticity,_presence;

{181 pl_4:_pn_recesses,_number;

{182 pl_5:_caudal_pn_recess,_position;

{183 pl_6:_caudal_pn_rec,_rstrl_mrgn,_psn;

{184 pl_7:_jugal_pr,_js_for_lc,_psn;

{185 pl_8:_mx_pr,_js_mx,_form;

{186 pl_9:_jgl_pr,_inflation;

{187 pl_10:_lateral_pr,_presence;

{188 pl_11:_brace,_orientation;

{189 palop_1:_choana,_shape;

{190 palop_2:_suborbital_fenestra,_shape;

{191 bc_1:_occipital_surface,_orientation;

{192 bc_2:_soc,_contrib_to_foramen_magnum;

{193 bc_3:_soc,_forked_condition;

{194 bc_4:_soc,_form;

{195 bc_5:_NEW_soc,_ventral_process,_form;

{196 bc_6:_NEW_soc,_pneumaticity;

{197 bc_7:_soc,_cdl_ridge;

{198 bc_8:_otoc,_flange,_presence;

{199 bc_9:_otoc,_paroc_pr,_fossa;

{200 bc_10:_otoc,_mt_strt,_width;

{201 bc_11:_otoc,_fossa_CNs,_depth;

{202 bc_12:_NEW_otoc,_otic_recess,_flange;

{203 bc_13:_NEW_otoc,_ctr,_presence;

{204 bc_14:_boc,_bsl_tbr,_hght;

{205 bc_15:_boc,_bsl_tbr,_vntrl_notch;

{206 bc_16:_boc,_subcond_recess,_depth;

{207 bc_17:_bs,_bpt_pr,_recess,_presence;

{208 bc_18:_bs,_bs_recess,_orientation;

{209 bc_19:_bs,_bs_recess,_inflation;

{210 bc_20,_bs,_bs_recess,_shape;

{211 bc_21,_bs,_bsicr,_shape;

{212 bc_22:_sph_rstrm,_shape;

{213 bc_23: proo,_dtr,_presence;

{214 bc_24:_NEW_proo,_prootic_fossa,_presence;

{215 bc_25:_lat,_antot_crst,_size;

{216 bc_26:_lat,_antot_crst,_form;

{217 bc_27:_lat,_fossa,_presence;

{218 bc_28:_lat,_ridge_of_dtfo;

{219 bc_29:_lat,_vntrltrl_ridge,_presence;

{220 bc_30:_mesethmoid,_presence;

{221 bc_31:_NEW_endocran,_trigeminal_split;

{222 bc_32:_NEW_endocran,_midbrain_flexure;

{223 bc_33:_NEW_endo,_midbrain_peak;

{224 bc_34:_NEW_endocran,_cochlear_duct,_length;

{225 lj_1:_depth_of_dentary_relative_to_jaw_length;

{226 lj_2:_emf_height;

{227 lj_3:_glenoid_position;

{228 dn_1:_position_of_chin_transition_point;

{229 dn_2:_projection_at_chin,_presence;

{230 dn_3:_symphysis,_texture;

{231 dn_4:_js_for_splenial,_texture;

{232 dn_5:_rostral_alveoli,_size;

{233 dn_6:_primary_foramina_row,_groove;

{234 dn_7:_alveolar_margin,_form;

{235 dn_8:_Meckelian_groove,_depth_&_height;

{236 dn_9:_rostral_margin,_angle;

{237 dn_10:_NEW_ventral_bar,_knob;

{238 su_1:_csf,_size;

{239 su_2:_sur_shelf,_form;

{240 su_3:_sur_shelf,_position_&_form;

{241 su_4:_sur_shelf,_orientation;

{242 su_5:_fossa_caudal_to_csf,_depth;

{243 su_6:_coronoid_region,_orientation;

{244 su_7:_fossa,_presence;

{245 su_8:_fossa_below_glenoid,_presence;

{246 su_9:_flange,_length;

{247 an_1:_ventral_margin,_flexure;

{248 ar_1:_muscle_site,_width;

{249 ar_2:_nonarticular_region,_presence;

{250 sp_1:_rostral_mylohyoid_foramen,_size;

{251 sp_2:_js_for_preart,_presence;

{252 pr_1:_ventral_bar,_ridges,_presence;

{253 pr_2:_midshaft_cross_section;

{254 in_1:_shape;

{255 in_2:_notch,_presnce;

{256 dntn_1:_pmx,_mesial_carina,_position;

{257 dntn_2:_pmx,_crown_4,_size;

{258 dntn_3:_lingual_ridge,_presence;

{259 dntn_4:_pmx_teeth,_curvature;

{260 dntn_5:_mx_teeth,_number;

{261 dntn_6:_mx_teeth,_crn_psn;

{262 dntn_7:_dn_teeth,_number;

{263 dntn_8:_lateral_teeth,_form;

{264 dntn_9:_ltrl_teeth,_dntcl_size_ratio;

{265 cv_1:_axis_&_cervs,_ratio_centrum_l:h;

{266 cv_2:_pn_for,_position;

{267 cv_3:_axis,_pn_for,_fossa,_extent;

{268 cv_4:_axis,_ventral_ridge,_presence;

{269 cv_5:_axis,_pn_for,_presence;

{270 cv_6:_axis,_sp_pr,_ant_srfc,_texture;

{271 cv_7:_axis,_spn_pr,_drsl_rgn,_projections,_#;

{272 cv_8:_axis,_spin_pr,_form;

{273 cv_9:_axis,_suprdpsl_fossa,_prsnc_or_form;

{274 cv_10:_axis-mid_cervs,_epipophysis,_form;

{275 cv_11:_axis-mid_cervs,_epi,_form,_B;

{276 cv_12:_mid-post_cervs,_spns_prs,_shape;

{277 cv_13:_spn_prs,_mid-post_cervs,_height;

{278 cv_14:_ant-mid_cervs,_cntrdphsl_lam,_form;

{279 cv_15:_hypapophysis,_presence;

{280 cv_16:_mid_cervs,_prezyg,_position;

{281 cv_17:_cntrdpsl_lmna,_orientation;

{282 dv_1:_crv_&_drs,_lig_scars,_form;

{283 dv_2:_pneumaticity,_extent;

{284 dv_3:_spinous_process,_position;

{285 dv_4:_mid-post,_centra,_length:height;

{286 dv_5:_mid-post,_pstzyg,_position;

{287 dv_6:_mid-post,_cntrdpl_lmna,_form;

{288 sv_1:_centra,_pn_for,_presence;

{289 sv_2:_fenestrae_between_spns_prs;

{290 sv_3:_ribs,_position_on_sacrum;

{291 sv_4:_ribs,_position_on_sacrals;

{292 sv_5:_sacral_five,_pstr_js,_position;

{293 sv_6:_last_scacral,_hyposhene,_form;

{294 cdv_1:_ant_cdls,_sp_pr,_position;

{295 cdv_2:_ant_cdls,_trnsv_prs,_dtl_end,_form;

{296 cdv_3:_ant_cdls,_laminae,_presence;

{297 sc_1:_angle_between_glenoid_and_blade;

{298 sc_2:_acromion_depth_relative_to_min_depth;

{299 sc_3:_ratio_length_to_min_depth;

{300 sc_4:_ratio_of_expansion_to_min_depth;

{301 sc_5:_glenoid,_position;

{302 co_1:_length_relative_to_scapular_acromion;

{303 co_2:_coracoid_foramen,_size/presence;

{304 hu_1:_length_relative_to_that_of_the_femur;

{305 hu_2:_head,_form;

{306 hu_3:_dltpct_cr,_apex,_position;

{307 hu_4:_shaft,_twist,_presence;

{308 hu_5:_additional_muscle_tuber,_presence;

{309 hu_6:_notch_btwn_tbr_&_dlt_cr,_presence;

{310 hu_7:_distal_condyles,_form;

{311 ul_1:_shaft,_form;

{312 ul_2:_distal_js,_form;

{313 c&m_1:_primary_distal_carpal,_form;

{314 c&m_2:_manus,_number_of_digits;

{315 c&m_3:_mtc_I,_medal_distal_condyle,_form;

{316 c&m_4:_mtc_I,_medial_margin,_form;

{317 c&m_5:_ratio_mtc_II_length:mtc_I_length;

{318 c&m_6:_mtc_II,_width_rltv_to_width_mtc_I;

{319 c&m_7:_PH_II-1,_length_rltv_mtc_I;

{320 c&m_8:_unguals,_flexor_tubercle,_form;

{321 c&m_9:_curvature,_degree;

{322 il_1:_length_rltv_to_femur_length;

{323 il_2:_supracetabular_ridge,_presence_&_ornt;

{324 il_3:_ridge_orientation;

{325 il_4:_preacet_pr,_form;

{326 il_5:_drsl_margin_rltv_to_sacral_vertebrae;

{327 il_6:_antitroch,_form;

{328 il_7:_acet_crest,_lateral_extent;

{329 il_8:_NEW_acet,_lateral_exposure;

{330 il_9:_acetabular_crest_on_pubic_ped,_form;

{331 il_10:_peduncles,_lengths;

{332 il_11:_pubic_peduncle,_jnt_srfc,_ornttn;

{333 il_12:_postacet_pr,_vntrl_edge,_form;

{334 il_13:_dorsal_margin,_form;

{335 il_14:_postacet_pr,_form;

{336 il_15:_ratio_l:h_&_shape;

{337 il_16:_lin_ridge,_drsl_extent;

{338 il_17:_lin_ridge,_robustness;

{339 il_18:_brevis_fossa,_orientation;

{340 il_19:_mound,_presence;

{341 il_20:_notch,_width;

{342 il_21:_anterodorsal_notch,_presence;

{343 pu_1:_shaft,_anterior_margin,_form;

{344 pu_2:_pubic_tubercle,_form;

{345 pu_3:_pubic_tubercle,_position;

{346 pu_4:_length_boot_rltv_to_height_pubis;

{347 pu_5:_boot,_length_anterior_ramus;

{348 pu_6:_boot,_position;

{349 pu_7:_orientation_rltv_to_long_axis_of_ilium;

{350 pu_8:_length_of_obturator_plate_rltc_to_shaft;

{351 pu_9:_obturator_notch,_form;

{352 is_1:_ischial_tubercle,_form;

{353 is_2:_distal_end,_form;

{354 is_3:_mdishaft_diameter_rltc_to_pubis;

{355 is_4:_obt_pr,_apex,_position;

{356 is_5:_medial_apron,_position;

{357 is_6:_shaft,_curvature;

{358 fe_1:_ova_scar,_position;

{359 fe_2:_lesser_troch,_h_rltc_to_grtr_tr;

{360 fe_3:_proximal_margin,_form;

{361 fe_4:_head,_fossa,_form;

{362 fe_5:_lesser_trochanter,_lssr_tr,_form;

{363 fe_6:_fourth_trochanter,_position;

{364 fe_7:_lateral_condyle,_shape;

{365 fe_8:_extensor_groove,_form;

{366 fe_9:_mesiodistal_crest,_form;

{367 fe_10:_tibiofibular_crest;

{368 ti_1:_length_relative_to_femur;

{369 ti_2:_lateral_condyle,_ant_pr,_presence;

{370 ti_3:_cnemial_crest_&_prox_condyles,_posn;

{371 ti_4:_ltrl_malls,_extent;

{372 ti_5:_ltrl_mlls,_position;

{373 fi_1:_bipartite_scar,_presence;

{374 as_1:_asc_pr_,_width_base;

{375 as_2:_fossa,_form;

{376 pes_1:_arctometatarsus,_presence;

{377 pes_2:_mtt_III,_distal_medial_expansion;

{378 pes_3:_mtt_III,_distal_platform,_presence;

{379 pes_4:_mtts,_distal_separation;

{380 pes_5:_mtt_II,_js_for_mtt_III,_form;

{381 pes_6:_mtt_II,_prox,_form;

{382 pes_7:_mtt_IV,_distal_l:w;

{383 pes_8:_mtt_IV,_scar,_prsnc_&_form;

{384 pes_9:_prox_pedal_phlgs,_ratio_l:mdshft_w;

{385 pes_10:_lip,_presence;

;

ccode + 7.9 12 18 22 25 30 36 37 51 71 75 80 85 93 97 98 114 118 119 157 179 180 184 190

195 197 200 216 223 238 240 256 269 277 282 284 285 289 304 322 323 332 333 342 345 *;

proc /; comments 1

{24 2 000;

;

**Modified matrix Loewen and colleagues47 with *M*. *intrepidus*.**

xread 501 53

Tawa_hallae

1010001100000000?10010?0110020100???00000100?0000??0?00000?0010????0?00?0

00????00000?001000000000??0???10000?0??0000000?00???0?0010??11000011000000001100

?10000?0?0????000?00000?000000000000000000000????0000??????????????????0??00000?0

2000000?0000010000000???10000?10000?0100??000000010000100000000201000???????????

?00?000000000000000000???00??????0???0000?0??000100100000?000010000?000?00000000

000000000000000?0000200000000000000000????0??0???0000??00000?00000?0000?0000000

0000000000000000000000000000

Coelophysis_bauri

0100001100000000000000?0110000000???00000000?0000??0?0000000010????0?00?0

00????10000?00100000000000000000000?0??0000000?00?????0100??0000001000000000000

0?1000000?0????000?00000000??????0000000000000????00000???00??0?????????0??00000?

02000010?0000000000000??0?0000?1000??0100??0000000000001000000000000000??001010

0000000000000000000000000000000000000000000000000000000000000000100000000?0000

0000000000000000000?00002000000001000?0000?0000000??00000??00000?0000000000?000

00000000000000000000000000000000

Dilophosaurus_wetherilli 00021000000000001100000011?000000???00001010?00?2010???0?0??011????0?00?00

0????00000?00??0?0???????????00000?1????00000?00???0?0110??01000010000000000000?1

000000?0????000?00001000??????0000000000000??????????????????????????0??00000?02??

00110?010000000000??000000?100???0100??0000000000001000000002020000??0010000031

02000100000000000000000000?000001001000000000000000000000000100000000010000000

000100100000010?00002000000011000?0000?0000000??000010001100?0011000000?000100

00000000010000000000000000000

Ceratosaurus_nasicornis

00121000000100001000100000000000200000001010?0011000?110200011210000?10?

001000010000?00101000000001000100000?1??0000000?00???0?0200??100000100000000000

00?0???001010010000?011001000000000000000000000????0000000010000???00000?0??000

00?02010010?0010010000000??100000?0000210100??00000000000021000000120300000000

0000003102000112110001000110000000??00001001000000010000010000000000000000100?

10000000000101100000010?00002000001011000?0000?0000000??0000100011010011100010

1000010010111010020000000000000000000

Dubreuillosaurus_valesdunensis 00121000000000000010001?00?00?00200000001100?00?????????????01?????0?10?00

0???0000100??100000000000000000000?0??0000000?00???0?02?????????00?0???00001000??

?????1??????000?001010100000000000000000100?????000?????????????????00??00000?0??1

?0010?010110000000??110001000??????????????????????1???00000203000?????????????????

????????010000??1??00?000000000110000001??0000000??????????????????????????????????

????????????????????????????????????????0??10?0??0???11??????0?01?????????????????????

????????????

Piatnitzkysaurus_floresi

????????0??0???????????????02110100000001110?0????????????????????????????????

?????????100??000000??????????????????????????????????????????????????????????????????

???000?001??010??????0000000000100?????000???????????????????????????0?????0????00??

0000000??????????????????????????????????????0??01??0000??001100003102000100010001

001000100000001???????????????0000000000000100100000000????????????10010000?010?0

0000000001010100000?0?000000000010010000101001?1011100?01??????????100100001000

01000001000

Eustreptospondylus_oxoniensis

??02100000000000??10000011002110200100?01110?0??????????????021????0?10?00

11000000?????100?0000?000000?10000?1??0110000?00???0?01?????????????????0??10?0?0?

??011???????10?0010001000000000000000001010?000??????????????????????????????0??1?

01100010??0000000?????????????????????????????????10000000????00010100110000301200

010000000111110000000?00100010001000????0?10010000?????00000000??????????????100

000000010?2000100100001010001010?000000???0011100?1?00?11210111110011100001112

10000000000000000000000

Monolophosaurus_jiangi

1102101010000010110000??00100100100100001100?00111110101201201210010?10?

000????1001000011100010?000000001000?1??0110000?0101???0221?1010000001000000011

00?20200?1011000010?000000100000000000011000100?????000?0011000??????????????????

?020100110?010010????00??110001010001100?1100000000??00011000000020100010000010

000101200011210000110101011000??0??00100???????????????????????????????????????????

???10?100000000?00002001012010000?0010?0000000000?????????????????????????????????

?????????????????????????

Sinraptor_dongi

00121000100000101010001000100100100000001010?0000??0?000200112210010?10?

001100001010000111000000000000101000?1??0010001000?????02120101000001000000001

10101000011011110000?011011100000000000110001000????00001001100010?00000001000

000000201000100010010000000001100010000011110110000000000000110000000102000100

00010000301200011211000110011011?00000??00110????????1??11110000?1??00??????????

11???00????100100?01010?0000100101101010000011000000010001111000111101111011111

001010010111010000000100001000001000

Allosaurus_fragilis

00121010000000100000000100111100200000001000?0000??0?000201112210010?10?

001000011010000111000000001000101000?1??0010000?00???0?0201?0010000010000000011

0101000001011110000?011001100000000000110000100????0000000110000??000000010000

0000010000010001011000000000110000?1000111101101000000000010100000011010001100

001000010120001121100011101101100000010001101100101010011110000010100100000100

021010000000100100001010?1000200101101010301010?000000200011110001111011110111

11001110010111010100000100001000001000

Tanycolagreus_topwilsoni 0??21011??0000000?1001000??0????????????????????????????????0?200100?1??0011

000010100?0????????????????11000?1?????0????????????????????0?00?????00001100?20100

0?????????????????????????????????????????????????????????????????????????0????????????

???????????11000??????????????????????020011???0?0?????00????????????0010?010210??00

0000000??00??????0000?000100??0011110000010100000000000121000?00000?????????????

????????????001031?01????????????11010000?11011100110011010210102110101000001000

01000010000

Coelurus_fragilis

?????????????????????????????????????????????????????????????????????????????????

???????????????????????????????????????????????????????????????????????????????????????

?????????????????????????????????????????????????????????????????0????000?0000??01????

??????????????????????????????????????????????0?0????????????00210000000000000000011

????????000000100??????0110?0000??0???000000000????????????????????????????????????0

01103?1010???????????1101000011100110011000?0?1200101101?01?0000100001000000000

Sinosauropteryx_prima

012000010??0001002?010??00?01?0?210000000?0??0?00??0??00????021????0?10?00

0??????01?000?00??????0???????1100?1??0??0?01101000??02?????0?000?0000?0????????0??

?10????????????????????????????????????0????????????????????????????????????00?00000??

000??????00????????????000??0??????0??????100010010000202010????????????0010?001000

0000000001?????????00000011101111?0000010000000100000000000121000010010000000?0

0100?02??20110101101010?01??0000?022001??100?1??????00?1?000?0?122111?1?01012000

0100000000000000

Compsognathus_longipes 0120001100000000010000??00?00?0120000000010??0000??0?00020??021????0?1??00

1000?01001??010001???000101?011100?1??01001011???????0220???0000000000100000?00?

?????00?????????????01?????????00000??????0???????0???0????????????????0000100?00000

000?0000?001???00??00000101?000??0?1?000000000?10010001000000101010000?0???0?002

0?000000??00000001??00000?0000000101010010000111000000?10000000000012110001001

0000000?00100?0????1110??000003010?0?000??01210???100?0?1????00?11000?0012001111

0?1010?000100?00000000000

Juravenator_starki

002000010?010010030110??00?0110110000000010???000??0?0002011011????0?10?0

00????00001?2211001????00?01?011100?1??0100001101000??0220??00000010000100000000

??????????????????????????????????????????????????????10101000??????????????????00?000

00??000?0????00???????????0000??0????????00?0????2000100013??010????????????002??01

?000???1000001???1?0?????0000001010000000121000000?100002?0?011?211000100100000

00?00000?1000210111101????????0?????????????????????????00???00??0???????????1012???

???0????????????

Scipionyx_samniticus

112000010?110000120101?000?0010110000100010???000??0?0002002021????0?10?0

00?????0001?0010001????00?01?011100?1??0100101111010????20??00000010000100000000

?????????????????????01??????????????????????????????010011000???????????000100000?00

000??000?1????00?????00????0000??0????????00?0????0000100003??0101000??0?00000020?

010000??01010000??0100???000000001010??00001000000001100002?0?01112100001001000

0000?02200?100020100110100000???0?000??02210???10?????????0?0?????????????????????

????????????????????

Ornitholestes_hermanni

1110001100000000?110111000100101111000001000?00?0??0?010?00?120????0?1??0

01110?11010000??000000??0101?001000?0??0000000?00?????1210??01110000000000101?00

?20100000??????1???01?1010??????0000?100000?????0?000?1000001??????????1000010??02

000000??00?0??000001??000001???001010?0??0000000?0???1100000022030?0????????????0

0211001100000000000011?????????000000101?11??0??????????????000000010?21???100000

000100?01000?20000011021001?00?101100101101010?111?0?010100110?????0?00?????????

??012000110000100001????

Haplocheirus_sollers

0110011100000010?01010?000100101110000001000?0000??0?1002101121????1010?0

00????001?????100?0?00?00101?000100?1??0010000?00???0?0310??11000000000000000000

?10200?0?11?000????010101000000000000000011010?00?000?1001001??????????100000011

02000000??00?00?????????010001010000?021110000000000200110010000000013??????????

???0????????????000000??????000120???????0?01??0011110001001100000???0000210001?0

0010?00001??20?????????????00?000?20???110??1200?11???????0?????0???????????????????

??1?????1000???????????

Utahraptor_ostrommaysorum 0?????0?0???000113100000???????????????????????????????????????????????????????

??????????????????????????????????????011110000?13?????????????????00000010??????????

???????????????????????????????????????????????????????????????????????????????????????

???????????????????????????????????????????????????0??????????????????11111?111?10010?

1???????????1????0??1??????????????????????????????????????????????????????????????????

???????????????????????????????????01100????1211101102????????????????????100

Deinonychus_antirrhopus 00100000000000011311110000?00101200000001?00?0000??0?1?1001?021????1010?0

00?????0??????????????????????11100?1??0010?011110000?1220??000001100000001000010

????????????????????????????????????????????????????1100?10110?0000?0?100000010020?0

000?0001??0010001???1000???00????0?1?000?10000121011000000?10201010000111000200

201010111012111001011???????2111000011111111010010101001?1?00100000002110000000

1002100101200?12??2001012????????????1?0001231011121010000?1100011000?0012211121

021010??00100000000000100

Linheraptor_exquisitus

0010011000000010?21010?000100?01100000001000?00?0??0?1?0?10?111????1010?0

00????001???????0?0???????000000110?1??0010001111000??1310??10000010000000001001

03120??0????????????????????????????????????????????????????????????????????????02?000

00??011?1??????????????1010000?01111010010????????????0??12021?0???????1??0?0?1110

1110000??00000??????????2110100011111111010?10??1101111002???000021100000001????

????????????????????101000?21?????????????11?2?01??0?????0??100??0?1221112102111?00

00100000000000100

Velociraptor_mongolienesis 0010010000000010021010?000100101100000001000?0000??0?1000110021????1010?0

00???0101?????100?00000000000000110?1??00101011010000?1310??0000000000000000000

1031200?00110100111101000100000000000010000001120000001100111110??00????100000

01102000001000110100?0001??110001010000?02111010010000120011000000120211010000

?110000001010111?00021000000??001100021101000111111110100101011011110000000000

21100000001002000101200?22??0111022101000?21111100001231011121010000?100001100

0?00122111210211120000100000000000100

Archaeopteryx_lithographica 1110010111100000?01000?000001101201000000100?0000??0?1002000010????0?10?0

00???0001?????100?1???000001?00??00?0???100????00?????03120?00000000000000000000?

??????000????0111?01110000000001?00?10?0??00???00?00010011010??000????1000000110

00?000100000?00010000??000000?10000??0?0??00010000?0??210000002303110?????111??0

00011?00010000000000000?0001??021100000101111110100101011011110110000000211000

00000000000101200?22??0111022101000?21111100001200011?1111?000?1000??100??00122

111?10211120000101000000000000

Pelecanimimus_polydon

000000??0?001011?3?112??00000?011????000100???000??0?0002001100????20?0?00

0????11001?221?001????4?101?000100???????0????0??????02120000000?000000???????????

??????0??????11?0011?????????01????????????????????1101010???????????????????0200010

0??00011????101???????0??00?1000????010?????????3????0?12000023??????0??000?0?1?0?0

100?000?0000?0???????????????????????011???0000211100002???01??21020002020????????

???????????????????????????????????????????????????????????????????????????????????????

?????????

Shenzhousaurus_orientalis 000000??00001001031110??00000?0111000000100???000??0?100200?100????2010?0

00????11???????00???????0101????????????????10?00?????021200000?0??000?0????????????

??????????????????????????????????????????????????1101010????0??????????????02???100??

00?11?????01???????????001000?0??010???????????????????13113?????????????????????????

00000001?11001??1000?1000?0001????????????????????????????21???0??020000011?01201?

01001?11000010010010?0?000?00200111?110011???211??????????????????????????????????

?????????

Harpymimus_okladnikovi 000000??00001001031110??00000?0??????000100???000??0?1002001100????20?0?00

0?????1001?2210001????00101?000100?0??0100110?00?????12????????????????????????????

????????????????????????????????????????????????????????????????????????????02000110?0

0001?????101??000000??00???00?0??010?00?0????3????1????130??????????????0001100010

0?001000001011001???0001?00000?010?0110000010??11000021???1?12101100202000?011?

0?201011001???0?201??1???0110??????????111????????????????01????01111021021012????

20100?????00001

Gallimimus_bullatus

0010001?000010110?0112??00000?0111000000100???000??0?1002001100????2010?0

00?????1001?2001001????40101?000100?0??0100110?00?????121????0?00?000000?????????

10?????00????0111?0011000??????0111000110?01??00?????1101010???????????????????020

00110?000011????1?1??000000??0001000?0??010100?002??3????1????13???10?????1????00?

11000100?01100100001100100?00010000000011101111??0102111000020??00??2102000202

000?01110200101100100100201001001011000010020111??0?????122221?111????0???1????1

?2?012????21200?????00001

Proceratosaurus_bradleyi 22000011000?11110311111?00101101100000002200?0?01??0?????????????????1?????

????0???0????????????????????0000?????????????1000???2220001000020010101001100?100

00?0????????????????????????????????0??????01??0?01001000????????0?10000????02000000

??110010???110???????1110011000?1000000000?0201121000000001010???????????????????

???????????????????????????????????????????????????????????????????????????????????????

???????????????????????????????????????????????????????????????????????????????????????

?????

Kileskus_aristotocus

220??????????111?31111?000?02101201000002200?0???????????????????????????????

???????????????????????????????????????????????????????????????????????????????????????

???????????????????????????????????????????????????????????????????????????????????????

????????????????????1110010??????????12???0??01???00??????????????????????????????????

???????????????????????????????????????????????????????????????????????????????????????

???????????????????????????????????????????????????????01?????10000??????1000

Guanlong_wucaii

2200001100001111?311111000102101200000002200?0011100?0010011011????0?10?0

01110001000?00100000000001000010000?1??0010?00?010100?122200010000200101010011

00?????0?001100020111010101100000001100000001111001010001?0?000??????????1000000

0?02?000010?110?100????0???????11100???00?10000000000????1210000011??00010000001

0000000100000000000000000110001001?0000?00?010??100110000000?01000000000000210

001000000000000100020000010110120002020101100011010100111100001110111001110100

0021111210110120010100000000001000

Sinotyrannus_kazuoensis 12??????????1111?3?111??001011??10100000??00?????????????????????????????????

???????????????????????????????????????????????????????????????????????????????????????

???????????????????????????????????????????????????????????????????????????0???0???????

???0?????????????????????????????????????0010????000?????????????????????????110001?0

??????????????????????????????????????????????????????????00010?1??????1200000101101?

????????????????????????????????????????????????????????????????????????????????

Juratyrant_langhami

?????????????????????????????????????????????????????????????????????????????????

???????????????????????????????????????????????????????????????????????????????????????

???????????????????????????????????????????????????????????????????????????????????????

????????????????????????????????????????????????????????????000000100100010000000010

00?001??00000??0101???????????????????????????????????????1001000?10110000000110120

0??0??10110?011111100?????????110011001111?????????????1???????????????????????

Stokesosaurus_clevelandi

?????????????????????????????????????????????????????????????????????????????????

???????????????????????????????????????????????????????????????????????????????????????

???????????????????????????????????????????????????????????????????????????????????????

???????????????????????????????????????????????????????????????????????????????????????

???????????????????????????????????????????????????????10?10?010011100001011012??????

?????????????????????????????????????????????????????????????????????????? Dilong_paradoxus 11000011000011111310011100100101211000001100?001[1

2]100?001001101200100?10?001110001000?00100000000001000010000?1??0010100?010110

?12220001100020010001001100?20001?00100102010101000010000000110000000110000101

00??????????????????10000000?011000010001001000001000111001110011001110010000000

0211121100002203000100000010010000000100?000?00000001?????????00000000010101000

1001000010100100000000?210011000001001?????0???1000??????2010001?1010?000??1210?

110100001110011001110??0?021????????0110000100000000000000

Eotyrannus_lengi

????????????1?1????001?000?0010?1?10???0??0???010??1010100110?200????1??1011

10000??0??????????????????????????????????????????????????????????????????????20111???

??????????????????????????????????????????00???????????????????????????0????00??00?0??

0000010???????????0????1111000??????????132??0?0????000????????00????0??0???????2???

????????????????????????????0?111??0000101000011001??0?10?????000???????????10?????0

???????????????????????????????????????????????????????????????01?00001000000000?0000

Dryptosaurus_aquilunguis

???????????????????????000?0???????????0????????????????????????????????????????

???????????????????????????????????????????????????????????????????????????????????????

??????????????????????????????????????????????????????????????????0?????????0????00????

0????????????????1211010???????????????0?1????000?????????????????????????????????????

?????????????00?????????????????????10100?1???????????120????????????????????????02???

???010??10??????121?10000?11111?1?111111102221112101?01??????01?1000000????

Xiongguanlong_baimoensis 0000001100011110?1?001?000100?0?1?000?10111101010??0?001001201200??0??0??

0???0?0001?1??001000101111000210000?1???010000?01011??1222000000011000000201110

102000??00110111????01000?0??????01100001001000001?1?00?????????????????100000001

???????????????????????????????????????????????????????1321010121??01010000010001110

100010020012110010011???????????????????????????????????????????????????????????00??

11????0201111?????????????????????????????210101001111221???????????22??????????????

????????????????

Alectrosaurus_olseni

?????????????????????????????????????????????????????????????????????????????????

???????????????????????????????????????????????????????????????????????????????????????

???????????????????????????????????????????????????????????????????????????????????????

???????????????????????????????????????????????????????????????????????????????????????

???????????????????????????????????????????????????????????????????????????????????????

?????????????????????020112210011001110222111210110100100112010110011000

Appalachiosaurus_montgomeriensis 000???????01????0???????00?001011?0000101110??010??0?1?1????0120110?0210001

1100?0????????????????????????????????????????????????22???????????????21111010??????

???????????????????????????????????????????100?100100011?000001010000100?111??00100

0110??100110??11100???????????????????????????????0??1102000????????????????????????

??????????????????????????????????0??????????????1100??11?????????????????????????????

????????0201?1?10111010??1201121?1000121212211111111111222111210110100101112020

111011010

Alioramus_altai

000110110001????0???????0010110120000110111101010??1110100010121121112111

1111000001?121001000101112101210000?1??001010101100121212210010101101100031111

1102011111011021101010110110000110111000100111112110100?1011001211111001020101

0101011010010001101110011000111001111011101121110001011?????????0??210100011011

01010111010001102101211?1100110112100???101?0?0???11??????????????????????????????

???????00?0111????201111????0?1??????????????0111201121210101?122111????00???12221

11210110??1??1112?20?????0010

Alioramus_remotus

00011011000?????????????0010??0???????10111101010??1110100010?21121112??111

1100?001?1??001000101112101210000?1??0110101011001212122????0101101100031111110

201111101102110101011011000011011100010011111211?100?1011?0021001????020101010

10110100100011011100110001110011110111011211100010??1211?????0?1?101000?????????

???????????????????????????????????????????????????????????????????????????????????????

?????????????????????????????????????????????????????????????????????????????????1101??

11?020????0????

Gorgosaurus_libratus

0011101110011110111001100010011110000010102102010??101010001012112021210

01100000001?111011000111112101201012020011100010021101?11221000120100110003111

111020111?10110211010101101100001100110001010100120101001101111011?11101111000

1010111100001000111111101100?1110011110111022211101010111211132100012102001110

1?010?11200200110221112111111011011???1100100000010111001210100000010001110121

1122111100120010011012202011111111011020202010111010111212121?10101?1221110111

112111222111210110221111112120110210010

Albertosaurus_sarcophagus 0011101110011110111001100010011110000010102102010??101010001012112021210

01110000001?111011000211112101201012020011100010021101?11221001120100110003111

111020111110111211010101101100001100110001011100110101001101111011?11101111010

1010111100001000111111101100?1110011110111022211101010111211132100012102001110

11010111?002001112211121111110110111101100100000010111001210100000010001110121

112211110012001001101220201111111101102020201011101011121212121010111221110111

112111222111210110221111112120111210010

DPF_tyrannosaurid_B

00121011100122101110011100100111200000101021?2110??101211001102112121211

11210100001?111012010311212202201011030110101010021101?11221100020100110003121

121120111110100111010101112101111111120001001111120111??1101111021111111112111

101011??010?1000?1111110110??11110????011102221210??????12111321??111101000?????

???????????????????????????????????????????????????????????????????????????????????????

???????????????????????????????????????????????????????????????????????????????????????

???????????????????

Daspletosaurus_torosus

0012101110012210011001??0010011120010011102102110??101211011112112021211

01211111001?111012000211212202201011131110101010021112?21221101120100110013121

121120111?111111?1???????????111111012100100?1111101110011011000210111111021101

010?11101001100111111???1000?????1111111102221110101011121113210010110200111010

01011120?200111221112111011011??????110011100001011100122010000001000111112111

2211110112001111101210201111?111111020213110121010111212121?10101?122??2111111

2111221111210110221111112120??0211010

Daspletosaurus_horneri

0002101110012210?11001??001001112101001110210??10??1012100?1122111021210

01110011001?1??011000211212202201011030010101010021102121221101120100110013121

1211201111111112210101011121011?111012?0010?111??????1??111110101??11?111?21111

110?1110100111011111100110??11110111101110222111010101?1211132100101102001?????

???????0??0?1??????????????????????????0?1??0?010111??122010000?0100????????????????

?????011?1??1210201111?1111110202?3110121010111212121?10101???????0111???????????

??????020111111212011??11010

Teratophoneus_curriei

0?1211111011????????????0010011111000011102102??????????00?102211202121011

120001001?111112001312212302201012130110101010121?12011????00??01??110013121121

1201112111112210111011021001111111110011011101102?1??????????????1????02???110?1

1110100110011111110020???????111111220222222000111?1211?????0?01203001??????????

??00200111321112110111011011?11110011100001011100132??0000?0100012111211???????

?????0121110???02?1111111111102021211012101011121212121010111222220111112111?21

1?1???????????????????????1010

Bistahieversor_sealeyi

0012111111112210011002110010012110000011102102110??101210011022111121210

1?220101001?111112001312212302201012030110100010?2111?011221101121101110013121

101120111211111221010?01102100011111121001111110110211???111101010?111?1102000

0100?11101001110111111100200?0111011111122022222201011111211132100102203001???

?????????0020011132111?????????????????10011200001011???1320100????100?????????????

??????????????????????????????????????????????????????21210101?122?22011111211122111

1210110201111112120111110010

Lythronax_argestes

001212??211?????0???????0000012111000021102102110??101210011???????????????

????????????112101312212????????21?????1??????????????2211111211011100???????112011

121??????????????????????????????????????????1??11??????201?11111??????????111?10011

1011111111020???????1111112202222220102011?????????0??2203002?????????????????????

???????????????????????????????????????????????????????????????????????????????????????

??????????020213110121??????????????????????????111112111?221?1?????02?11111121201

1111????

Tyrannosaurus_rex

0012121121112210011002110000012131111021102102110??101011011122111122211

11220101101?121112101312302202201012131110110010121112020221111121101111013121

121120111011111221011101102100011111111001101110110211112111101120111111112111

111011111100111011111111020110111111111122022222201021111211132100102203002111

110101112002001113211121100110110112111000112000010111001320100000010001211121

112211110112001211101210201111111111102021411012101011121112121010111222221111

112111221111210110221111112120111112010

Tarbosaurus_bataar

0012121121112210011002110000012131111021102112110??101011011122111122211

01220111101?121112101312302202201012131110110010121112120221111121101111013121

121120111011111221011101102100011111111001111110120211112111101120111111112111

111011111100111011111111020110111111111122022221201020111211132100101203002111

110101112002001113211121100110??011?111000112000010111001320100000010001211121

112211110112001111101210201111111111102021411012101011121112121010111222221111

112111221111210110221111112120111112010

Zhuchengtyrannus_magnus

?????????1???????1??????000001213?111021102112????????????????????????????????

???????????????????????????????????????????????????????????????????????????????????????

??????????????????????????????????????1?????????????????????????????11111001110111111

11020??????????????????????????????????????0??1203002?????????????????????????????????

???????????????????????????????????????????????????????????????????????????????????????

??????????????????????????????????????????????????????????????????????????????

Moros_intrepidus

?????????????????????????????????????????????????????????????????????????????????

???????????????????????????????????????????????????????????????????????????????????????

???????????????????????????????????????????????????????????????????????????????????????

???????????????????????????????????????????????????????????????????????????????????????

???????????????????????????????????????????????????????????????????????????????????????

?????????????????10?00111????0?11???????[1 2]21?1??????[1 2]?0100??2??011101????

;

proc /; comments 0

;

# Supplementary References

- 1. Yingling, V. L., & Heller, P. A. U. L. (1992). Timing and record of foreland sedimentation during the initiation of the Sevier orogenic belt in central Utah. *Basin Research*, *4*(3-4), 279-290.
  2. Lawton, T. F., Talling, P. J., Hobbs, R. S., Trexler Jr, J. H., Weiss, M. P., & Burbank, D. W. (1993). *Structure and stratigraphy of Upper Cretaceous and Paleogene strata (North Horn Formation), eastern San Pitch Mountains, Utah; sedimentation at the front of the Sevier orogenic belt* (No. 1787-II).
  3. DeCelles, P. G., Lawton, T. F., & Mitra, G. (1995). Thrust timing, growth of structural culminations, and synorogenic sedimentation in the type Sevier orogenic belt, western United States. *Geology*, *23*(8), 699- 702.
  4. DeCelles, P. G., & Currie, B. S. (1996). Long-term sediment accumulation in the Middle Jurassic–early Eocene Cordilleran retroarc foreland-basin system. *Geology*, *24*(7), 591-594.
  5. Currie, B. S. (1998). Upper Jurassic-Lower Cretaceous Morrison and Cedar Mountain Formations, Ne Utah-Nw Colordao: Relationships between Nonmarine Deposition and Early Cordilleran Foreland-Basin Development. *Journal of Sedimentary Research*, *68*(4), 632-652.
  6. Greenhalgh, B. W., & Britt, B. B. (2007). Stratigraphy and sedimentology of the Morrison–Cedar Mountain Formation boundary, east-central Utah, *Central Utah: Diverse Geology of a Dynamic Landscape*, 81-100.
  7. Suarez, C. A., González, L. A., Ludvigson, G. A., Cifelli, R. L., & Tremain, E. (2012). Water utilization of the Cretaceous Mussentuchit Member local vertebrate fauna, Cedar Mountain Formation, Utah, USA: using oxygen isotopic composition of phosphate. *Palaeogeography, Palaeoclimatology, Palaeoecology*, *313*, 78- 92.
  8. Roca, X., & Nadon, G. C. (2007). Tectonic control on the sequence stratigraphy of nonmarine retroarc foreland basin fills: Insights from the Upper Jurassic of central Utah, USA. *Journal of Sedimentary Research*, *77*(3), 239-255.
  9. Stokes, W. L. (1952). Lower Cretaceous in Colorado Plateau. *AAPG Bulletin*, *36*(9), 1766-1776.
  10. Hunt, G. J., Lawton, T. F., Kirkland, J. I., Sprinkel, D. A., Yonkee, W. A., & Chidsey, T. C. (2011). Detrital zircon U-Pb geochronological provenance of Lower Cretaceous strata, foreland basin, Utah. *Sevier Thrust Belt: Northern and Central Utah and Adjacent Areas: Utah Geological Association, Publication*, *40*, 193-211.
  11. Cifelli, R. L., Cohen, J. E., & Davis, B. M. (2016). New tribosphenic mammals from the Mussentuchit Local Fauna (Cedar Mountain Formation, Cenomanian), Utah, USA. *Palaeontologia Polonica*, *67*, 67-81.
  12. Kirkland, J. I., Simpson, E. L., DeBLIEUX, D. D., Madsen, S. K., Bogner, E., & Tibert, N. E. (2016). Depositional constraints on the Lower Cretaceous stikes quarry dinosaur site: Upper yellow cat member, cedar mountain formation, Utah. *Palaios*, *31*(9), 421-439.
  13. Bhattacharya, J. P., & MacEachern, J. A. (2009). Hyperpycnal rivers and prodeltaic shelves in the Cretaceous seaway of North America. *Journal of Sedimentary Research*, *79*(4), 184-209.
  14. Suarez, M. B., Suarez, C. A., Al-Suwaidi, A. H., Hatzell, G., Kirkland, J. I., Salazar-Verdin, J., Ludvigson, G.A., Joeckel, R. M. (2017). Terrestrial Carbon Isotope Chemostratigraphy in the Yellow Cat Member of the Cedar Mountain Formation: Complications and Pitfalls. (In) Terrestrial Depositional Systems (pp. 303-

336); Zeigler, K. E., & Parker, W. (Eds.). (2017). Terrestrial Depositional Systems: Deciphering Complexities Through Multiple Stratigraphic Methods. Elsevier

- 1. Tucker, R.T., Makovicky, P.J., Zanno, L.E. (In Prep) Recent advances in temporal calibration for newly discovered dinosaurian assemblages in the Mussentuchit Member of the Cedar Mountain Formation, Central Utah, USA
  2. W.G.E. Caldwell, E.G. Kauffman (Eds.), Evolution of the Western Interior Basin, Geological Association of Canada, Special Paper, 39 (1993), pp. 1-30
  3. Fielding, C. R. (2011). Foreland basin structural growth recorded in the Turonian Ferron Sandstone of the Western Interior Seaway Basin, USA. *Geology*, *39*(12), 1107-1110.
  4. Ullah, M. S., Bhattacharya, J. P., & Dupre, W. R. (2015). Confluence Scours Versus Incised Valleys: Examples From the Cretaceous Ferron Notom Delta, Southeastern Utah. *Journal of Sedimentary Research*, *85*(5), 445-458.
  5. Tucker, R. T., Roberts, E. M., Hu, Y., Kemp, A. I., & Salisbury, S. W. (2013). Detrital zircon age constraints for the Winton Formation, Queensland: contextualizing Australia's Late Cretaceous dinosaur faunas. *Gondwana Research*, 24(2), 767-779.
  6. Hendrickx, C., Mateus, O., & Araújo, R. (2015). A proposed terminology of theropod teeth (Dinosauria, Saurischia). *Journal of Vertebrate Paleontology*, *35*(5), e982797.
  7. Campione, N. E., Evans, D. C., Brown, C. M., & Carrano, M. T. (2014). Body mass estimation in

non-avian bipeds using a theoretical conversion to quadruped stylopodial proportions. *Methods in Ecology and Evolution*, *5*(9), 913-923.

- 1. Anderson, J. F., Hall-Martin, A., & Russell, D. A. (1985). Long-bone circumference and weight in mammals, birds and dinosaurs. *Journal of Zoology*, *207*(1), 53-61.
  2. Christiansen, P., & Fariña, R. A. (2004). Mass prediction in theropod dinosaurs. *Historical Biology*, *16*(2- 4), 85-92.
  3. Campbell Jr, K. E., & Marcus, L. (1992). The relationship of hindlimb bone dimensions to body weight in birds. *Natural History Museum of Los Angeles County Science Series*, *36*, 395-412.
  4. Averianov, A. O., Sues, H. D., Dyke, G., & Bayshashov, B. (2017). Hind limb bones of an ornithomimid dinosaur from the Upper Cretaceous Bostobe Formation, northeastern Aral Sea region,

Kazakhstan. *Palaeoworld*, *26*(1), 194-201.

- 1. Madsen Jr, J. H. (1976). *Allosaurus fragilis*: a revised osteology. *Utah Geological and Mining Survey Bulletin*, *109*, 1-163.
  2. Brochu, C. A. (2003). Osteology of *Tyrannosaurus rex*: insights from a nearly complete skeleton and high- resolution computed tomographic analysis of the skull. *Journal of Vertebrate Paleontology*, *22*(sup4), 1- 138.
  3. Brusatte, S. L., Carr, T. D., & Norell, M. A. (2012). The osteology of *Alioramus*, a gracile and long- snouted tyrannosaurid (Dinosauria: Theropoda) from the Late Cretaceous of Mongolia. *Bulletin of the American Museum of Natural History*, 1-197.
  4. Sues, H. D., & Averianov, A. (2016). Ornithomimidae (Dinosauria: Theropoda) from the Bissekty Formation (Upper Cretaceous: Turonian) of Uzbekistan. *Cretaceous Research*, *57*, 90-110.
  5. Madsen, J. H., & Welles, S. P. (2000). *Ceratosaurus (Dinosauria, Theropoda): a revised osteology*. Utah Geological Survey.
  6. Balanoff A, Norell M (2012) Osteology of *Khaan mckennai* (Oviraptorosauria, Theropoda). Bulletin of the American Museum of Natural History: 372.
  7. Funston, G. F., & Currie, P. J. (2018). A small caenagnathid tibia from the Horseshoe Canyon Formation (Maastrichtian): Implications for growth and lifestyle in oviraptorosaurs. *Cretaceous Research*, *92*, 220- 230.
  8. Carpenter, K. (2005). Redescription of the small maniraptoran theropods *Ornitholestes* and *Coelurus* from the Upper Jurassic Morrison Formation of Wyoming. In *Carnivorous Dinosaurs* (p. 49).
  9. Xu, X., Norell, M. A., Kuang, X., Wang, X., Zhao, Q., & Jia, C. (2004). Basal tyrannosauroids from China and evidence for protofeathers in tyrannosauroids. *Nature*, *431*(7009), 680.
  10. Persons IV, W. S., & Currie, P. J. (2016). An approach to scoring cursorial limb proportions in carnivorous dinosaurs and an attempt to account for allometry. *Scientific reports*, *6*, 19828.
  11. Xu, X., Wang, K., Zhang, K., Ma, Q., Xing, L., Sullivan, C., ... & Wang, S. (2012). A gigantic feathered dinosaur from the Lower Cretaceous of China. *Nature*, *484*(7392), 92.
  12. Carr TD, Varricchio DJ, Sedlmayr JC, Roberts EM, Moore JR. 2017. A new tyrannosaur with evidence for anagenesis and crocodile-like facial sensory system. Scientific Reports 7:44942 DOI: 10.1038/srep44942
  13. Osmólska, H. A. L. S. Z. K. A., Roniewicz, E., & Barsbold, R. (1972). A new dinosaur, *Gallimimus bullatus* n. gen., n. sp. (Ornithomimidae) from the Upper Cretaceous of Mongolia. *Palaeontologia Polonica*, *27*, 103-143.
  14. Kobayashi, Y., & Barsbold, R. (2005). Reexamination of a primitive ornithomimosaur, *Garudimimus brevipes* Barsbold, 1981 (Dinosauria: Theropoda), from the Late Cretaceous of Mongolia. *Canadian Journal of Earth Sciences*, *42*(9), 1501-1521.
  15. Fowler, D. W. (2016). A new correlation of the Cretaceous formations of the Western Interior of the United States, I: Santonian-Maastrichtian formations and dinosaur biostratigraphy. *PeerJ Preprints*, *4*, e2554v1.
  16. Carr, T. D., Williamson, T. E., & Schwimmer, D. R. (2005). A new genus and species of tyrannosauroid from the Late Cretaceous (Middle Campanian) Demopolis Formation of Alabama. *Journal of vertebrate Paleontology*, *25*(1), 119-143.
  17. Carpenter, K., Russell, D., Baird, D., & Denton, R. (1997). Redescription of the holotype of *Dryptosaurus aquilunguis* (Dinosauria: Theropoda) from the Upper Cretaceous of New Jersey. *Journal of vertebrate Paleontology*, *17*(3), 561-573.
  18. Trujillo, K. C., Foster, J. R., & Lucas, S. G. (2006). Clay mineralogy of the Morrison Formation (Upper Jurassic-? Lower Cretaceous), and its use in long distance correlation and paleoenvironmental

analysis. *New Mexico Museum of Natural History and Science Bulletin*, *36*, 17-23.

- 1. Xu, X., Clark, J. M., Forster, C. A., Norell, M. A., Erickson, G. M., Eberth, D. A., ... & Zhao, Q. (2006). A basal tyrannosauroid dinosaur from the Late Jurassic of China. *Nature*, *439*(7077), 715.
  2. Averianov, A. O. (2002). An ankylosaurid (Ornithischia: Ankylosauria) braincase from the Upper Cretaceous Bissekty Formation of Uzbekistan. *Bulletin-Institut royal des sciences naturelles de Belgique. Sciences de la terre*, *72*, 97-109.
  3. Brusatte, S. L., & Benson, R. B. (2013). The systematics of Late Jurassic tyrannosauroid theropods from Europe and North America. *Acta Palaeontologica Polonica*, *58*(1), 47-54.
  4. Loewen, M. A., Irmis, R. B., Sertich, J. J., Currie, P. J., & Sampson, S. D. (2013). Tyrant dinosaur evolution tracks the rise and fall of Late Cretaceous oceans. *PloS one*, *8*(11), e79420.
  5. Li, D., Norell, M. A., Gao, K. Q., Smith, N. D., & Makovicky, P. J. (2009). A longirostrine tyrannosauroid from the Early Cretaceous of China. *Proceedings of the Royal Society of London B: Biological Sciences*, rspb-2009.
  6. Brusatte, S. L., Averianov, A., Sues, H.-D. & Butler, I. B. (2016) New tyrannosaur from the mid- Cretaceous of Uzbekistan clarifies the evolution of giant body sizes and advanced senses in tyrant dinosaurs. Proc. Nat. Acad. Sci. USA 113(13), 3447, doi: 10.1073/0nas.1600140113.
