# Supplementary material for: Diminutive fleet-footed tyrannosauroid narrows the 70-million-year gap in the North American fossil record
Source: Commun Biol. 2019 Feb 21;2:64. doi: 10.1038/s42003-019-0308-7 (PMC6385174; doi:10.1038/s42003-019-0308-7)
Supplement: Supplementary file 2 — Reporting Summary [file 42003_2019_308_MOESM2_ESM.pdf]

nature research

Corresponding author(s): Lindsay Zanno

Reporting Summary

Nature Research wishes to improve the reproducibility of the work that we publish. This form provides structure for consistency and transparency in reporting. For further information on Nature Research policies, see Authors & Referees and the Editorial Policy Checklist.

Statistical parameters

When statistical analyses are reported, confirm that the following items are present in the relevant location (e.g. figure legend, table legend, main text, or Methods section).

n/a	Confirmed	
☐	☒	The exact sample size (*n*) for each experimental group/condition, given as a discrete number and unit of measurement
☐	☒	An indication of whether measurements were taken from distinct samples or whether the same sample was measured repeatedly
☒	☐	The statistical test(s) used AND whether they are one- or two-sided<br>*Only common tests should be described solely by name; describe more complex techniques in the Methods section.*
☒	☐	A description of all covariates tested
☒	☐	A description of any assumptions or corrections, such as tests of normality and adjustment for multiple comparisons
☐	☒	A full description of the statistics including central tendency (e.g. means) or other basic estimates (e.g. regression coefficient) AND variation (e.g. standard deviation) or associated estimates of uncertainty (e.g. confidence intervals)
☒	☐	For null hypothesis testing, the test statistic (e.g. *F*, *t*, *r*) with confidence intervals, effect sizes, degrees of freedom and *P* value noted<br>*Give P values as exact values whenever suitable.*
☒	☐	For Bayesian analysis, information on the choice of priors and Markov chain Monte Carlo settings
☒	☐	For hierarchical and complex designs, identification of the appropriate level for tests and full reporting of outcomes
☒	☐	Estimates of effect sizes (e.g. Cohen's *d*, Pearson's *r*), indicating how they were calculated
☒	☐	Clearly defined error bars<br>*State explicitly what error bars represent (e.g. SD, SE, CI)*

Our web collection on statistics for biologists may be useful.

Software and code

Policy information about availability of computer code

Data collection	no software was used
Data analysis	MASSTIMATE package (version 1.3) in R version 3.3.3; phylogenetic analyses were carried out using Mesquite ver.3.51 58 . Phylogenetic analyses were executed in the program TNT.

For manuscripts utilizing custom algorithms or software that are central to the research but not yet described in published literature, software must be made available to editors/reviewers upon request. We strongly encourage code deposition in a community repository (e.g. GitHub). See the Nature Research guidelines for submitting code & software for further information.

Data

Policy information about availability of data

All manuscripts must include a data availability statement. This statement should provide the following information, where applicable:

- Accession codes, unique identifiers, or web links for publicly available datasets
- A list of figures that have associated raw data
- A description of any restrictions on data availability

The authors declare that all measurement and phylogenetic data supporting the findings of this study are available within the paper (and its supplementary

information files) and that paleontological specimens including paleohistological sections generated and analyzed during the current study are available for access to qualified researchers at the NC Museum of Natural Sciences by request.

Field-specific reporting

Please select the best fit for your research. If you are not sure, read the appropriate sections before making your selection.

☐ Life sciences ☐ Behavioural & social sciences ☒ Ecological, evolutionary & environmental sciences

For a reference copy of the document with all sections, see nature.com/authors/policies/ReportingSummary-flat.pdf

Ecological, evolutionary & environmental sciences study design

All studies must disclose on these points even when the disclosure is negative.

Study description	Anatomical description, taxonomic identification, and phylogenetic analysis of a new species of extinct dinosaur. Including comparative examination of hind limb length ratios.
Research sample	Three fossil specimens collected in nearby localities, plus comparative fossil material reposited in collections at other public institutions. Paleohistological sections were also made of the holotype specimen. Characterizations of comparative anatomy of related taxa was compiled and published by other authors for the purposes of phylogenetic analyses.
Sampling strategy	Sample was chosen as all representatives of tyrannosauroid fossils from the Mussentuchit Member of the Cedar Mountain Formation collected by the authors; plus comparative fossil specimens of ornithomimosaurs and tyrannosaurs from other public institutions. For phylogenetic analyses, three independent previously published phylogenetic datasets were utilized.
Data collection	Measurements taken with digital callipers and trait characterizations were assessed by the senior author (Zanno).
Timing and spatial scale	Measurements were taking on comparative materials between September 2017 and May 2018 at the NC Museum of Natural Sciences and the Royal Ontario Museum. Duration of specimen measurement was dictated by specimen and researcher availability. Phylogenetic data was compiled between January and April of 2018.
Data exclusions	No data were excluded from the analyses
Reproducibility	All data are provided in this paper and its supplementary information and all specimens are reposited in public institutions where they are available to researchers for reproducibility.
Randomization	Measurement data on specimen dimensions does not require randomization. Support values were generated for phylogenetic analyses to aid in assessing the strength of results. No randomization of phylogenetic data was conducted.
Blinding	Binding is not relevant to phylogenetic analyses or measurement biplots.

Did the study involve field work? ☒ Yes ☐ No

Field work, collection and transport

Field conditions	desert environment in central Utah
Location	exact locality information in on file to available researchers with the North Carolina Museum of Natural Sciences, but may not be disclosed publicly under condition of the collection permit.
Access and import/export	specimens were collected under permit by the State of Utah, permit # Utah2013-422 in compliance with all permit regulations
Disturbance	after collection the site was remediated following permit regulations

Reporting for specific materials, systems and methods

Materials & experimental systems

n/a	Involved in the study
☒	☐ Unique biological materials
☒	☐ Antibodies
☒	☐ Eukaryotic cell lines
☐	☒ Palaeontology
☒	☐ Animals and other organisms
☒	☐ Human research participants

Methods

n/a	Involved in the study
☒	☐ ChIP-seq
☒	☐ Flow cytometry
☒	☐ MRI-based neuroimaging

Palaeontology

Specimen provenance

State of Utah; permit # Utah 2013-422

Specimen deposition

North Carolina Museum of Natural Sciences

Dating methods

Sample preparation was conducted at the Central Analytical Facility, Stellenbosch University, Stellenbosch, South Africa. Samples were crushed and milled in a tungsten carbide disc mill and then sieved using both 250 and 500 µm meshes, washed and decanted numerous times to remove the clay-sized fraction, and lights were separated using Tetrabromoethane (TBE) with a density of 2.96. Mineral separates were then washed, dried, sorted via a Frantz magnetic separator at progressively higher magnetic currents, and non-magnetic heavy mineral separates were then handpicking as randomly as possible from the greater population within a defined field of view. For each sample, ±150 grains were mounted, polished and documented (via a Zeiss MERLIN Field Emission Gun Scanning Electron Microscope) to access microstructures, cracks, inclusions, and other complexities.
Laser ablation U-Pb data were collected at the Central Analytical Facilities, Stellenbosch University, using a 193 nm wavelength ASI Resolution laser ablation system coupled to a Thermo Scientific Element 2 single collector magnetic sector field inductively coupled plasma mass spectrometer (SC-SF-ICP-MS). Over the duration of ablation, groups of 10–12 zircon grains were analyzed, followed by at least two analyses each of a primary (GJ-1, 609 Ma 66 and secondary in-house zircon standard (Pleasovice 337.13 ± 0.37. Data reduction was conducted via Iolite. If grains exhibited a greater discordance of 15%, those grains were omitted from the populations and the study as a whole. All standard analyses were within 2% of the expected ages, and most were within 1% of the expected age. Youngest maximum depositional age was calculated by combining the results for YSG, YDZ, YC1s [+3], weight average, YC2s [+3], and TuffZirc [+6])

☒ Tick this box to confirm that the raw and calibrated dates are available in the paper or in Supplementary Information.
